# Supplementary material for: Photogated two conductive pathways of donor-acceptor Stenhouse adducts in single-molecule junctions
Source: Nat Commun. 2026 Feb 16;17:2842. doi: 10.1038/s41467-026-69459-0 (PMC13022350; doi:10.1038/s41467-026-69459-0)
Supplement: Supplementary file 1 — Supplementary Information [file 41467_2026_69459_MOESM1_ESM.pdf]

Supplementary Information for

Photogated Two Conductive Pathways of Donor-Acceptor Stenhouse Adducts in Single-Molecule Junctions

Fanxi Sun[1, #], Shengqing Jiang[1, #], Hanjun Zhang[1, 2, #], Rui Wang[3]*, Yu Ji[1], Songjun Hou[4], Maolin Zhang[1], Gaolu Zhu[1], Tianfang Shi[1], Jiayu Li[1], Yuantao Zheng[1], Wenshu Liu[1], Yangyang Pan[1], Hao Luo[1], Xu Deng[5], Yonghao Zheng[1]*, Chen Wei[1] and Dongsheng Wang[1]*

[1]School of Optoelectronic Science and Engineering, University of Electronic Science and Technology of China, Chengdu 610054, China.

[2]School of Pharmacy, Qujing University of Medicine & Health Sciences, Qujing 655100, China.

[3]School of Chemistry and Molecular Engineering, East China University of Science and Technology, Shanghai 200237, China.

[4]Department of Physics, Lancaster University, Lancaster LA1 4YB, UK.

[5]Institute of Fundamental and Frontier Sciences, University of Electronic Science and Technology of China, Chengdu 610054, China.

[#]These authors contributed equally: Fanxi Sun, Shengqing Jiang and Hanjun Zhang.

Corresponding Authors: Rui Wang (rui_wang@ecust.edu.cn), Yonghao Zheng (zhengyonghao@uestc.edu.cn) and Dongsheng Wang (wangds@uestc.edu.cn).

Table of Contents

1 Synthesis and Characterization 3

2 Computational analysis of the DASAs photoisomerization pathway 9

3 Photoisomerization property of DASAs 13

4 STM-BJ measurements 17

4.1 Background conductance measurements 18

4.2 DAS and SDA (single anchoring site) 18

4.3 SSDA and SDAS (two anchoring sites) 24

4.4 SSDAS (three anchoring sites) 29

5 Electron transport calculations 32

6 The isomerization characteristics 36

7 NMR and MS spectra 39

8 Cartesian coordinates 51

9 References 107

1 Synthesis and Characterization

DASAs bearing one, two, or three thioether anchoring groups were synthesized in two steps. First, five modular intermediates (1-5) were prepared. These intermediates were then subjected to similar aza-Piancatelli rearrangement reactions to afford DASAs with different numbers of thioether anchoring groups.

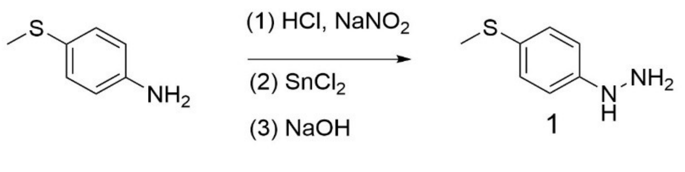

Supplementary Fig. 1 | Synthesis of Intermediate 1

Synthesis of Intermediate 1. Intermediate 1 was synthesized according to the method reported by Nichols, Richard J et al[1]. 4-(Methylthio)aniline (3.48 g, 25.0 mmol) was added to hydrochloric acid solution (37%, 12 M, 32.0 mL) and cooled to 0 ºC. Aqueous sodium nitrite (1.37 g, 19.8 mmol in 16.0 mL H_2O) was added dropwise under the same temperature. After stirring for 30 min, a solution of tin(II) chloride dihydrate (12.8 g, 56.6 mmol) in hydrochloric acid (37%, 13.0 mL) was added. The mixture was stirred for 3 h, and the resulting precipitate was collected by filtration. The solid was dispersed in sodium hydroxide aqueous solution (1.0 M, 20 mL) and stirred for 1 h. After extraction with DCM and rotary evaporation, **Intermediate 1** was obtained as a brownish oil (2.69 g, 70%).

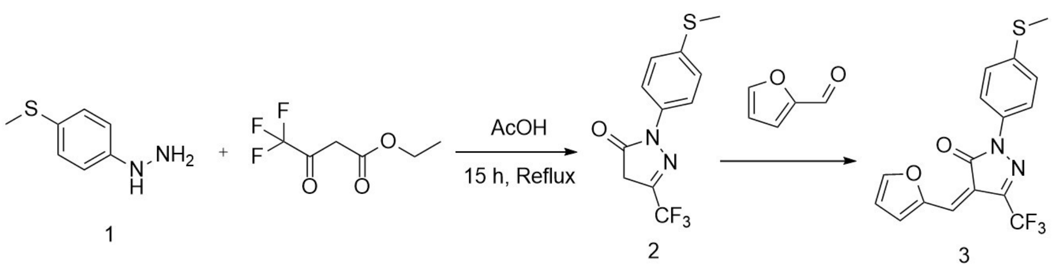

Supplementary Fig. 2 | Synthesis of Intermediate 3

Synthesis of Intermediate 3. Intermediate 3 was synthesized via a modified procedure reported by Javier Read de Alaniz et al[2]. Intermediate 1 (3.7 g, 24 mmol, 1.0 eq.) and ethyl 4,4,4-trifluoroacetoacetate (3.5 mL, 24 mmol, 1.0 eq.) were dissolved in glacial acetic acid (6 mL) and

refluxed under nitrogen for 15 h. After cooling to room temperature, beige crystals formed and were collected by filtration, washed with water (10 mL), and dried under vacuum overnight. The resulting crude product (Intermediate 2) was used directly in the next step. The crude product (3.02 g, 11 mmol, 1.0 eq.) and furfural (1.92 g, 20 mmol, 1.8 eq.) were dissolved in dichloromethane (30 mL) and stirred at 25 °C for 20 h. The reaction mixture was filtered through a silica column using DCM as eluent. Water (20 mL) was added to the orange filtrate, and the organic solvent was removed by rotary evaporation. The resulting precipitate was collected by filtration and washed with water to yield **Intermediate 3** as a deep orange solid (2.88 g, 9.2 mmol, 74%).

^{1}H NMR (400 MHz, $CDCl_3$) δ (ppm): 8.92 (d, J = 3.9 Hz, 1H), 7.88 (d, J = 7.9 Hz, 2H), 7.84 (d, J = 7.9 Hz, 1H), 7.69 (s, 1H), 7.33 (d, J = 8.4 Hz, 2H), 6.81 (t, J = 8.0 Hz, 1H), 2.51 (s, 3H).

^{13}C NMR (101 MHz, $CDCl_3$) δ (ppm): 160.17, 149.73, 139.24, 138.99, 138.74, 138.49, 135.03, 134.04, 130.57, 127.00, 126.20, 119.73, 119.20, 117.93, 114.63, 15.17.

HRMS (ESI$^+$): *m/z* found 352.0475, 353.0532, 354.0542; calcd for $C_{16}H_{11}F_3N_2O_2S$ [**M**]$^+$ 352.0493 (100%), 353.0527 (17.3%), 354.0451 (4.5%), 354.0560 (1.4%).

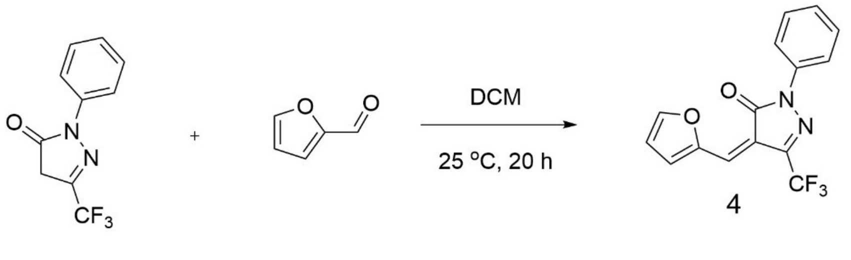

Supplementary Fig. 3 | Synthesis of Intermediate 4

Synthesis of Intermediate 4. Intermediate 4 was synthesized in a similar manner to Intermediate 3. 3-trifluoromethyl-1-phenyl-1H-5-Pyrazolone (2.51 g, 11 mmol) and furfural (1.92 g, 20 mmol, 1.8 eq.) were stirred in DCM (30 mL) at 25 °C for 20 h. The mixture was filtered through a silica column (DCM eluent). Water (20 mL) was added to the filtrate, and the solvent was removed. The precipitated product was filtered and washed with water to afford Intermediate 4 as a deep orange solid (2.56 g, 8.36 mmol, 76%).

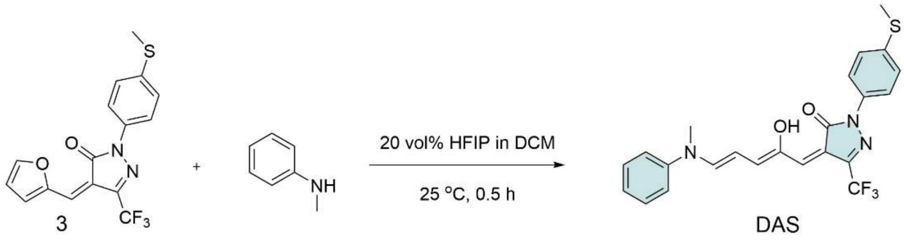

Supplementary Fig. 4 | Synthesis of DAS

Synthesis of DAS. Intermediate 3 (112.75 mg, 0.32 mmol, 1.0 eq.) and N-methylaniline (42.86 mg, 0.40 mmol, 1.25 eq.) were dissolved in DCM (0.8 mL), and hexafluoroisopropanol (HFIP, 0.2 mL) was added. The mixture was stirred for 0.5 h. After removing the solvent under reduced pressure, the residue was triturated with diethyl ether (1.0 mL). The crude solid was dissolved in THF to form a saturated solution, which was then poured into cold hexane. The precipitation–redissolution cycle was repeated three times. The final solid was dried under vacuum at 60 °C to give **DAS** as a dark blue-green solid (56.32 mg, 0.12 mmol, 38%).

^{1}H NMR (400 MHz, DMSO-*d6*) δ (ppm): 11.55 (s, 1H), 7.87 (dd, *J* = 6.0, 2.0 Hz, 1H), 7.58-7.50 (m, 2H), 7.43-7.35 (m, 2H), 7.15-7.05 (m, 2H), 6.72 (d, *J* = 8.3 Hz, 2H), 6.64 (t, *J* = 7.2 Hz, 1H), 6.52 (dd, *J* = 6.0, 2.0 Hz, 1H), 5.28-5.22 (m, 1H), 3.77 (d, *J* = 3.9 Hz, 1H), 2.78 (s, 3H), 2.52 (s, 3H).

^{13}C NMR (101 MHz, DMSO-*d6*) δ (ppm): 204.16, 163.64, 151.84, 149.71, 138.54, 138.28, 134.92, 134.45, 129.31, 126.76, 125.99, 123.67, 123.31, 120.63, 117.95, 114.01, 98.55, 66.85, 45.20, 40.62, 40.41, 32.79, 15.16.

HRMS (ESI$^+$): *m/z* found 460.1313 ([**M+H**]$^+$), 482.1133 ([**M+Na**]$^+$), 498.0871([**M+K**]$^+$); calcd for $C_{23}H_{20}F_3N_3O_2S$ [**M+H**]$^+$ 460.1201, [**M+Na**]$^+$ 482.1020, [**M+K**]$^+$ 498.0759.

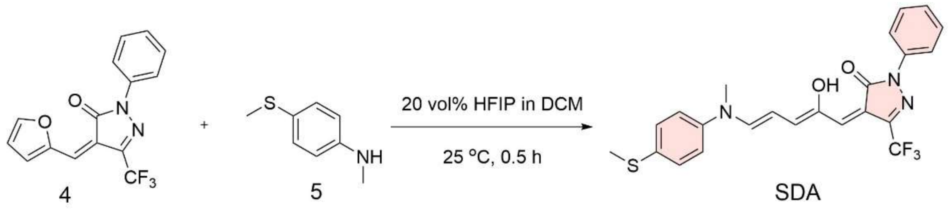

Supplementary Fig. 5 | Synthesis of SDA

Synthesis of SDA. Intermediate 4 (98.00 mg, 0.32 mmol) and N-methyl-4-(methylsulfanyl)aniline (61.30 mg, 0.40 mmol, 1.25 eq.) were dissolved in DCM (0.8 mL) and stirred with HFIP (0.2 mL) for 0.5 h. After solvent removal, the residue was triturated with ether and recrystallized from THF/hexane as described above to yield SDA as a dark blue-green solid (97.00 mg, 0.21 mmol, 66%).

^{1}H NMR (400 MHz, DMSO-*d6*) δ (ppm): 11.54 (s, 1H), 7.87 (dd, *J* = 5.9, 2.0 Hz, 1H), 7.59 (d, *J* = 7.7 Hz, 2H), 7.51 (dd, *J* = 8.6, 7.0 Hz, 2H), 7.40 (t, *J* = 7.3 Hz, 1H), 7.08 (d, *J* = 8.7 Hz, 2H), 6.70 (s, 2H), 6.52 (dd, *J* = 5.9, 2.0 Hz, 1H), 5.22 (s, 1H), 3.75 (d, *J* = 3.8 Hz, 1H), 2.76 (s, 3H), 2.31 (s, 3H).

^{13}C NMR (101 MHz, DMSO-*d6*) δ (ppm): 204.12, 163.43, 152.83, 148.14, 137.99, 134.51, 129.80, 129.64, 128.14, 125.05, 123.24, 114.95, 45.08, 40.63, 40.42, 40.21, 40.00, 39.79, 39.58, 39.37, 32.92, 25.90, 17.58.

HRMS (ESI$^+$): *m/z* found 460.1306 ([**M+H**]$^+$), 482.1124 ([**M+Na**]$^+$), 498.0864 ([**M+K**]$^+$); calcd for $C_{23}H_{20}F_3N_3O_2S$ [**M+H**]$^+$ 460.1201, [**M+Na**]$^+$ 482.1020, [**M+K**]$^+$ 498.0759.

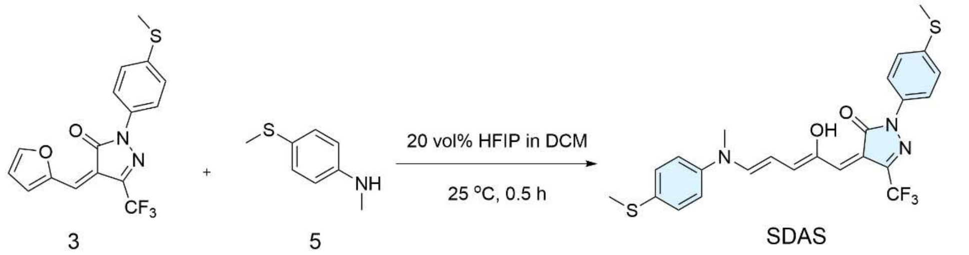

Supplementary Fig. 6 | Synthesis of SDAS

Synthesis of SDAS. Intermediate 3 (112.75 mg, 0.32 mmol) and Intermediate 5 (61.30 mg, 0.40 mmol, 1.25 eq.) were treated under the same conditions as SDA to afford SDAS as a dark blue-green solid (79.21 mg, 0.16 mmol, 48%).

^{1}H NMR (400 MHz, DMSO-*d6*) δ (ppm): 11.55 (s, 1H), 7.87 (dd, *J* = 6.0, 2.0 Hz, 1H), 7.53 (d, *J* = 8.6 Hz, 2H), 7.44-7.35 (m, 2H), 7.14-7.04 (m, 2H), 6.73-6.65 (m, 2H), 6.52 (dd, *J* = 5.9, 2.0 Hz, 1H), 5.22-5.18 (m, 1H), 3.74 (d, *J* = 3.8 Hz, 1H), 2.76 (s, 3H), 2.32 (s, 3H).

^{13}C NMR (101 MHz, DMSO-*d6*) δ (ppm): 204.08, 163.44, 148.13, 138.28, 134.91, 134.51, 129.80, 126.76, 125.05, 123.70, 123.30, 114.93, 67.02, 45.09, 40.62, 40.41, 40.21, 40.00, 39.79, 39.58, 39.37, 32.92, 17.59, 15.18.

HRMS (ESI$^+$): *m/z* found 506.1161 ([**M+H**]$^+$), 528.0980 ([**M+Na**]$^+$), 544.0719 ([**M+K**]$^+$); calcd for $C_{24}H_{22}F_3N_3O_2S_2$ [**M+H**]$^+$ 506.1184, [**M+Na**]$^+$ 528.1003, [**M+K**]$^+$ 544.0743.

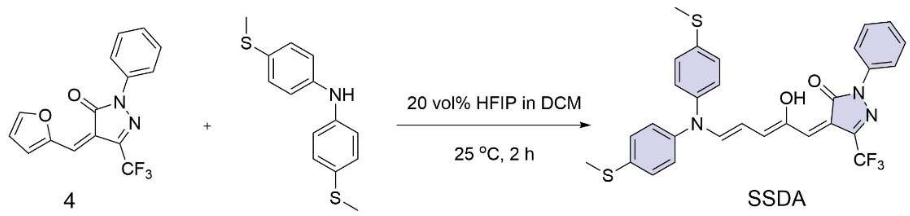

Supplementary Fig. 7 | Synthesis of SSDA

Synthesis of SSDA. Intermediate 4 (98.00 mg, 0.32 mmol) and 4,4'-dimethylthiodiphenylamine (104.56 mg, 0.40 mmol, 1.25 eq.) were reacted under the same procedure as above, except that the stirring time in HFIP was extended to 2 h. The resulting solid SSDA was obtained as a dark blue-green solid (79.21 mg, 0.12 mmol, 48%).

^{1}H NMR (400 MHz, DMSO-*d6*) δ (ppm): 11.60 (s, 1H), 8.09 (dd, *J* = 6.0, 2.0 Hz, 1H), 7.71-7.64 (m, 2H), 7.55 (dd, *J* = 8.7, 7.1 Hz, 2H), 7.49-7.40 (m, 1H), 7.24-7.15 (m, 4H), 6.86-6.78 (m, 4H), 6.39 (dd, *J* = 5.9, 2.0 Hz, 1H), 5.45 (s, 1H), 3.68 (d, *J* = 3.9 Hz, 1H), 2.41 (s, 6H).

^{13}C NMR (101 MHz, DMSO-*d6*) δ (ppm): 203.93, 163.31, 152.11, 143.65, 141.89, 137.93, 133.80, 131.88, 129.74, 129.63, 128.53, 128.26, 127.50, 123.61, 123.43, 123.35, 120.67, 117.97, 99.21, 65.88, 46.30, 40.62, 40.41, 40.00, 39.79, 39.58, 39.37, 17.25, 16.17.

HRMS (ESI$^+$): *m/z* found 568.1305 ([**M+H**]$^+$), 590.1122 ([**M+Na**]$^+$), 606.0863 ([**M+K**]$^+$); calcd for $C_{29}H_{24}F_3N_3O_2S_2$ [**M+H**]$^+$ 568.1340, [**M+Na**]$^+$ 590.1160, [**M+K**]$^+$ 606.0899.

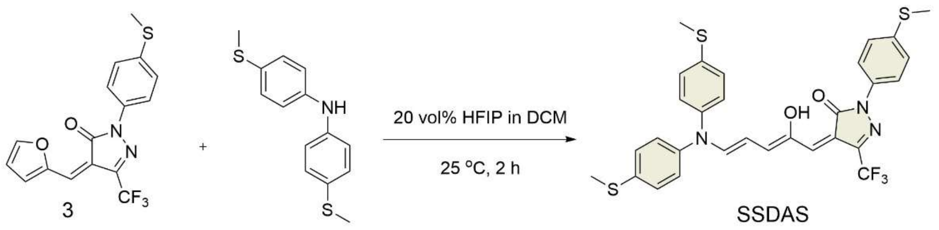

Supplementary Fig. 8 | Synthesis of SSDAS

Synthesis of SSDAS. Intermediate 3 (112.75 mg, 0.32 mmol) and 4,4'-dimethylthiodiphenylamine (104.56 mg, 0.40 mmol, 1.25 eq.) were reacted following the same procedure to yield SSDAS as a dark blue-green solid (74.32 mg, 0.12 mmol, 38%).

^{1}H NMR (400 MHz, DMSO-*d6*) δ (ppm): 11.62 (s, 1H), 8.08 (dd, *J* = 5.9, 2.0 Hz, 1H), 7.65-7.58 (m, 2H), 7.45-7.37 (m, 2H), 7.24-7.15 (m, 4H), 6.85-6.77 (m, 4H), 6.38 (dd, *J* = 6.0, 2.0 Hz, 1H), 5.45 (s, 1H), 3.68-3.57 (m, 1H), 2.53 (s, 3H), 2.41 (s, 6H).

^{13}C NMR (101 MHz, DMSO-*d6*) δ (ppm): 207.24, 193.26, 163.32, 165.52, 143.65, 138.33, 134.88 133.80, 131.87, 128.53, 126.82, 126.54, 123.79, 123.61, 109.24, 83.81, 76.22, 68.50, 60.07, 46.30, 40.63, 40.42, 40.21, 40.00, 39.79, 39.58, 39.37, 16.18, 15.20, 14.95.

HRMS (ESI$^+$): *m/z* found 614.1168 ([**M+H**]$^+$), 636.0985 ([**M+Na**]$^+$), 652.0726 ([**M+K**]$^+$); calcd for $C_{30}H_{26}F_3N_3O_2S_3$ [**M+H**]$^+$ 614.1321, [**M+Na**]$^+$ 636.1141, [**M+K**]$^+$ 652.0880.

2 Computational analysis of the DASAs photoisomerization pathway

All quantum chemical calculations were performed using the Gaussian 16 software package[3]. Geometry optimizations for all species were conducted using density functional theory (DFT) at the M06-2X/6-311G(d) level, including frequency analyses to confirm the nature of the stationary points[4,5]. Molecular geometries and dimensions were extracted from optimized structures. Electrostatic potential (ESP) distributions were analyzed using Multiwfn 6.0[6] and visualized with VMD[7].The molecular polarity index (*MPI*) was evaluated based on the calculated ESP distributions using Multiwfn 6.0[6].

Transition state (TS) searches were performed starting from the initial isomer (A) by relaxed potential energy surface (PES) scans along the reaction coordinate (e.g., dihedral angles for *Z/E* isomerization)[8,9]. The highest-energy geometries along the scan paths were used as starting points for TS optimization, employing the keyword opt = (ts, calcfc, noeigentest, gdiis).

All optimizations and single-point energy calculations were carried out at the M06-2X/6-311G(d) level with Grimme's D3 dispersion correction[4,5]. Solvent effects were considered using the SMD implicit solvation model, with 1,2,4-trichlorobenzene (TCB, $\varepsilon = 2.25$) as the solvent.

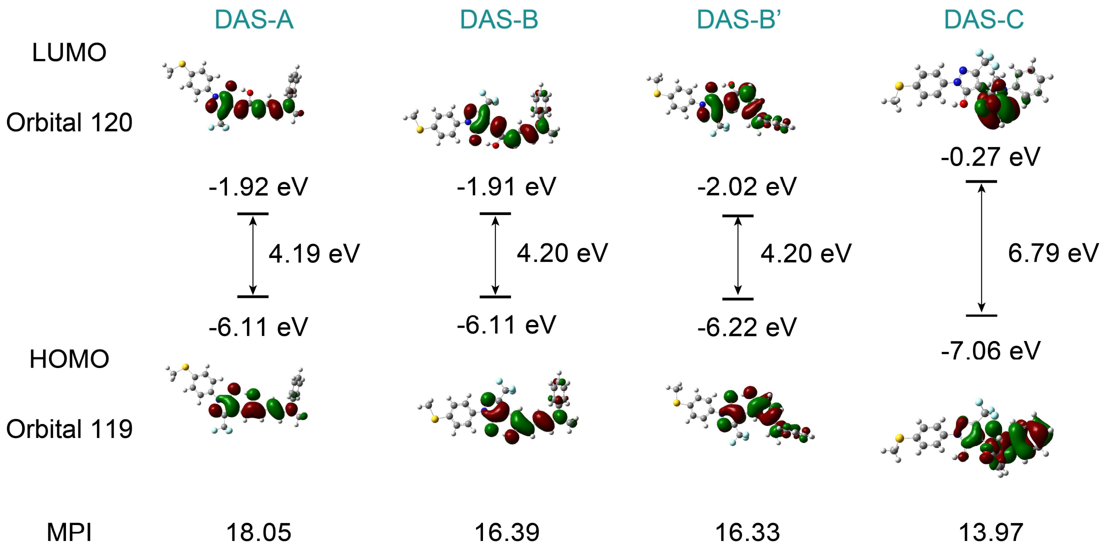

Supplementary Fig. 9 | Frontier orbitals, energy gaps and molecular polarity index of DAS. HOMO and LUMO distributions, orbital energies, HOMO-LUMO energy gaps and MPI values of DAS-A, DAS-B, DAS-B′ and DAS-C calculated at the M06-2X/6-311G(d) level with Grimme's D3 dispersion correction.

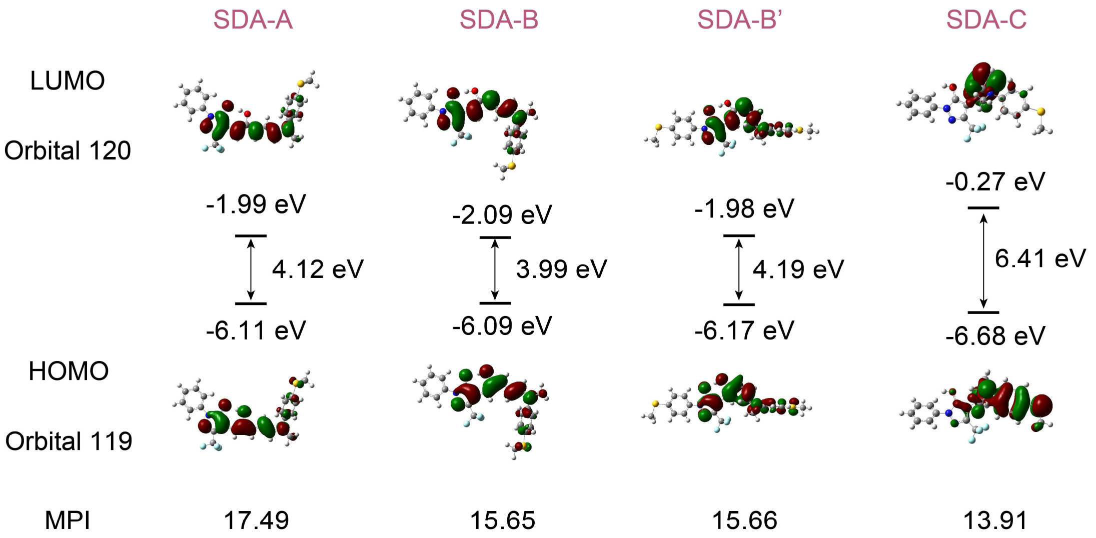

Supplementary Fig. 10 | Frontier orbitals, energy gaps and molecular polarity index of SDA. HOMO and LUMO distributions, orbital energies, HOMO-LUMO energy gaps and MPI values of SDA-A, SDA-B, SDA-B′ and SDA-C calculated at the M06-2X/6-311G(d) level with Grimme's D3 dispersion correction.

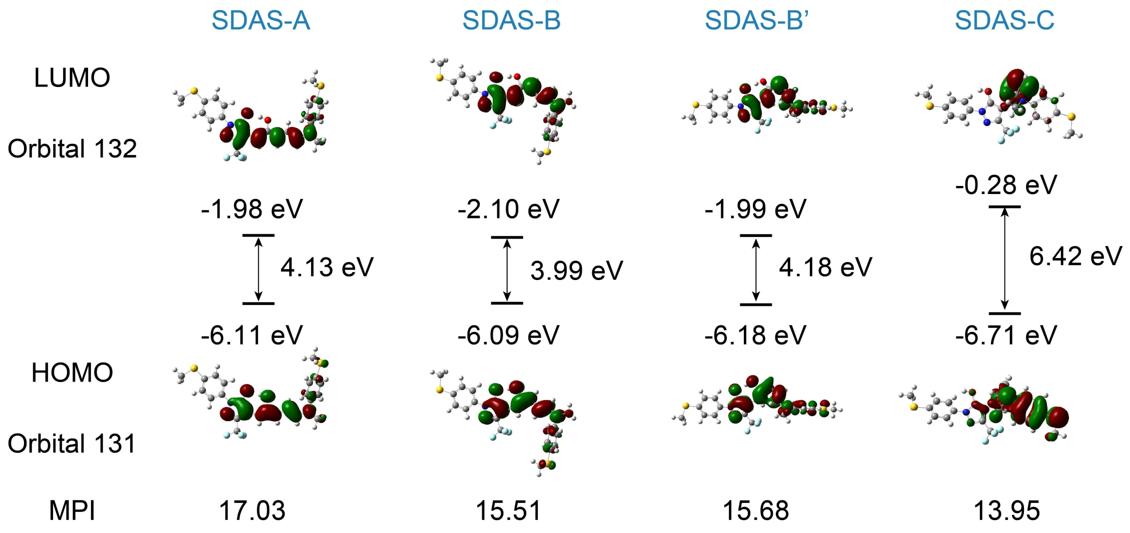

Supplementary Fig. 11 | Frontier orbitals, energy gaps and molecular polarity index of SDAS. HOMO and LUMO distributions, orbital energies, HOMO-LUMO energy gaps and MPI values of SDAS-A, SDAS-B, SDAS-B′ and SDAS-C calculated at the M06-2X/6-311G(d) level with Grimme’s D3 dispersion correction.

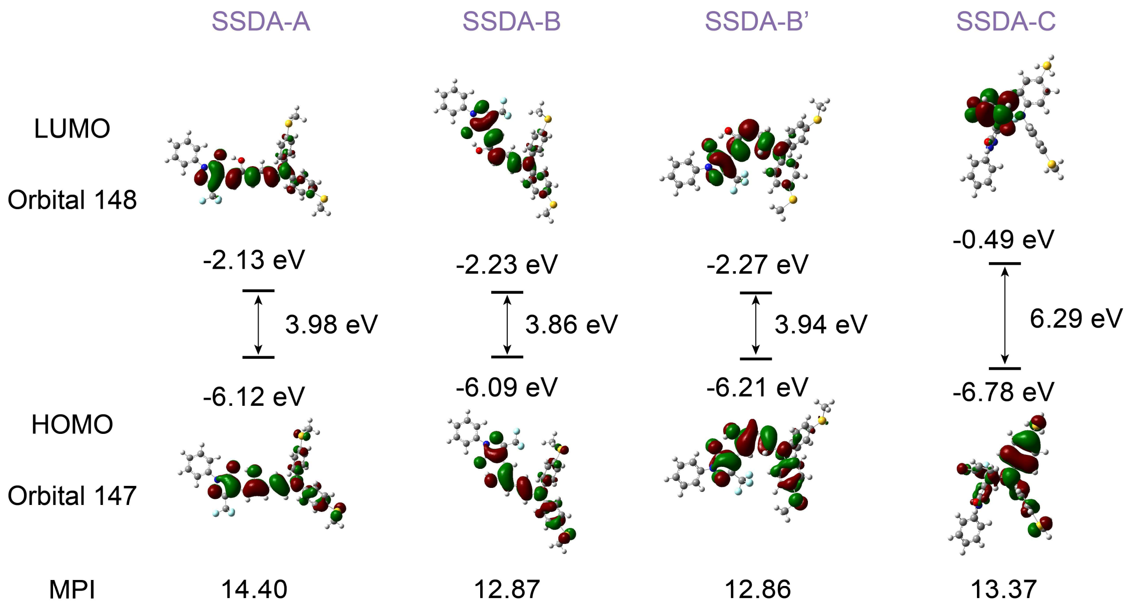

Supplementary Fig. 12 | Frontier orbitals, energy gaps and molecular polarity index of SDAS. HOMO and LUMO distributions, orbital energies, HOMO-LUMO energy gaps and MPI values of SDAS-A, SDAS-B, SDAS-B′ and SDAS-C calculated at the M06-2X/6-311G(d) level with Grimme’s D3 dispersion correction.

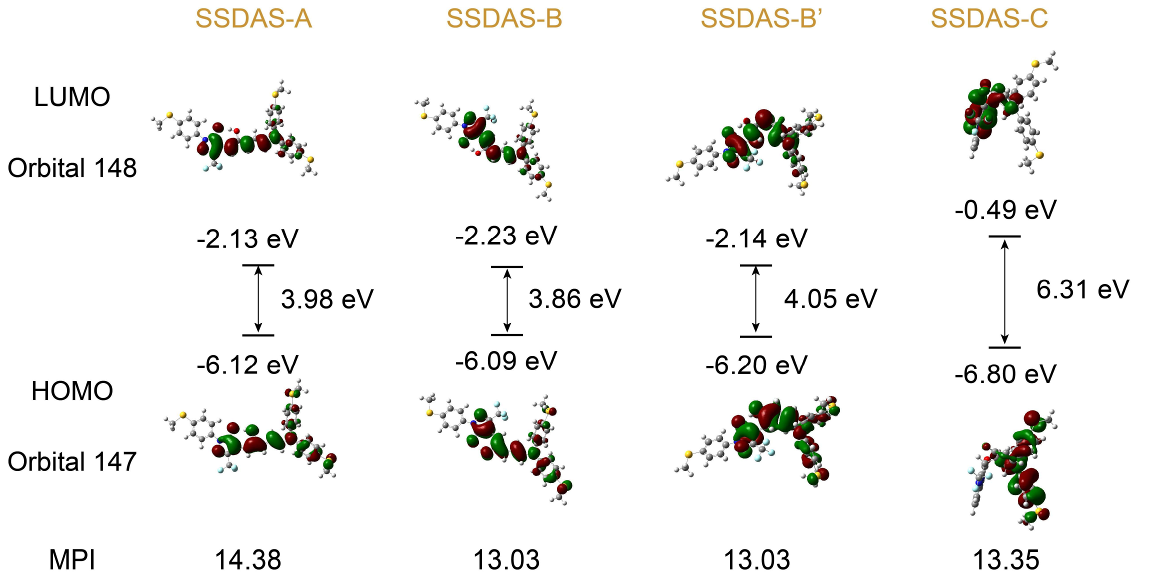

Supplementary Fig. 13 | Frontier orbitals, energy gaps and molecular polarity index of SSDAS. HOMO and LUMO distributions, orbital energies, HOMO-LUMO energy gaps and MPI values of SSDAS-A, SSDAS-B, SSDAS-B′ and SSDAS-C calculated at the M06-2X/6-311G(d) level with Grimme’s D3 dispersion correction.

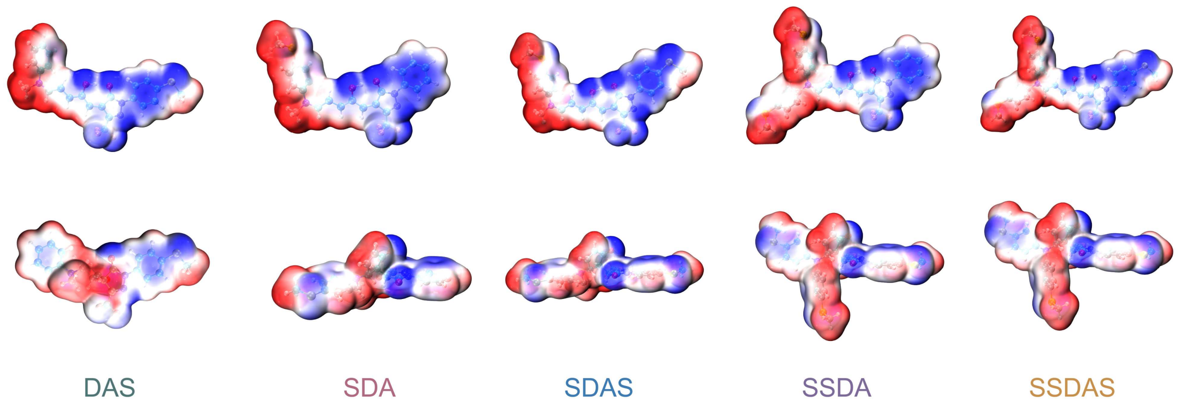

Supplementary Fig. 14 | Electrostatic potential (ESP) maps of DASAs derivatives in *linear* and *cyclic* states. ESP distributions of DAS, SDA, SDAS, SSDA, and SSDAS in their *linear* (top) and *cyclic* (bottom) isomers. Color scale represents electrostatic potential mapped onto the electron density isosurface (0.001 a.u.), highlighting charge redistribution upon photoisomerization. The MPI values are evaluated based on the calculated ESP distributions using Multiwfn 6.0.

3 Photoisomerization property of DASAs

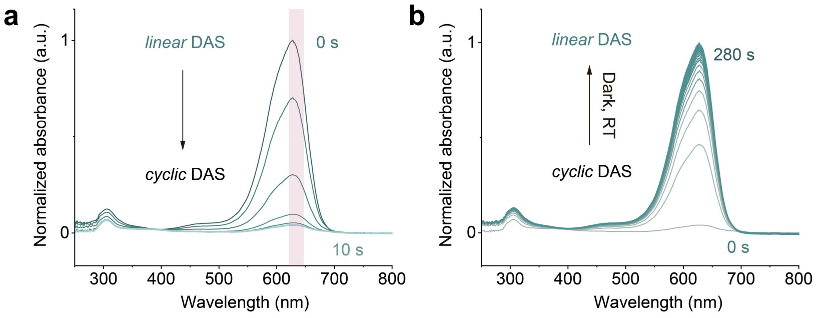

Supplementary Fig. 15 | Photoisomerization and thermal recovery kinetics of DAS in solution. a Time-dependent UV-vis absorption spectra of DAS (0.025 mM in TCB) under continuous irradiation at 635 nm. **b** Thermal recovery of DAS monitored by UV-vis absorption spectra in the dark at 20 °C after irradiation.

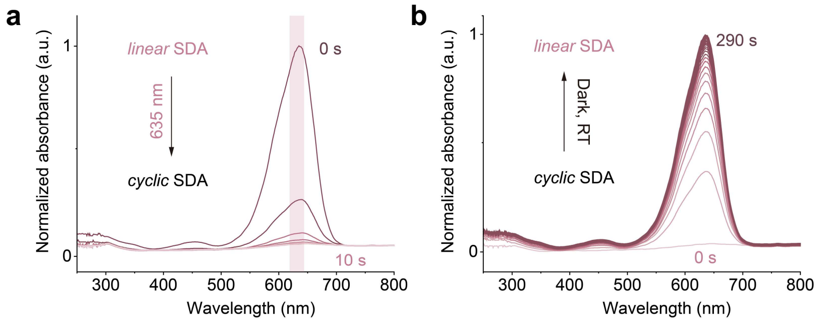

Supplementary Fig. 16 | Photoisomerization and thermal recovery kinetics of SDA in solution. a Time-dependent UV-vis absorption spectra of SDA (0.025 mM in TCB) under continuous irradiation at 635 nm. **b** Thermal recovery of SDA monitored by UV-vis absorption spectra in the dark at 20 °C after irradiation.

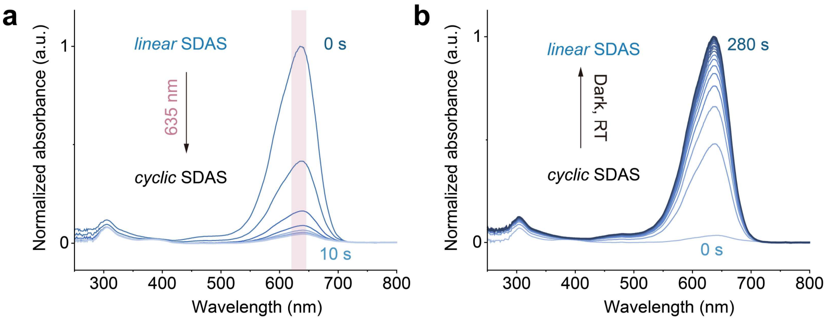

Supplementary Fig. 17 | Photoisomerization and thermal recovery kinetics of SDAS in solution. a Time-dependent UV-vis absorption spectra of SDAS (0.025 mM in TCB) under continuous irradiation at 635 nm. **b** Thermal recovery of SDAS monitored by UV-vis absorption spectra in the dark at 20 °C after irradiation.

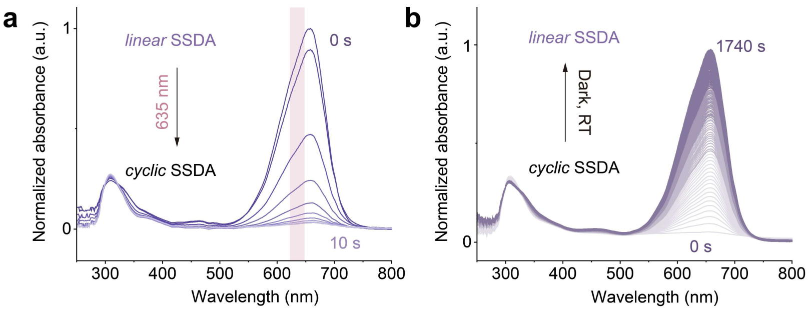

Supplementary Fig. 18 | Photoisomerization and thermal recovery kinetics of SSDA in solution. a Time-dependent UV-vis absorption spectra of SSDA (0.025 mM in TCB) under continuous irradiation at 635 nm. **b,** Thermal recovery of SSDA monitored by UV-vis absorption spectra in the dark at 20 °C after irradiation.

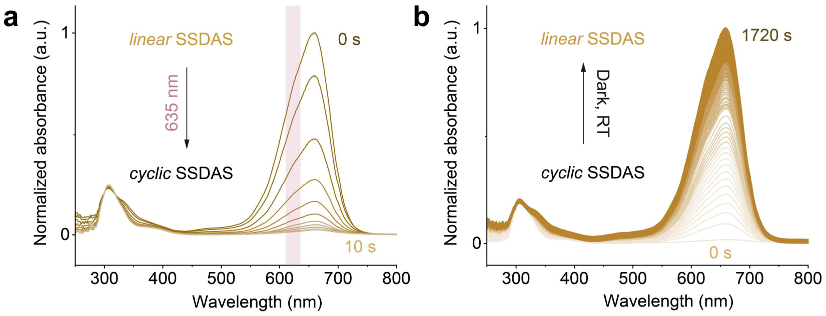

Supplementary Fig. 19 | Photoisomerization and thermal recovery kinetics of SSDAS in solution. a Time-dependent UV-vis absorption spectra of SSDAS (0.025 mM in TCB) under continuous irradiation at 635 nm. **B** Thermal recovery of SSDAS monitored by UV-vis absorption spectra in the dark at 20 °C after irradiation.

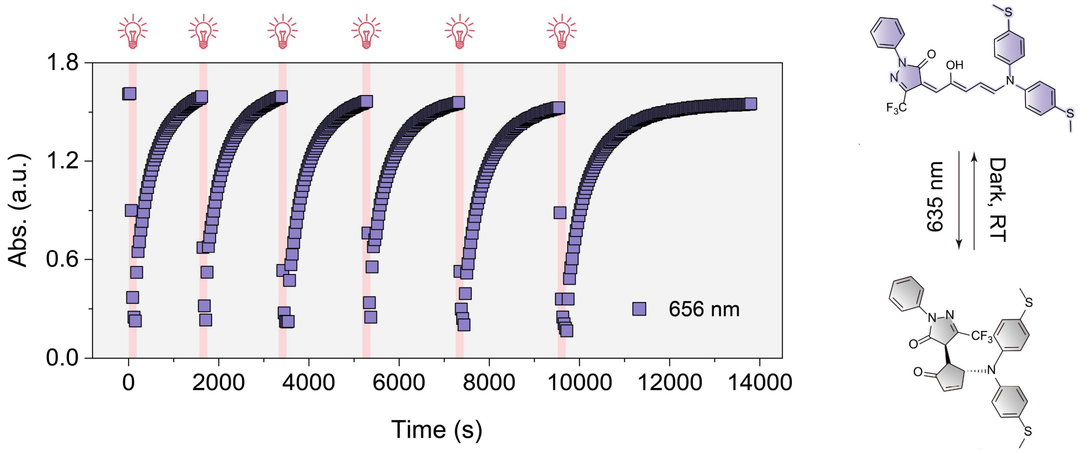

Supplementary Fig. 20 | Reversible photoswitching cycles of SSDA. Fatigue resistance of SSDA under repeated *linear*-to-*cyclic* photoisomerization (635 nm laser, 1 mW cm^{-2}) and *cyclic*-to-*linear* thermal relaxation (19 ^{o}C, dark). UV-vis absorbance at 656 nm was monitored over multiple switching cycles.

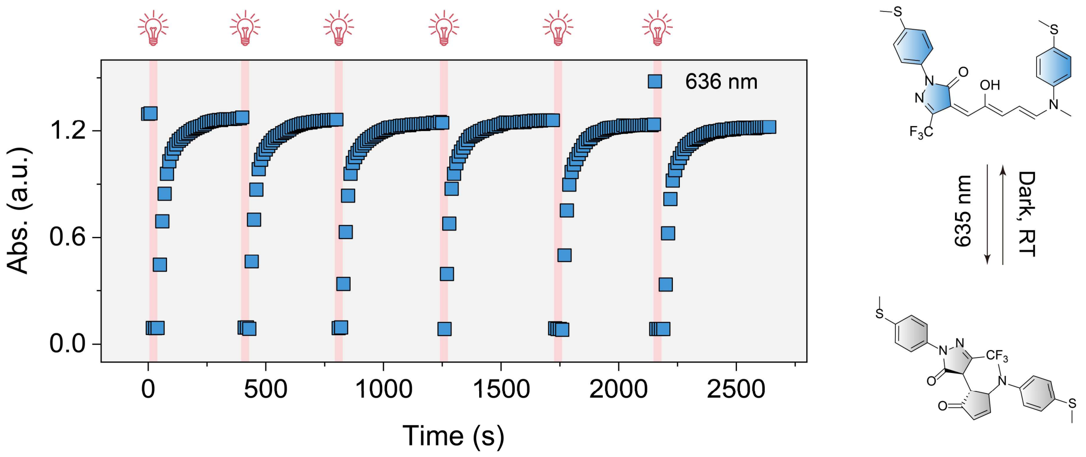

Supplementary Fig. 21 | Reversible photoswitching cycles of SDAS. Fatigue resistance of SDAS under repeated *linear*-to-*cyclic* photoisomerization (635 nm laser, 1 mW cm^{-2}) and *cyclic*-to-*linear* thermal relaxation (19 ^{o}C, dark). UV-vis absorbance at 636 nm was monitored over multiple switching cycles.

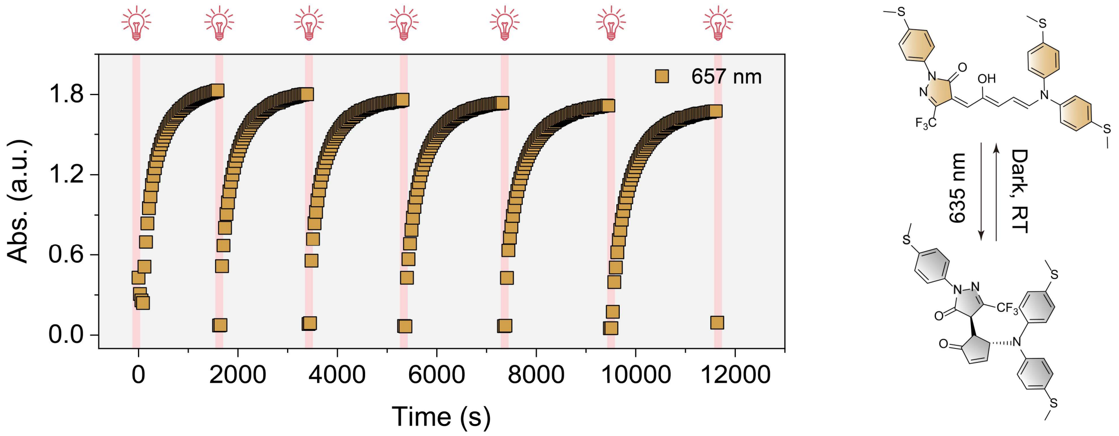

Supplementary Fig. 22 | Reversible photoswitching cycles of SSDAS. Fatigue resistance of SSDAS under repeated *linear*-to-*cyclic* photoisomerization (635 nm laser, 1 mW cm^{-2}) and *cyclic*-to-*linear* thermal relaxation (19 ^{o}C, dark). UV-vis absorbance at 657 nm was monitored over multiple switching cycles.

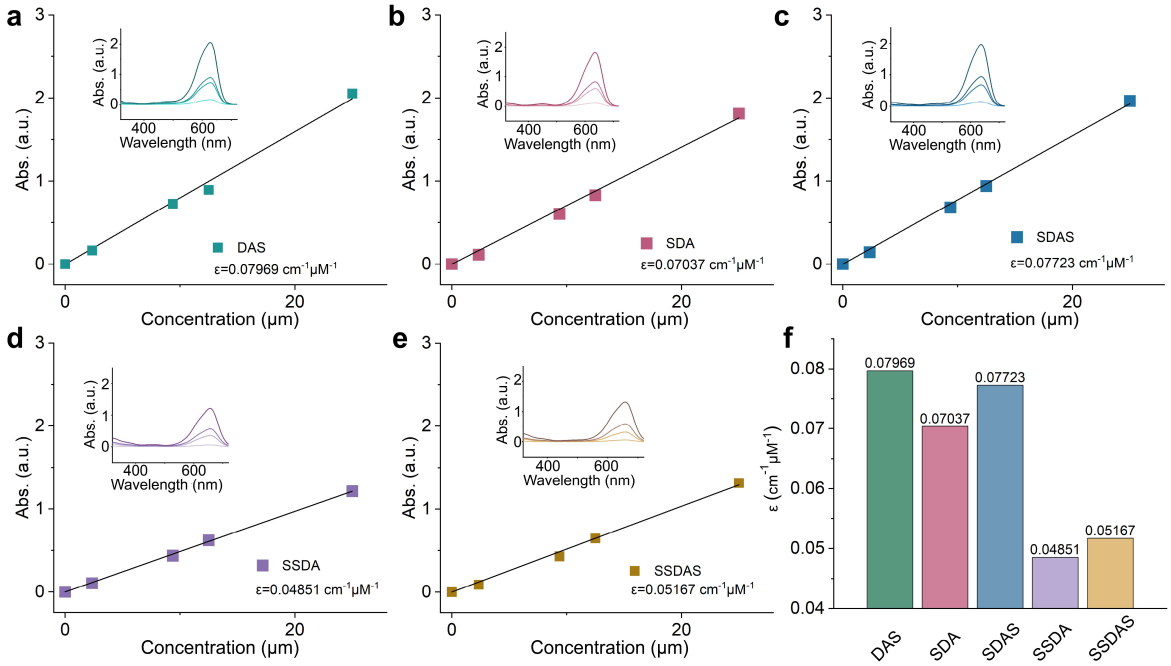

Supplementary Fig. 23 | Molar absorption coefficients. a-e Absorbance-concentration calibration curves and corresponding UV-vis absorption spectra (insets) for DAS, SDA, SDAS, SSDA, and SSDAS, respectively. Linear fitting yields molar absorption coefficients (ε) as indicated. **f** Comparison of ε values for the five compounds.

4 STM-BJ measurements

Single-molecule conductance measurements were performed using a home-built scanning tunneling microscope break-junction (STM-BJ) setup. Gold tips (99.99%, 0.25 mm) were fabricated by melting Au wire into a bead and mounted on a piezoelectric actuator fixed to a stepping motor for coarse control. Gold-coated silicon wafers were used as substrates, cleaned with piranha solution (H_2SO_4/H_2O_2 = 3:1, v/v), rinsed with ultrapure water (18.25 MΩ·cm) and dried in vacuum prior to each experiment.

5-10 μL of a 0.1 mM solution of the DASAs in anhydrous 1,2,4-trichlorobenzene (TCB) was deposited on the substrate. A 635 nm continuous-wave laser (K635EWDFN-5.000W, BWT Beijing) was employed as the monochromatic light source for photophysical and isomerization studies. The laser output was coupled into a quartz optical fiber to allow flexible alignment and precise delivery of light to the sample. The fiber tip was positioned to the substrate surface, ensuring uniform irradiation over the entire junction area. The laser intensity (1 mW/cm^2) at the sample surface was monitored using a calibrated power meter (PcPlug V3, LaserPoint). All photoirradiation experiments were performed under ambient conditions unless otherwise specified. All measurements were carried out at 298 K, with conductance measurements performed either in the dark or under 635 nm laser irradiation, as specified in the main text. During measurements, the gold tip was repeatedly brought into and out of contact with the substrate at 10 nm/s. Conductance traces were extracted by segmenting the conductance-time curves at a 50 μA threshold, yielding ~3000 data points per trace. The time axis was converted into tip displacement (Δz) using the known piezo velocity. Peaks were obtained via Gaussian fitting.

Flicker noise analysis was performed to probe the charge transport mechanism. After junction formation, the tip was held for 150 ms and conductance fluctuations were Fourier transformed to obtain power spectral densities (PSDs)[10]. Normalized PSD vs. average conductance maps were constructed from thousands of traces, and correlation exponents were extracted using 2D Gaussian fitting[11]. Exponents near 1 indicate through-bond transport, while values near 2 indicate through-space tunneling. The molecular conductance data, including typical individual conductance traces, 1D and 2D conductance-displacement histograms are analyzed with the homemade single-

molecule break junctions experimental data processing software built with Python and PyQt5, XMe DataAnalysis, which is available for research users at https://github.com/Pilab-XMU/XMe_DataAnalysis [12].

4.1 Background conductance measurements

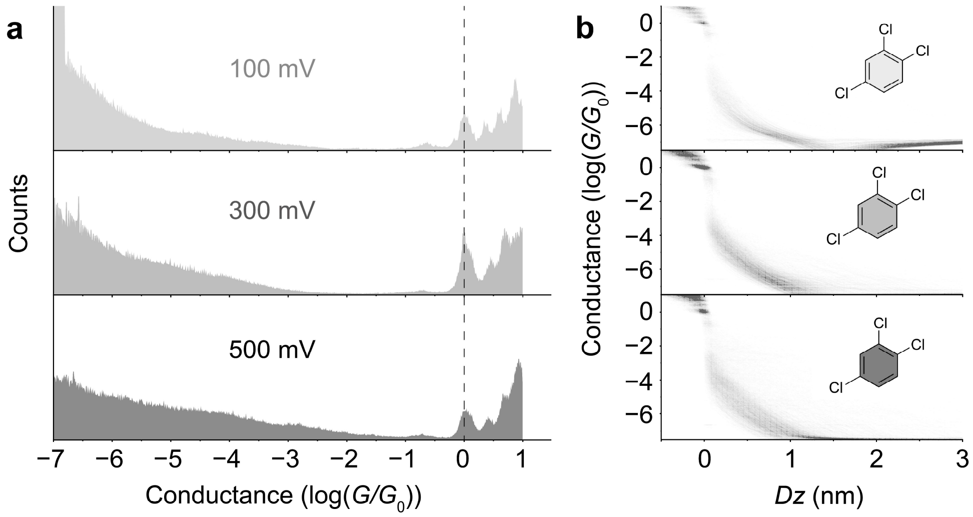

Supplementary Fig. 24 | Solvent background conductance measurements in 1,2,4-trichlorobenzene. a 1D logarithmic conductance histograms of 1,2,4-trichlorobenzene measured at bias voltages of 100 mV, 300 mV and 500 mV. **b** 2D conductance histogram of the 1,2,4-trichlorobenzene at corresponding voltages.

The data in both 1D and 2D conductance histograms suggest that 1,2,4-trichlorobenzene, as a solvent, does not exhibit any significant electronic features or conductance pathways at the applied bias voltages, which means that it serves as a background material without significantly affecting the conductance measurements of the targeted molecules.

4.2 DAS and SDA (single anchoring site)

In single-molecule conductance measurements, ensuring the clarity of the electron transport path is crucial. Apart from the typical conduction through the π-bridge or donor pathways, unconventional anchoring can introduce additional conductive paths, potentially compromising the accuracy of the measurements. In DASAs, certain atoms, such as oxygen on the π-bridge and the

acceptor part, can accidentally anchor to gold electrodes, forming unintended electron transport channels (Supplementary Fig. 26). Previous studies also suggest that intermolecular π-π stacking may create extra conductive paths, distorting the measured conductance values[13].

To confirm that the measured conductance arises from the target transport pathways, we designed SDA and DAS molecules with a single anchoring point (Fig. 1d). This design eliminates the possibility of non-typical anchoring via π-bridge oxygen, acceptor oxygen, or other sites. Ensuring that the observed conductance accurately reflects the intrinsic electron transport mechanisms.

Single-molecule conductance measurements of DAS and SDA molecules were carried out under the same conditions to rule out any interference from unconventional anchoring. For *linear* DAS, a minor conductance peak was observed around $\log(G/G_0) = -4$ (Supplementary Fig. 27), but this is not further discussed here as the primary goal of the experiment was to verify the origin of the conductance. Furthermore, SDA were tested under the same conditions. The results of the 2D conductance histograms (Supplementary Figs. 29-30) confirmed that, no significant conductance signals behave similar to donor and π-bridge pathways were observed in both the *linear* and *cyclic* states.

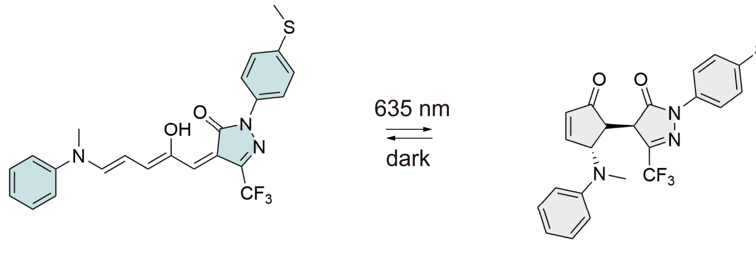

Supplementary Fig. 25 | Chemical structure of DAS. Chemical structure of DAS in its *linear* state (left) and *cyclic* state (right) upon 635 nm light irradiation and in the dark.

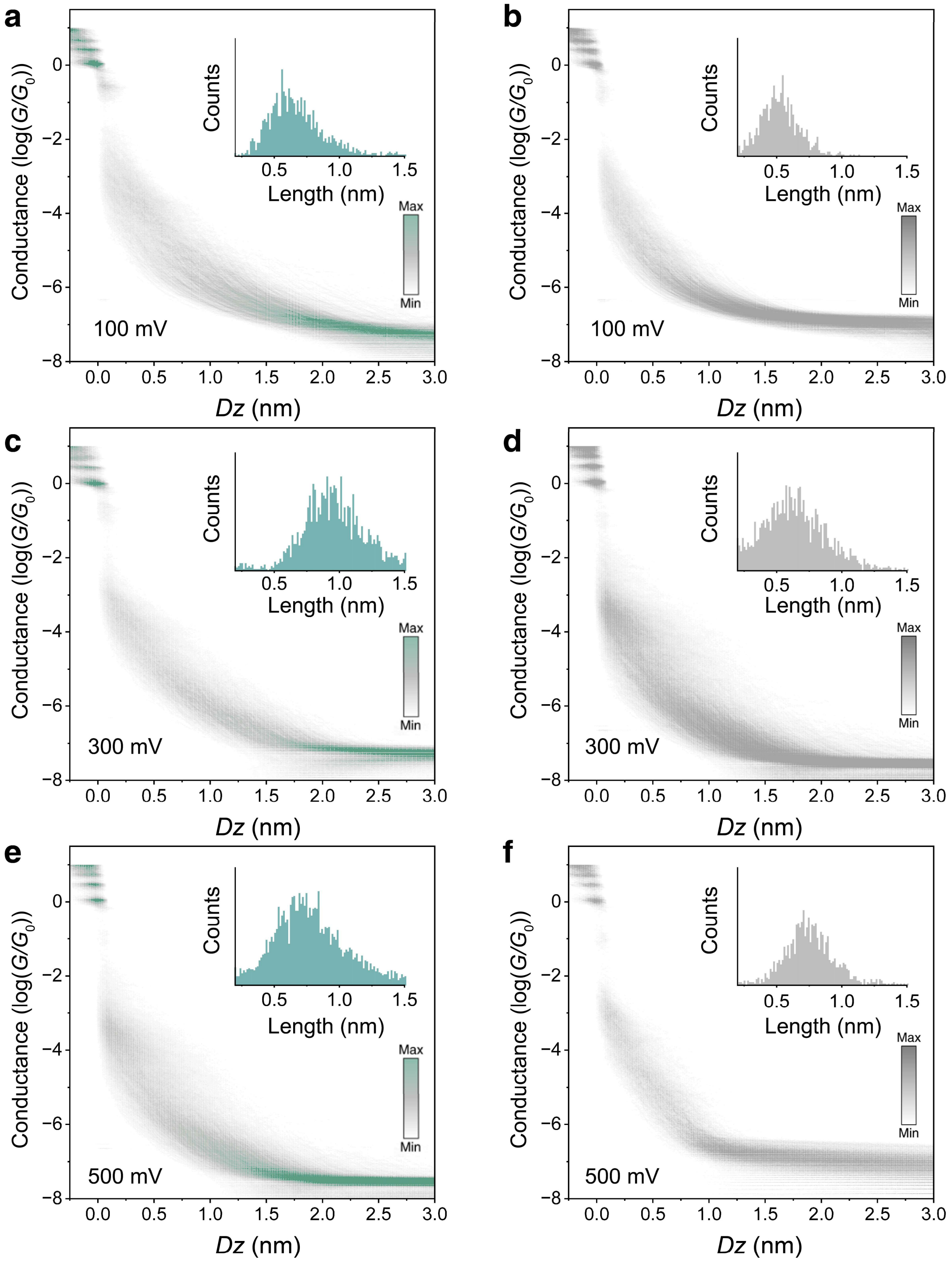

Supplementary Fig. 26 | 2D conductance histograms of DAS. 2D conductance histograms of **a, c, e** *linear* DAS and **b, d, f** *cyclic* DAS at bias voltages of 100 mV, 300 mV and 500 mV, respectively. The color bar indicates the number of counts. Insets show the relative stretching distance distributions of *linear* and *cyclic* DAS junctions.

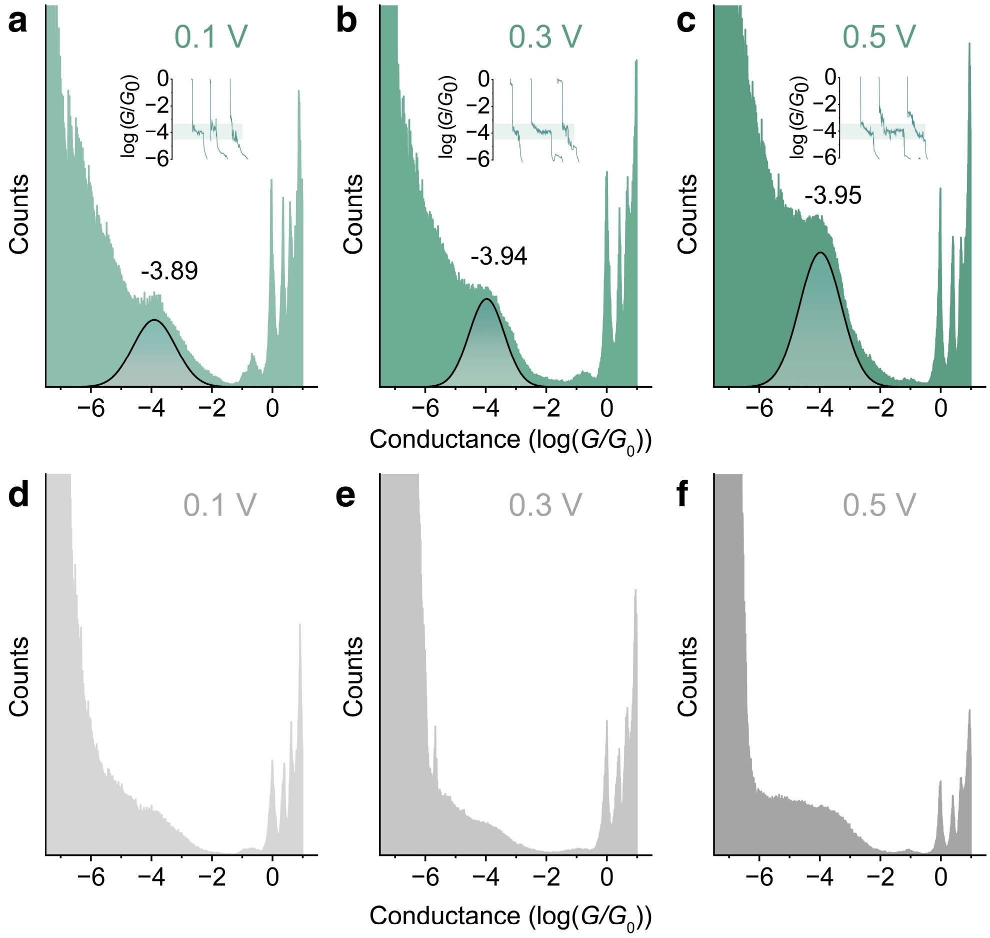

Supplementary Fig. 27 | 1D logarithmic conductance histograms of DAS. a-c 1D logarithmic conductance histograms for *linear* DAS at bias voltages of 100 mV, 300 mV and 500 mV, respectively. **d-f** 1D logarithmic conductance histograms for *cyclic* DAS at bias voltages of 100 mV, 300 mV and 500 mV, respectively. Inset shows the typical individual conductance traces.

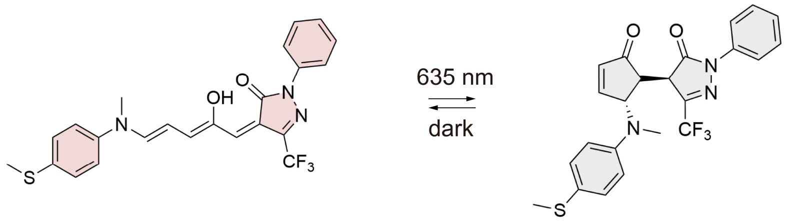

Supplementary Fig. 28 | Chemical structure of SDA. Chemical structure of SDA in its *linear* state (left) and *cyclic* state (right) upon 635 nm light irradiation and in the dark.

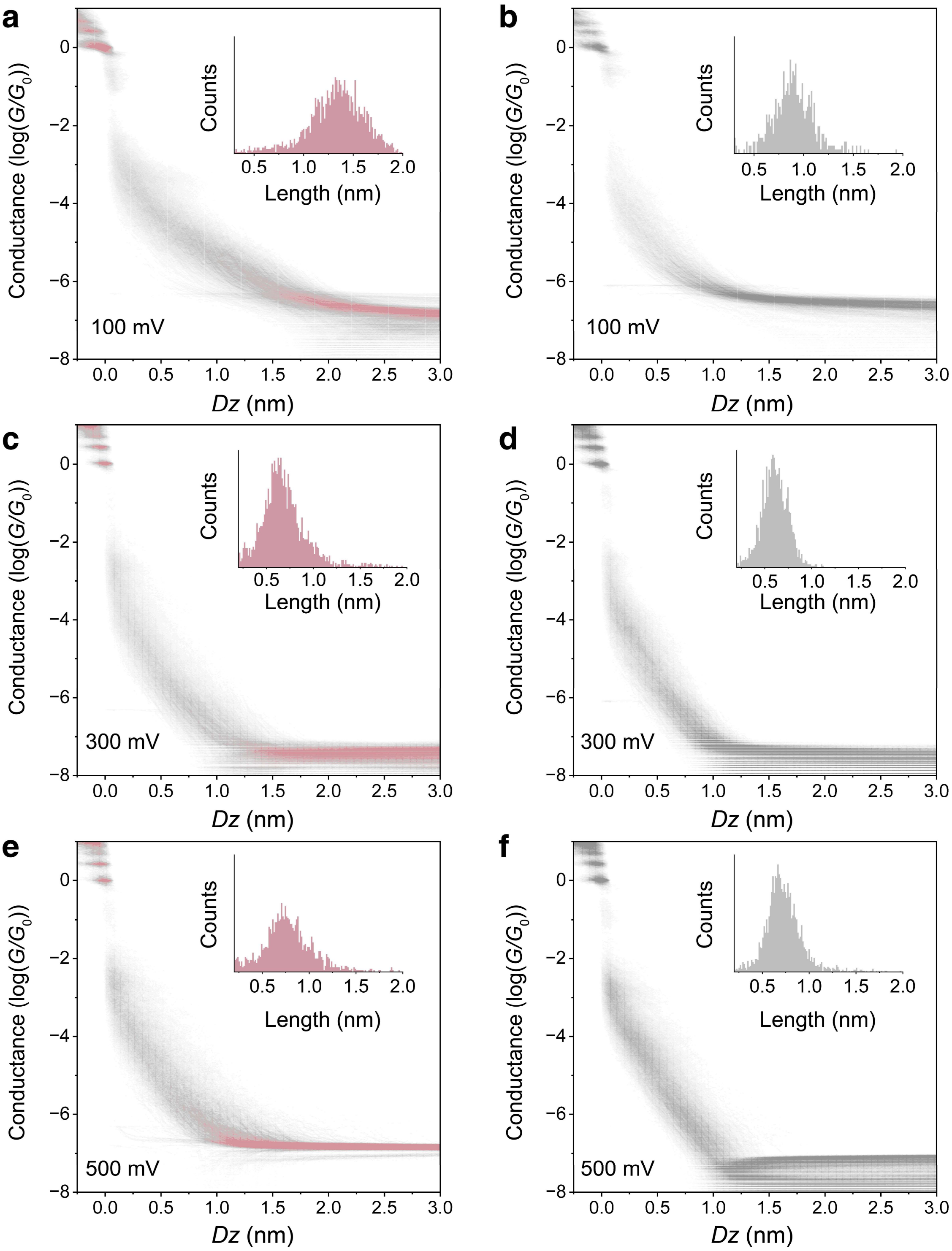

Supplementary Fig. 29 | 2D conductance histograms of SDA. 2D conductance histograms of **a, c, e** *linear* SDA and **b, d, f** *cyclic* SDA at bias voltages of 100 mV, 300 mV and 500 mV, respectively. The color bar indicates the number of counts. Insets show the relative stretching distance distributions of *linear* and *cyclic* SDA junctions.

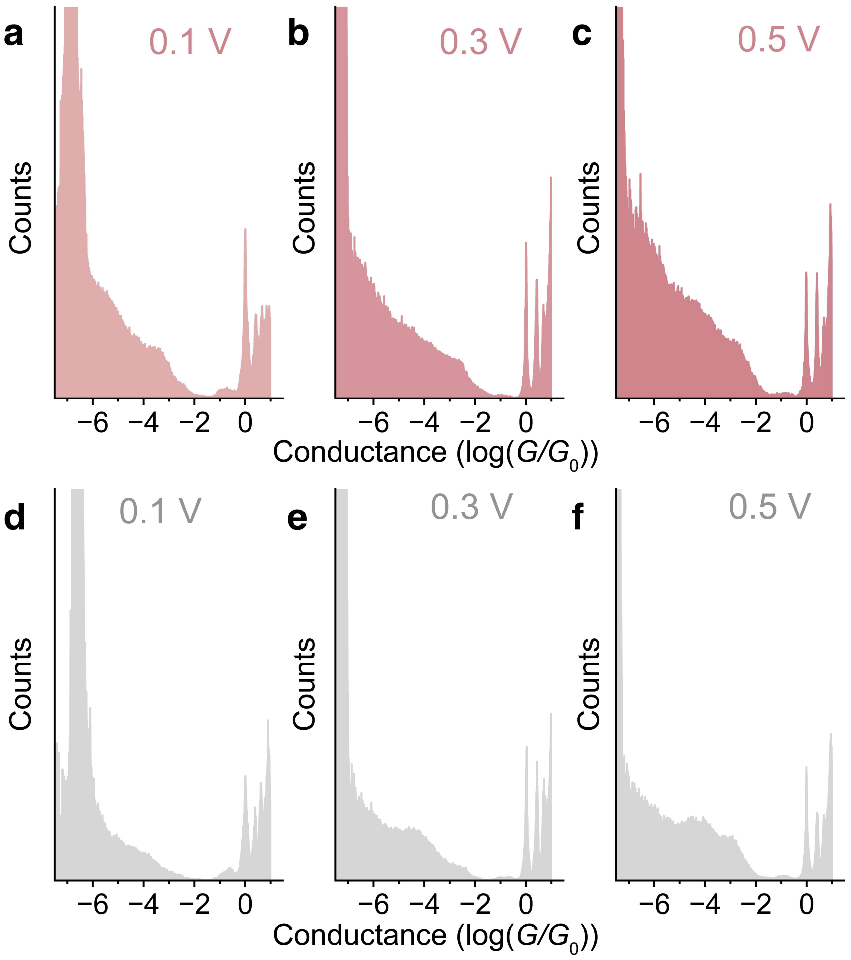

Supplementary Fig. 30 | 1D logarithmic conductance histograms of SDA. a-c 1D logarithmic conductance histograms for *linear* SDA at bias voltages of 100 mV, 300 mV and 500 mV, respectively. **d-f** 1D logarithmic conductance histograms for *cyclic* SDA at bias voltages of 100 mV, 300 mV and 500 mV, respectively.

4.3 SSDA and SDAS (two anchoring sites)

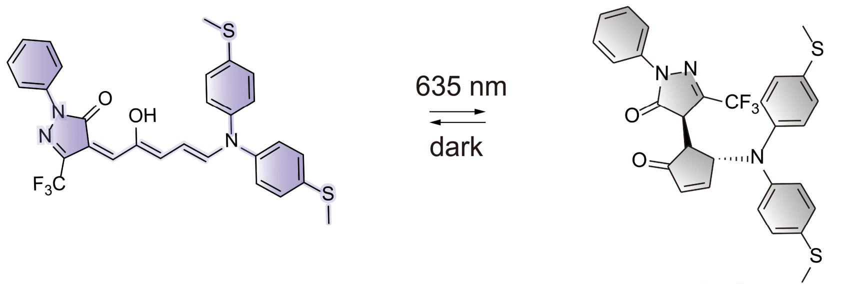

Supplementary Fig. 31 | Chemical structure of SSDA. Chemical structure of SSDA in its *linear* state (left) and *cyclic* state (right) upon 635 nm light irradiation and in the dark.

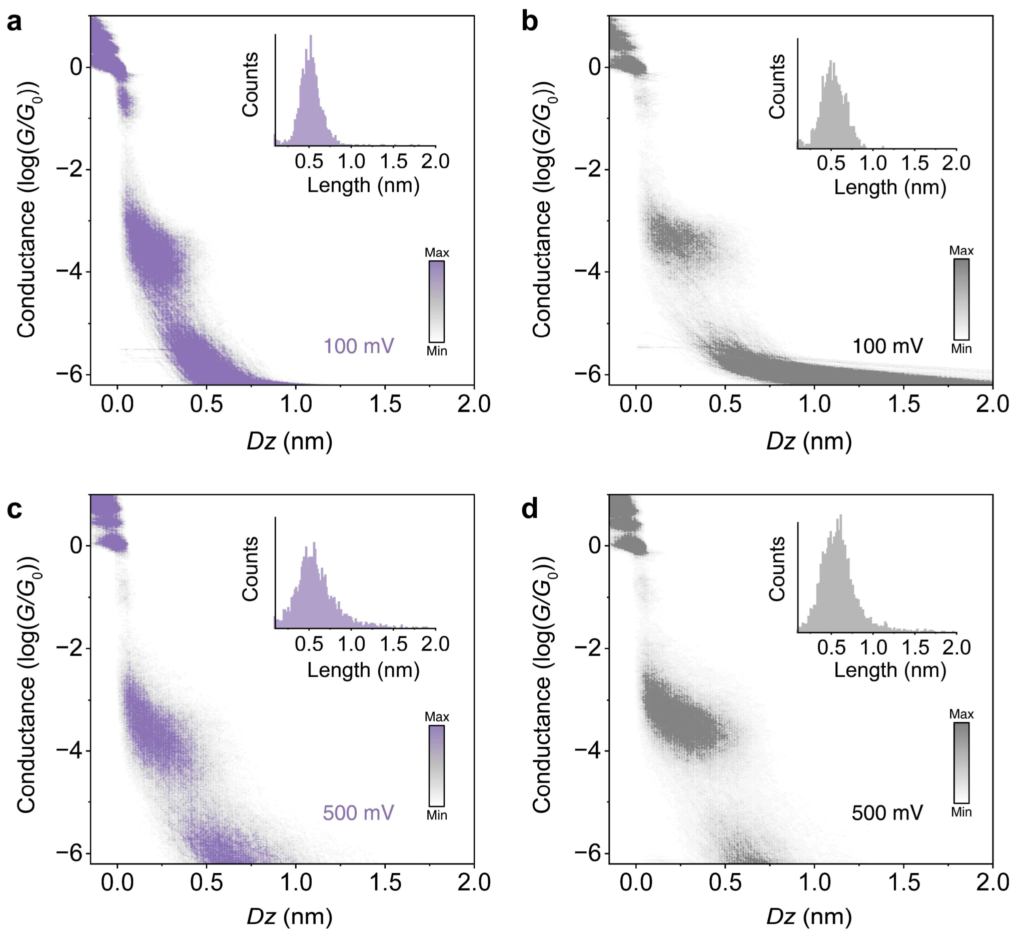

Supplementary Fig. 32 | 2D conductance histograms of SSDA. 2D conductance histograms of **a, c** *linear* SSDA and **b, d** *cyclic* SSDA at bias voltages of 100 mV and 500 mV, respectively. The color bar indicates the number of counts. Insets show the relative stretching distance distributions of *linear* and *cyclic* SSDA junctions.

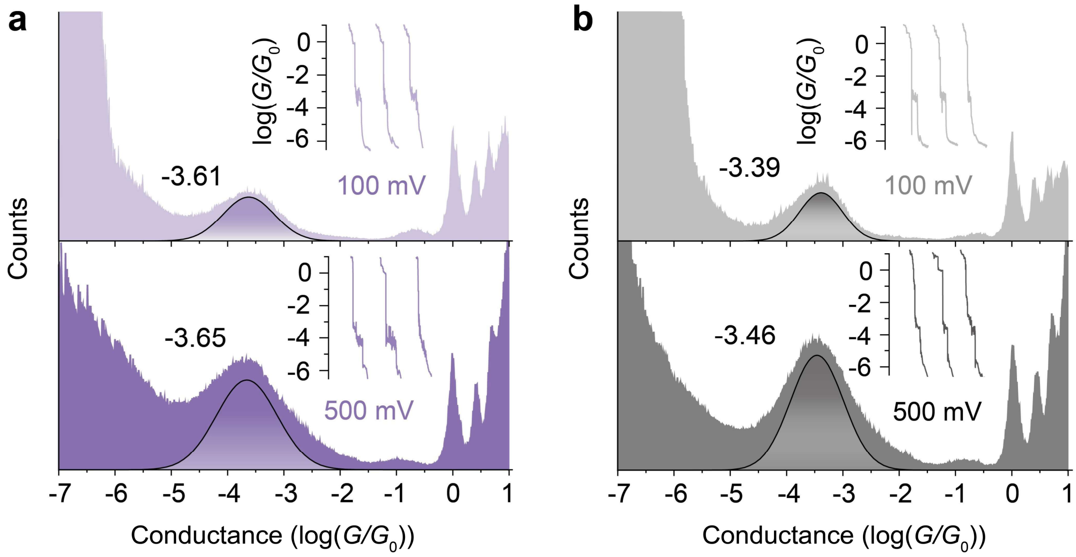

Supplementary Fig. 33 | 1D logarithmic conductance histograms of SSDA. a One-dimensional logarithmic conductance histograms of *linear* SSDA at bias voltages of 100 mV and 500 mV. Insets show the typical individual conductance traces. **b** One-dimensional logarithmic conductance histograms of *cyclic* SSDA at bias voltages of 100 mV and 500 mV. Insets show the typical individual conductance traces.

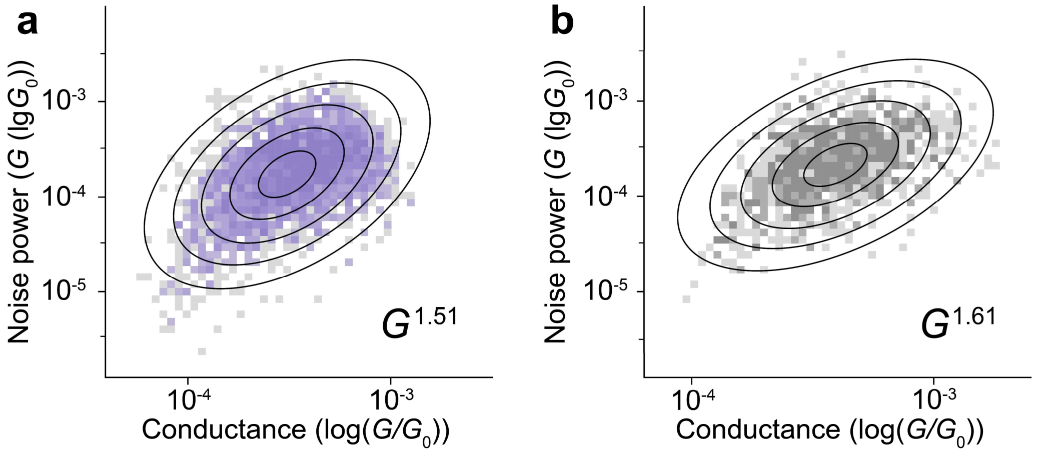

Supplementary Fig. 34 | Flicker noise analysis of SSDA. Flicker noise PSD of **a** *linear* SSDA and **b** *cyclic* SSDA.

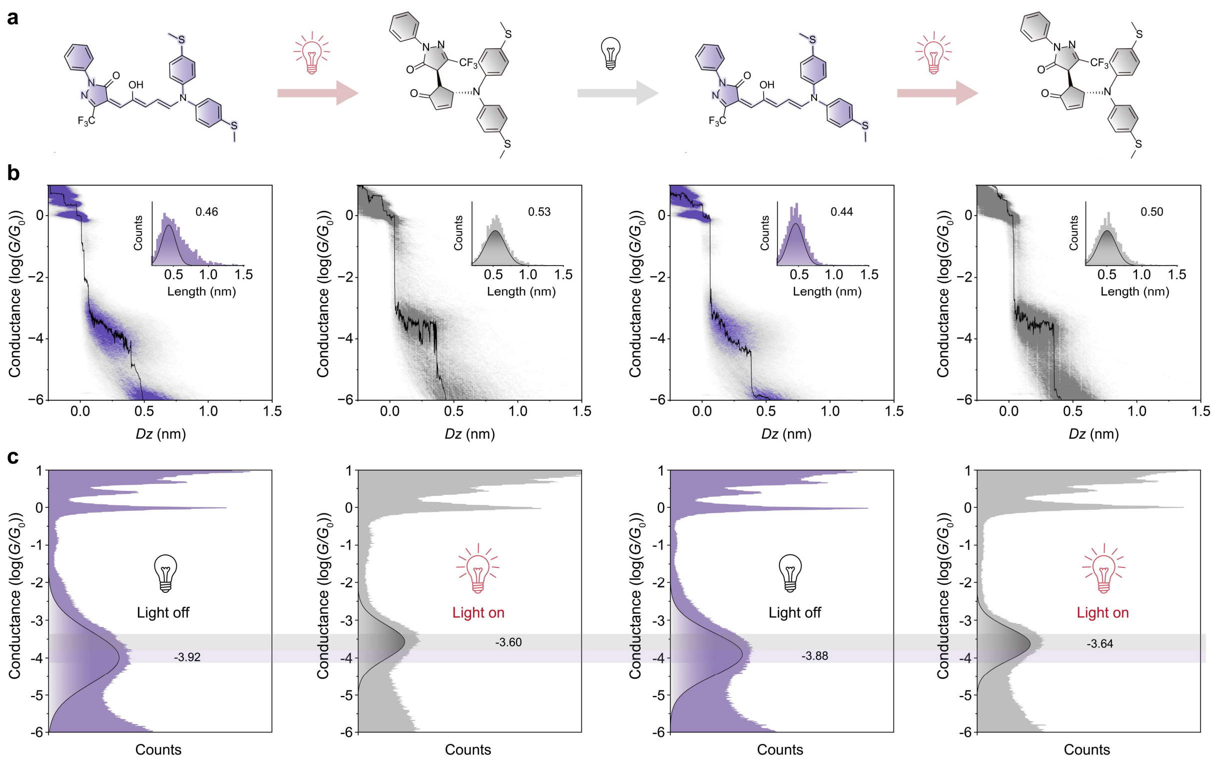

Supplementary Fig. 35 | Reversible conductance switching of SSDA at 300 mV bias. a Chemical structures and reversible *linear*-*cyclic* isomerization of SSDA. **b** Two-dimensional conductance-distance histograms of *linear* SSDA (purple) and *cyclic* SSDA (gray) junctions measured at a bias voltage of 300 mV; the color scale represents the number of counts. Insets show the relative stretching distance distributions for *linear* SSDA and *cyclic* SSDA. **c** One-dimensional logarithmic conductance histograms of *linear* (purple) and *cyclic* (gray) SSDA recorded under "light off" and "light on" conditions at 300 mV bias.

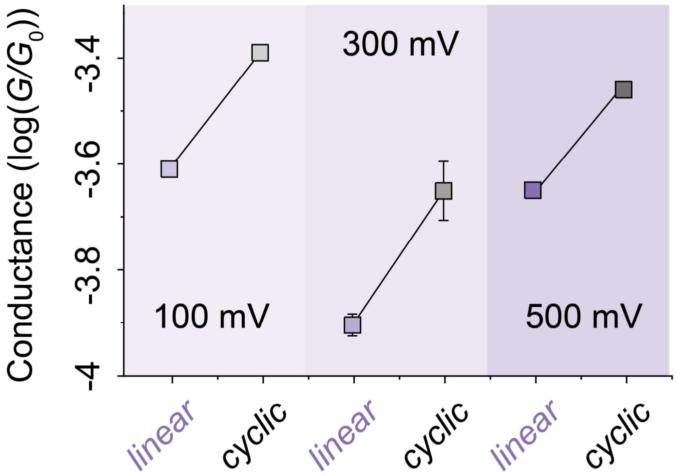

Supplementary Fig. 36 | Summary of conductance for *linear* and *cyclic* SSDA. Conductance for *linear* and *cyclic* SSDA at bias voltages of 100 mV, 300 mV and 500 mV.

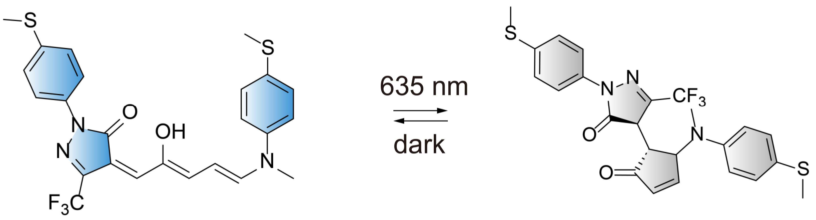

Supplementary Fig. 37 | Chemical structure of SDAS. Chemical structure of SDAS in its *linear* state (left) and *cyclic* state (right) upon 635 nm light irradiation and in the dark.

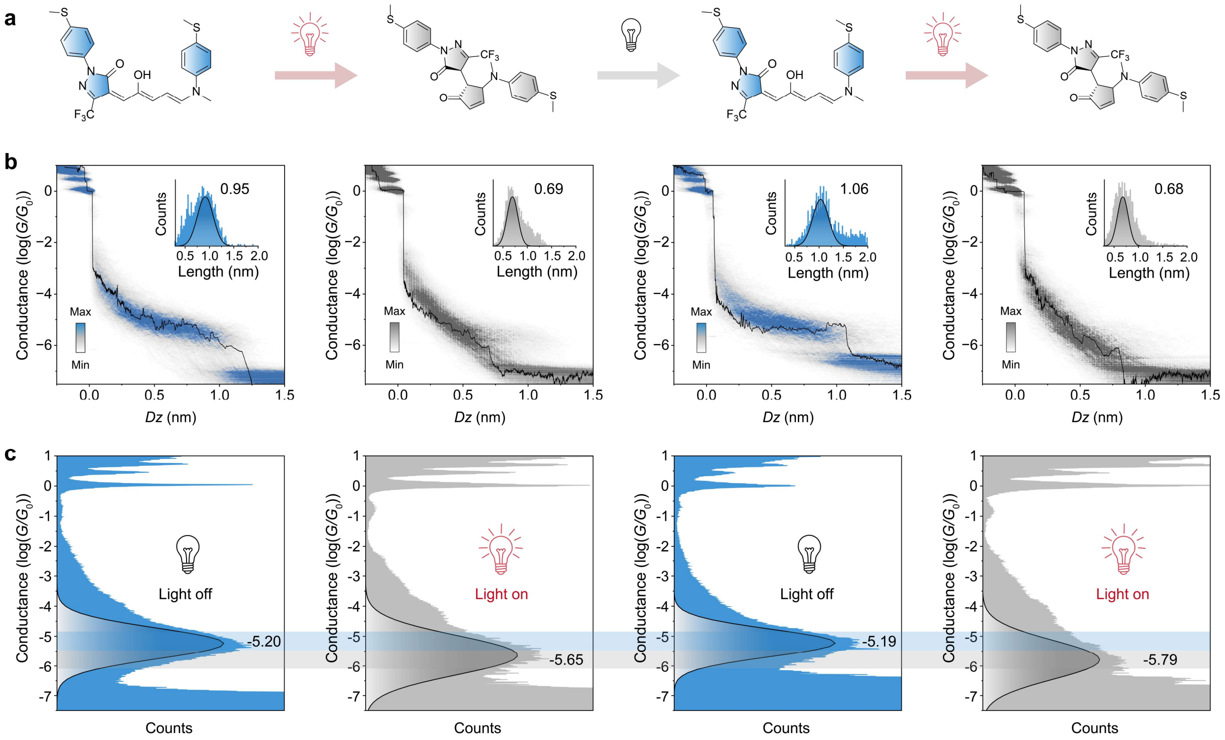

Supplementary Fig. 38 | Reversible conductance switching of SDAS at 300 mV bias. a Chemical structures and reversible *linear*-*cyclic* isomerization of SDAS. **b** Two-dimensional conductance-distance histograms of *linear* SDAS (blue) and *cyclic* SDAS (gray) junctions measured at a bias voltage of 300 mV; the color scale represents the number of counts. Insets show the relative stretching distance distributions for *linear* SDAS and *cyclic* SDAS. **c** One-dimensional logarithmic conductance histograms of *linear* (blue) and *cyclic* (gray) SDAS recorded under “light off” and “light on” conditions at 300 mV bias.

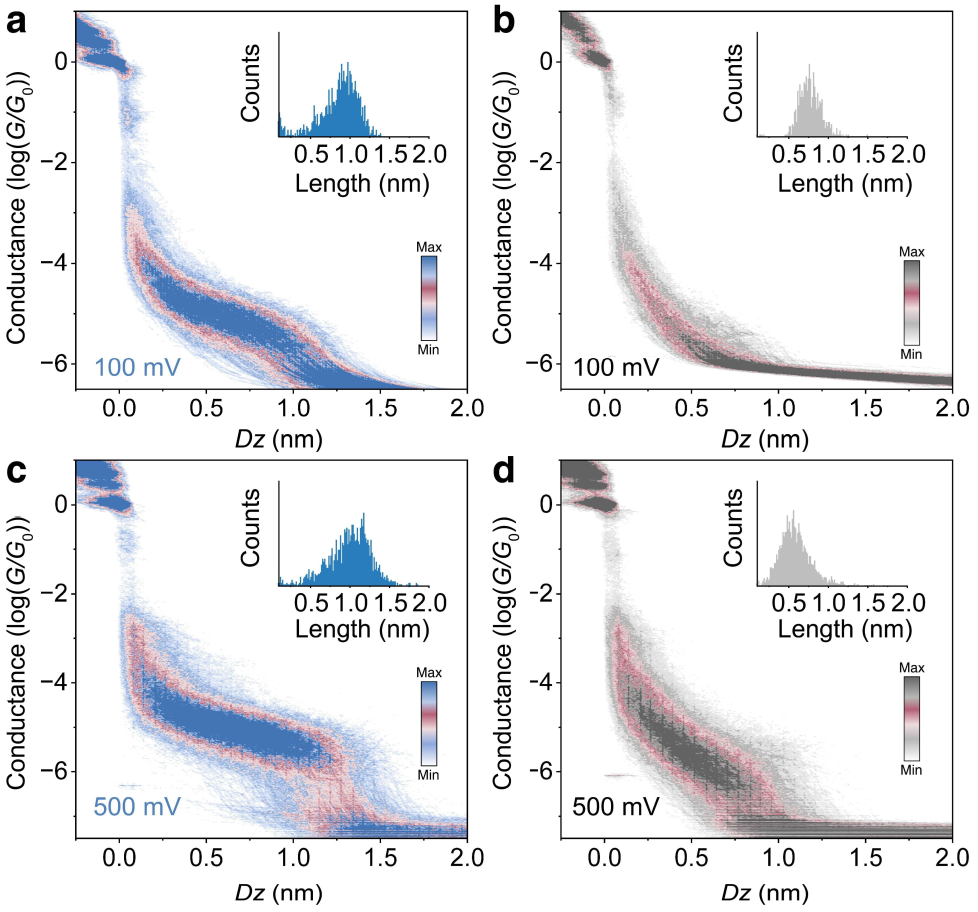

Supplementary Fig. 39 | 2D conductance histograms of SDAS. 2D conductance histograms of **a, c** *linear* SDAS and **b, d** *cyclic* SDAS at bias voltages of 100 mV and 500 mV, respectively. The color bar indicates the number of counts. Insets show the relative stretching distance distributions of *linear* and *cyclic* SDAS junctions.

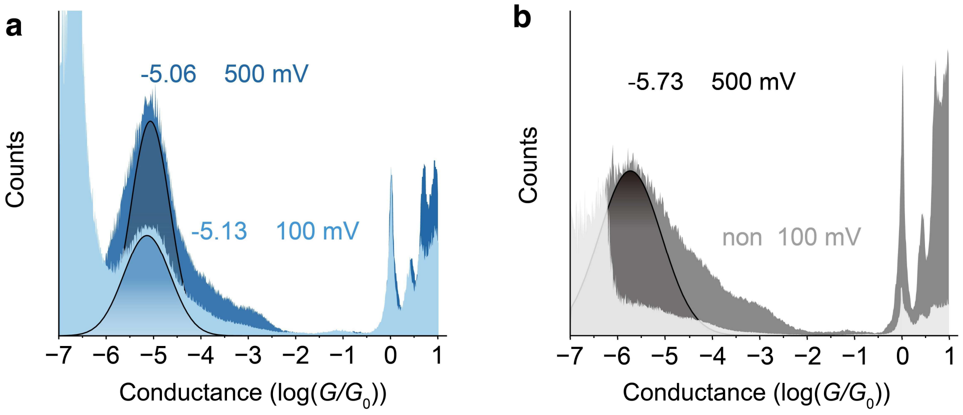

Supplementary Fig. 40 | 1D logarithmic conductance histograms of SDAS. a One-dimensional logarithmic conductance histograms of *linear* SDAS at bias voltages of 100 mV and 500 mV. **b** One-dimensional logarithmic conductance histograms of *cyclic* SDAS at bias voltages of 100 mV and 500 mV.

4.4 SSDAS (three anchoring sites)

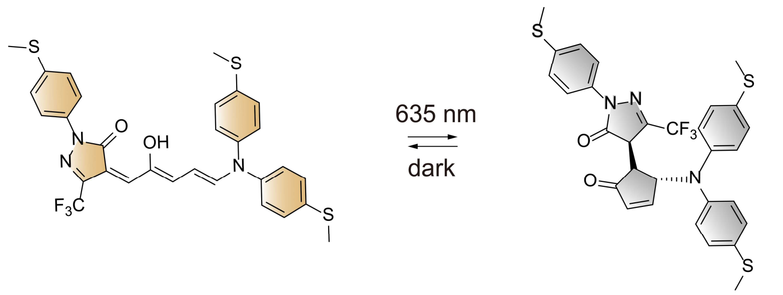

Supplementary Fig. 41 | Chemical structure of SSDAS. Chemical structure of SSDAS in its *linear* state (left) and *cyclic* state (right) upon 635 nm light irradiation and in the dark

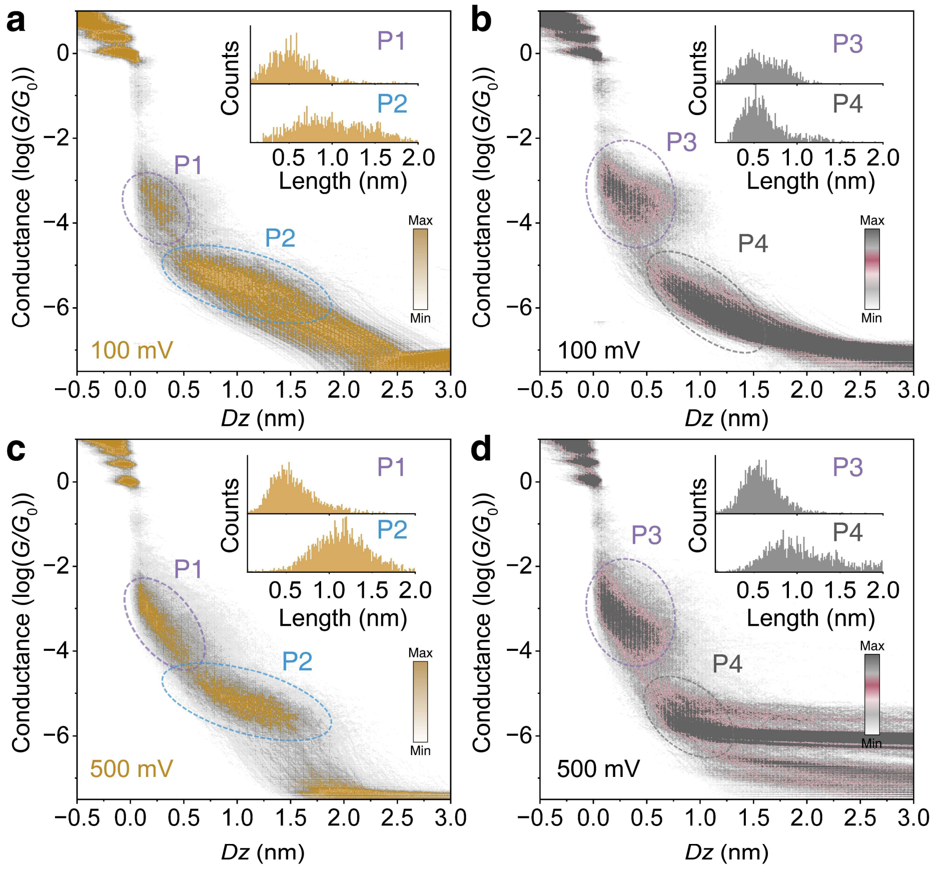

Supplementary Fig. 42 | 2D conductance histograms of SSDAS. 2D conductance histograms of **a, c** *linear* SSDAS and **b, d** *cyclic* SSDAS at bias voltages of 100 mV and 500 mV, respectively. The color bar indicates the number of counts. Insets show the relative stretching distance distributions of *linear* and *cyclic* SSDAS junctions.

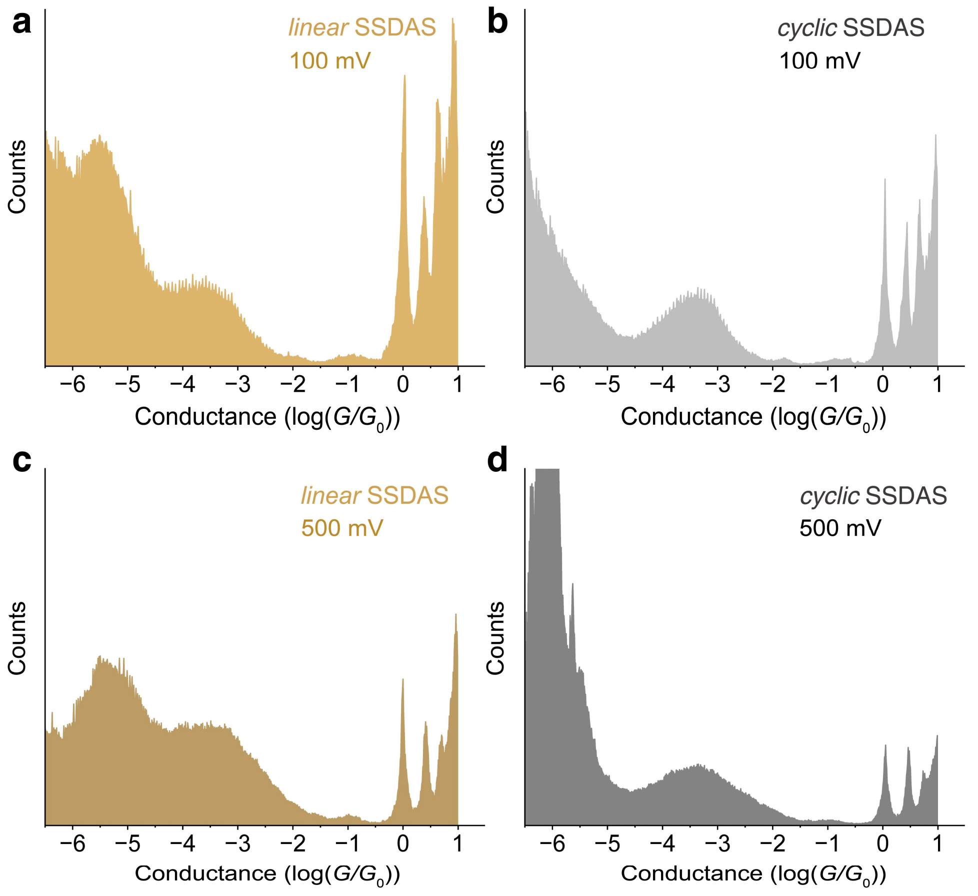

Supplementary Fig. 43 | 1D logarithmic conductance histograms of SSDAS. 1D logarithmic conductance histograms of **a, c** *linear* SSDAS and **b, d** *cyclic* SSDAS at bias voltages of 100 mV and 500 mV, respectively.

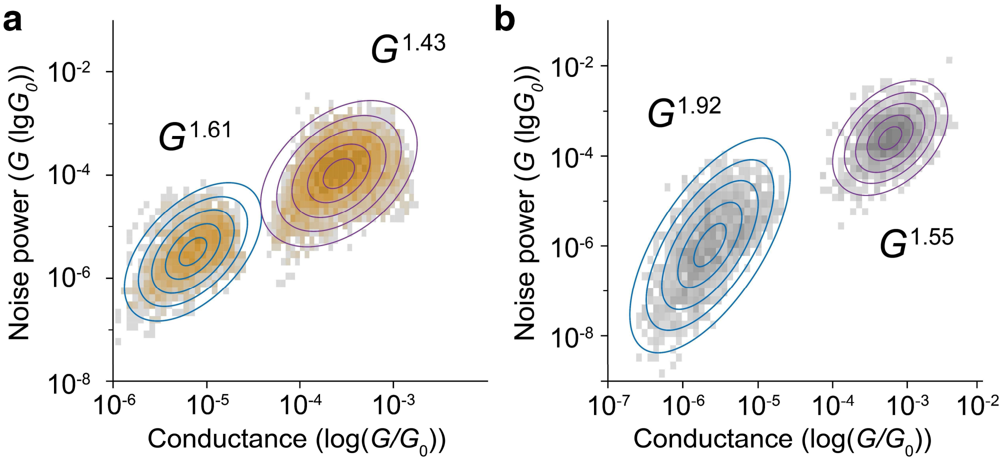

Supplementary Fig. 44 | Flicker noise analysis of SSDA. Flicker noise PSD of **a** *linear* SSDAS and **b** *cyclic* SSDAS.

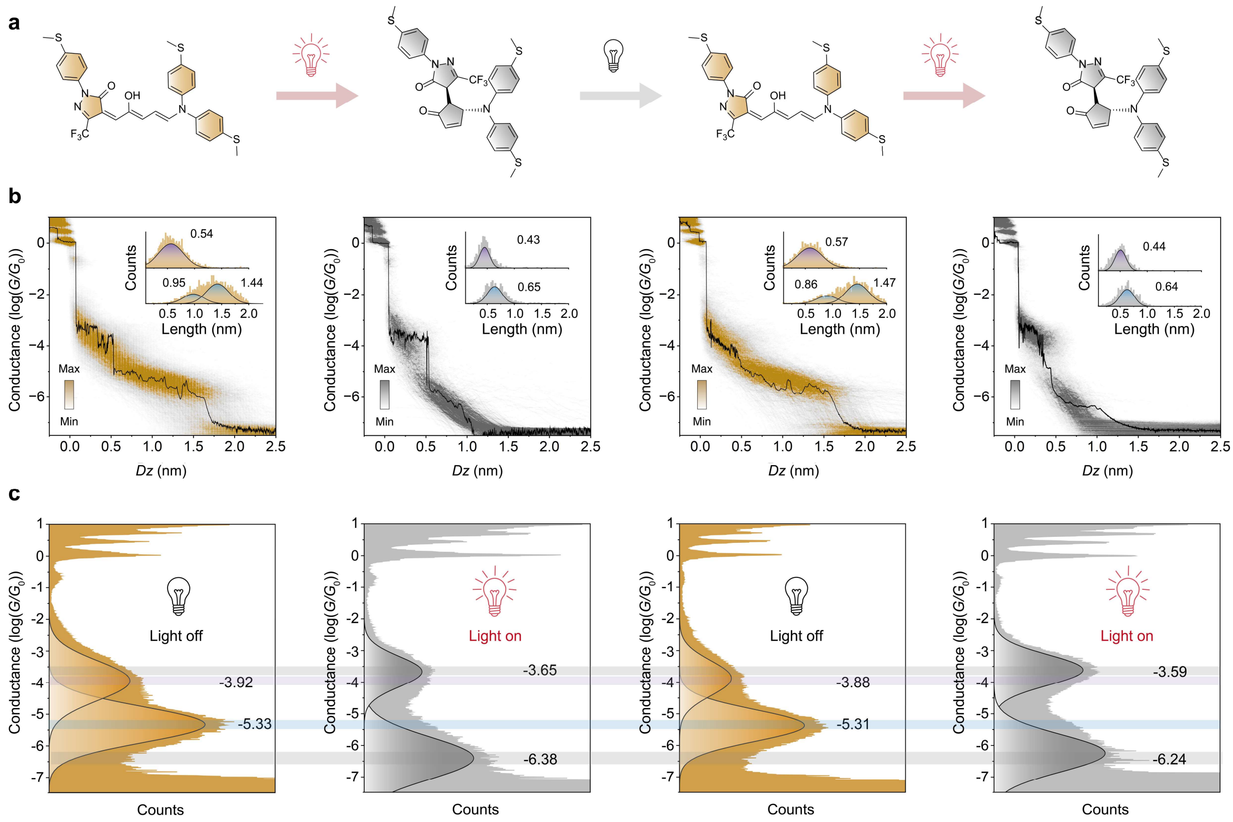

Supplementary Fig. 45 | Reversible conductance switching of SSDAS at 300 mV bias. a Chemical structures and reversible *linear-cyclic* isomerization of SSDAS. **b** Two-dimensional conductance-distance histograms of *linear* SSDAS (yellow) and *cyclic* SSDAS (gray) junctions measured at a bias voltage of 300 mV; the color scale represents the number of counts. Insets show the relative stretching distance distributions for *linear* SSDAS and *cyclic* SSDAS. **c** One-dimensional logarithmic conductance histograms of *linear* (yellow) and *cyclic* (gray) SSDAS recorded under "light off" and "light on" conditions at 300 mV bias.

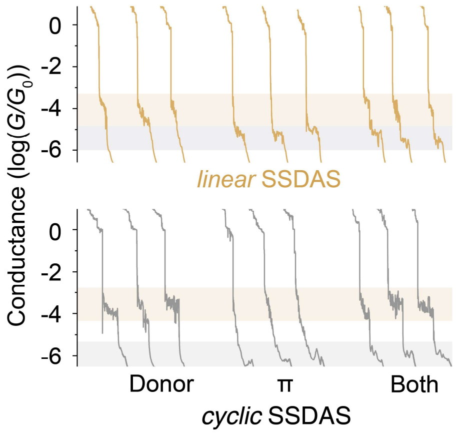

Supplementary Fig. 46 | The typical individual conductance traces for SSDAS. Typical traces for *linear* SSDAS (upper, yellow) and *cyclic* SSDAS (lower, gray) at bias voltage of 300 mV, conductive pathways through donor, π-bridge and both on single-molecule junctions are observed.

5 Electron transport calculations

The geometry optimization and transmission functions of the single-molecule device were calculated using GGA and PBE functional with the NEGF approach in Atomistix Tool Kit (ATK) package[14,15]. To construct the single-molecule device models, the molecule structures were firstly optimized by Gaussian 16. The optimized molecular structures were then placed between two gold electrodes to construct the single-molecule junction. The initial gold sulfur bond distance was set at 0.25 nm. The geometries of molecules and the gold clusters are optimized in a gas atmosphere with k-points sampling (1, 1, 150) with a force threshold of 0.05 eV/Å. The single-ζ polarized (SZP) basis set was used for Au atoms and the double-ζ polarized (DZP) basis set was used for other atoms, with a real space grid defined with an equivalent energy cut-off of 80 Hartree. The devices were then fabricated to calculate the transmission coefficient with the k-points of (7, 7). The transmission spectra dependent on the energy of molecular orbitals were obtained.

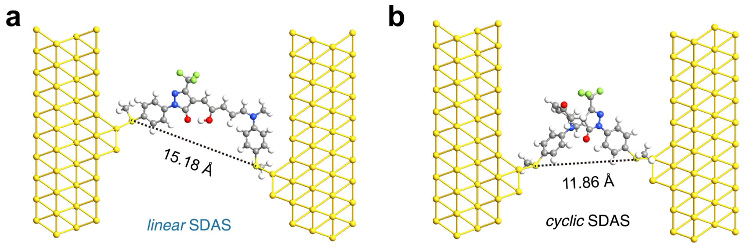

Supplementary Fig. 47 | The optimized molecular device structures. The optimized molecular device structures of **a** *linear* SDAS and **b** *cyclic* SDAS.

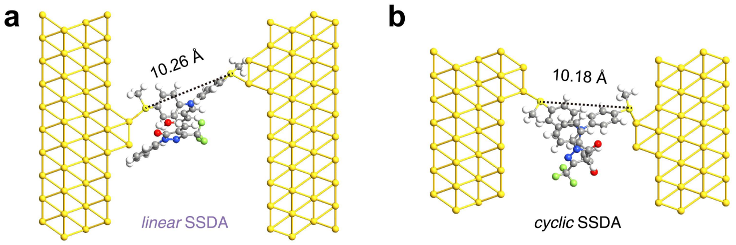

Supplementary Fig. 48 | The optimized molecular device structures. The optimized molecular device structures of **a** *linear* SSDA and **b** *cyclic* SSDA.

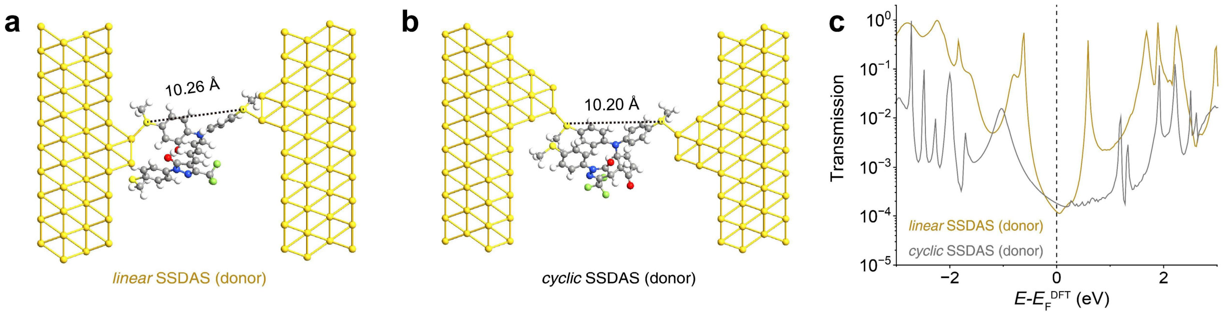

Supplementary Fig. 49 | The optimized molecular device structures and the transmission spectra. The optimized molecular device structures of **a** *linear* SSDAS (donor pathway) and **b** *cyclic* SSDAS (donor pathway). **c** The transmission spectra of *linear* (yellow) and *cyclic* (gray) SSDAS (donor pathway).

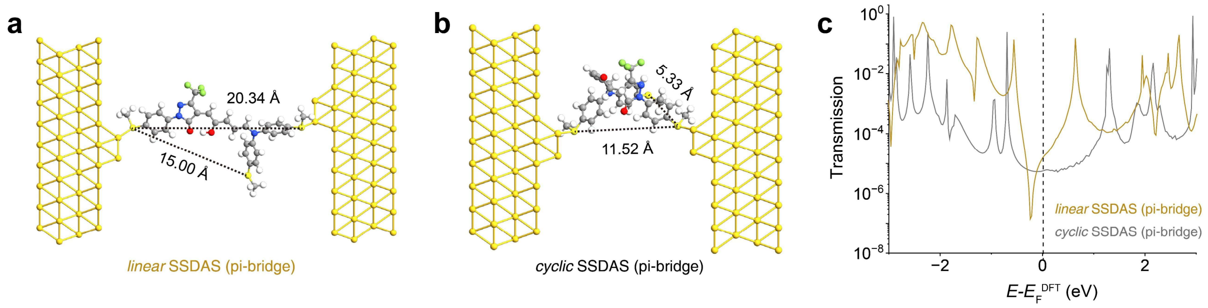

Supplementary Fig. 50 | The optimized molecular device structures and the transmission spectra. The optimized molecular device structures of **a** *linear* SSDAS (π-bridge pathway) and **b** *cyclic* SSDAS (π-bridge pathway). **c** The transmission spectra of *linear* (yellow) and *cyclic* (gray) SSDAS (π-bridge pathway).

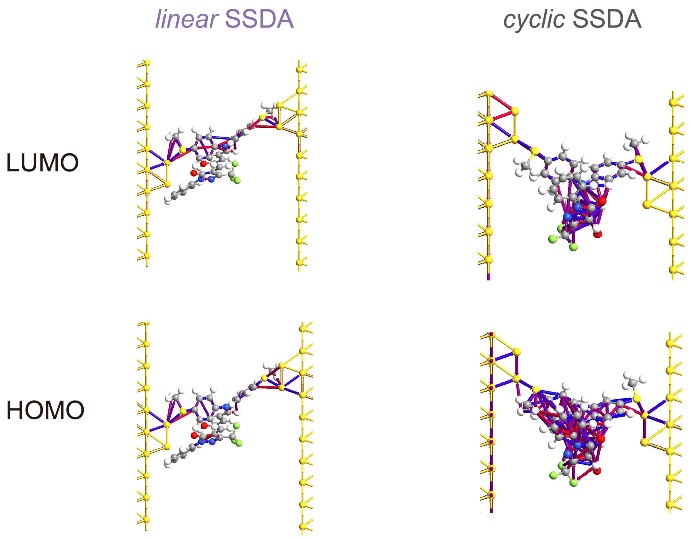

Supplementary Fig. 51 | The transmission pathways of molecular devices (donor pathway) at the Fermi energy for SSDA. The images display the transmission pathways for *cyclic* and *linear* SSDA in both HOMO and LUMO states under different conditions. The color of the arrows indicates the transport direction, and the size of the arrows is in proportion to the contribution of the transmission. Blue arrows represent positive channel, red arrows represent negative channel. The threshold value of pathway is uniformly selected as 0.05 for a better comparison.

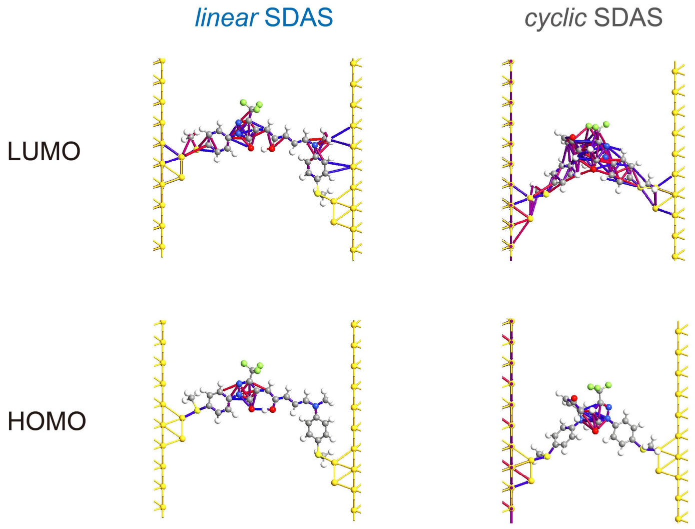

Supplementary Fig. 52 | The transmission pathways of molecular devices (π-bridge pathway) at the Fermi energy for SDAS. The images display the transmission pathways for *cyclic* and *linear* SDAS in both HOMO and LUMO states under different conditions. The color of the arrows indicates the transport direction, and the size of the arrows is in proportion to the contribution of the transmission. Blue arrows represent positive channel, red arrows represent negative channel. The threshold value of pathway is uniformly selected as 0.05 for a better comparison.

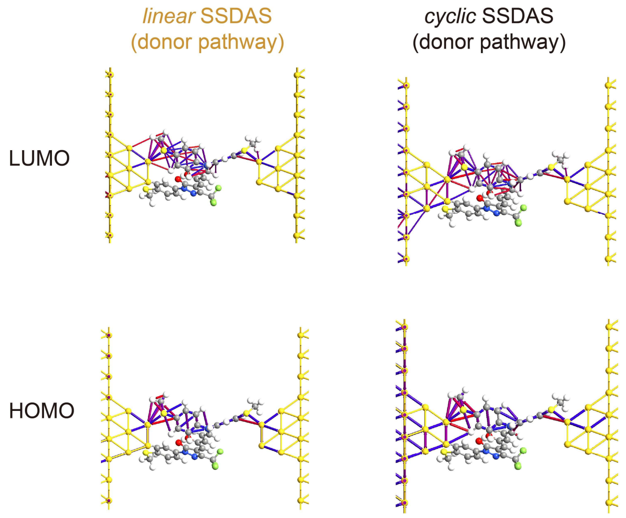

Supplementary Fig. 53 | The transmission pathways of molecular devices (donor pathway) at the Fermi energy for SSDAS. The images display the transmission pathways for the donor pathway in *cyclic* and *linear* SSDAS in both HOMO and LUMO states under different conditions. The color of the arrows indicates the transport direction, and the size of the arrows is in proportion to the contribution of the transmission. Blue arrows represent positive channel, red arrows represent negative channel. The threshold value of pathway is uniformly selected as 0.05 for a better comparison.

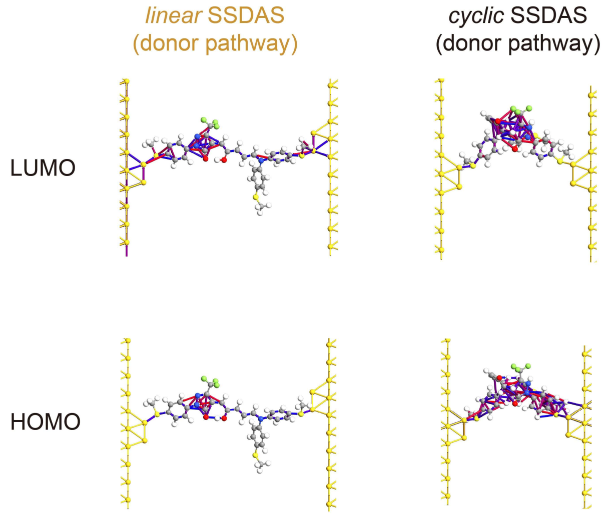

Supplementary Fig. 54 | The transmission pathways of molecular devices (π-bridge pathway) at the Fermi energy for SSDAS. The images display the transmission pathways for π-bridge pathway in *cyclic* and *linear* SSDAS in both HOMO and LUMO states under different conditions. The color of the arrows indicates the transport direction, and the size of the arrows is in proportion to the contribution of the transmission. Blue arrows represent positive channel, red arrows represent negative channel. The threshold value of pathway is uniformly selected as 0.05 for a better comparison.

6 The isomerization characteristics

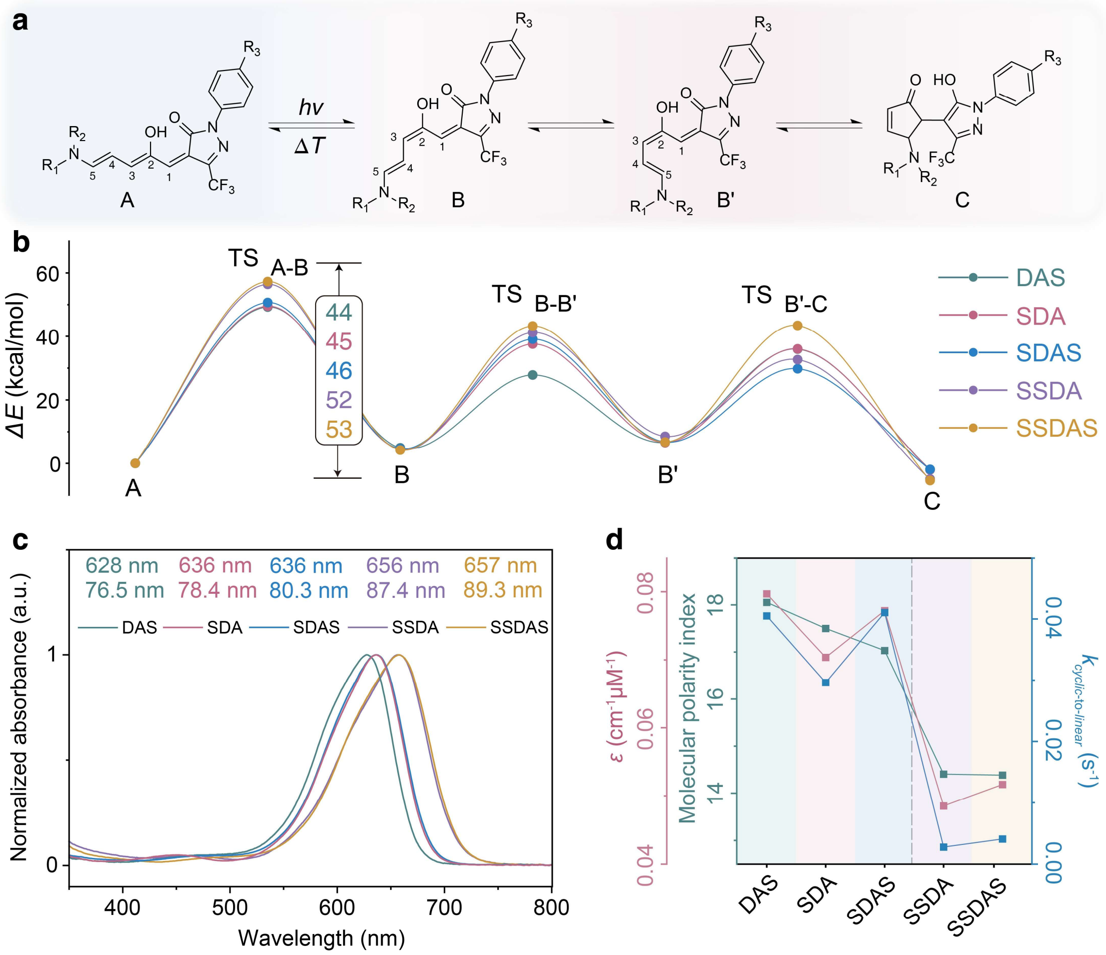

Supplementary Fig. 55 | The spectral and photoisomerization property of DASAs. a Schematic illustration of typical isomerization process between *linear* and *cyclic* DASAs. **b** Calculated energy landscape for the DASAs through the *linear*-to-*cyclic* isomerization based on M06-2X/6-311G(d) (Grimme's D3 dispersion model using the SMD solvent model in TCB (Eps=2.25)). **c** Normalized absorption spectra of DAS, SDA, SDAS, SSDA, and SSDAS in TCB. The corresponding maximum absorption wavelengths and full width at half maximum (FWHM) values are labelled above each curve. **d** Calculated molecular polarity index (*MPI*, green axis on the left), molar absorption coefficient (red axis on the left) and *cyclic*-to-*linear* isomerization rate constants for the DASAs (blue axis on the right).

The photoisomerization of DASAs was studied before integrating into STM-BJ. Due to the push-pull nature, all the DASAs exhibit strong absorption bands in the visible light region beyond 620 nm (Supplementary Fig. 55c). The high molar absorption coefficients (~0.08 $cm^{-1}\mu m^{-1}$) indicates excellent color-developing ability of DASAs, suggesting their potential applications in

the dye industry (Supplementary Figs. 15-23). Under irradiation of 635 nm red light, the *linear*-to-*cyclic* isomerization of DASAs dramatically drops the absorption bands, which induces a colored-to-colorless transition and is generally recognized as a typical characteristic of negative photochromism (Supplementary Figs. 15-22). Typical *linear*-to-*cyclic* isomerization of DASAs could be briefly resolved into three transition steps (Supplementary Fig. 55a): (1) A-B transition is a light-dominated process that includes the C2-C3 inversion, the energy barrier for the transition state (TS) determines the thermodynamical favorability of this step; (2) B-B` transition with the C3-C4 rotation is generally regarded as a spontaneous process, while B` is a relatively stable intermediate which could be observed during the isomerization; (3) B`-C transition includes intramolecular proton transfer is critical for the generation of *cyclic* isomer, it should be notably the intermediate C with the enolate structure is unstable and spontaneously switches to *cyclic* via tautomerization.

Interestingly, the introduced thiomethylic groups significantly affect the isomerization behavior of DASAs by altering the absorption spectra and molecular energy during the isomerization process. The computed energy landscapes show that the energy barrier for the C2-C3 inversion (TS of A-B transition) is significantly increased after introducing two thiomethylic groups on the donor (SSDA and SSDAS), which sharply increases the time required for the backward *cyclic*-to-*linear* isomerization by one order of magnitude from tens of seconds to hundreds of seconds (Supplementary Fig. 55b and 55d, Supplementary Figs. 15-23). These indicate the thermodynamics and kinetics of the isomerization between *linear* and *cyclic* DASAs could be modulated by controlling the substituted groups on the electron donor and acceptor. Moreover, SSDA and SSDAS exhibit markedly red shifted and broadened absorption spectra compared with DASAs containing a single thiomethyl anchoring site (DAS and SDA) and with SDAS (Supplementary Fig. 55c). The absorption maxima progressively shift from 628 nm (DAS) to 657 nm (SSDAS), while the full width at half maximum (FWHM) increases from 78.4 nm to 89.3 nm. This trend suggests enhanced overlap of π-π^* and n-π^* transitions with stronger push-pull effects. The red-shift and spectral broadening thus directly reflect the electronic structural modifications introduced by thiomethylic group substitution.

Notably, it was found that a consistent trend emerges when comparing the spectral shifts and broadening, *cyclic*-to-*linear* isomerization kinetics, and molar extinction coefficients of DASAs, where a clear boundary was noticed between the DASAs with two thiomethylic groups on the donor (SSDA and SSDAS) and the others (Supplementary Fig. 55c and 55e, Supplementary Fig. 14). To understand the interrelationships, the molecular polarity indices (*MPI*) for the *linear* isomers are calculated, which demonstrates the lowest molecular polarity for SSDA and SSDAS (Supplementary Fig. 55e). Therefore, while the molecular structure is systematically modified, the electronic distribution on the π-bridge might be the essential factor governing the spectral property and isomerization behavior of DASAs. These findings indicate that the rational tuning of DASAs structure can modulate both spectral response and isomerization performance, offering a predictive framework for the design of photoresponsive molecules.

7 NMR and MS spectra

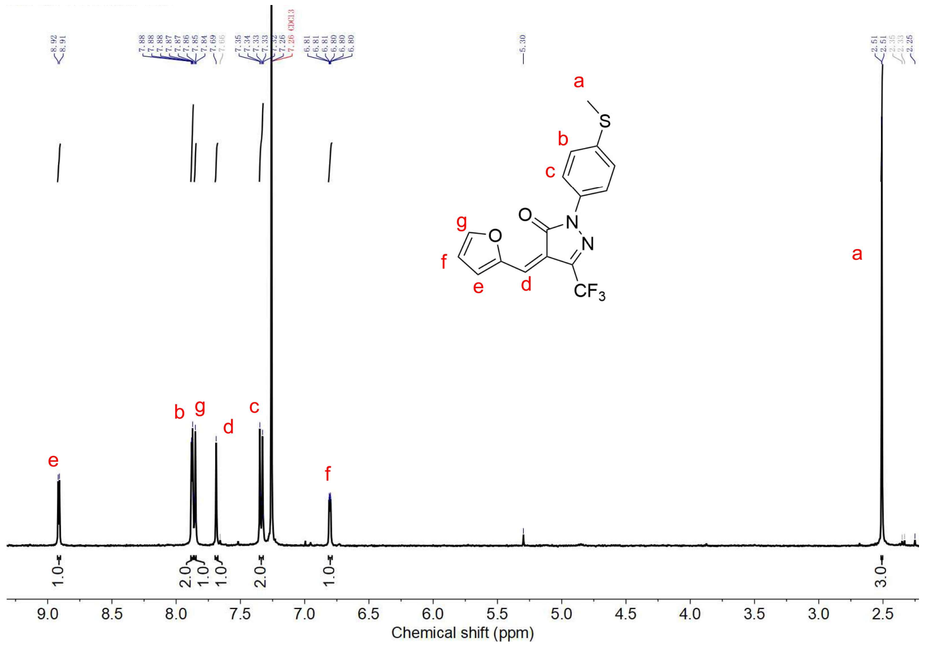

Supplementary Fig. 56 | ^{1}H NMR spectrum of 3 (400 MHz, 298 K), [3] = 10 mM, solvent: $CDCl_3$.

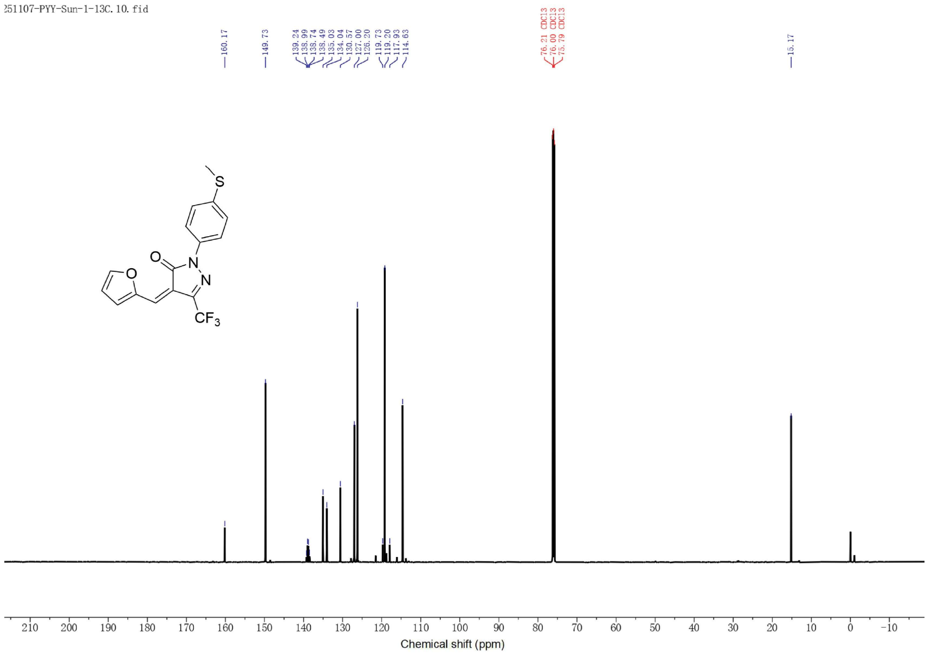

Supplementary Fig. 57 | ^{13}C NMR spectrum of 3 (100 MHz, 298 K), [3] = 10 mM, solvent: $CDCl_3$.

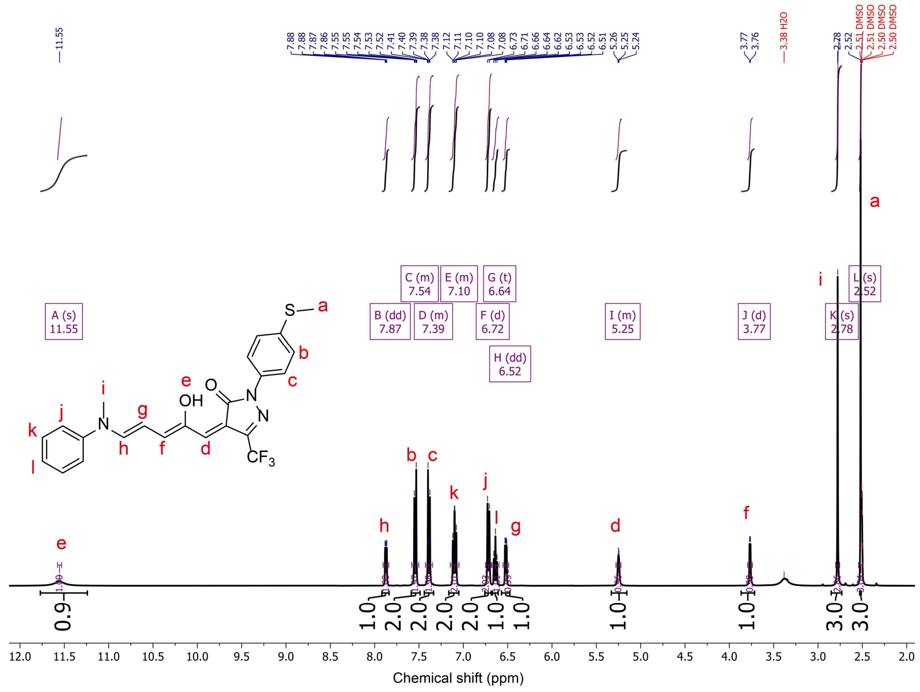

Supplementary Fig. 58 | ^{1}H NMR spectrum of DAS (400 MHz, 298 K), [DAS] = 10 mM, solvent: DMSO-*d*6.

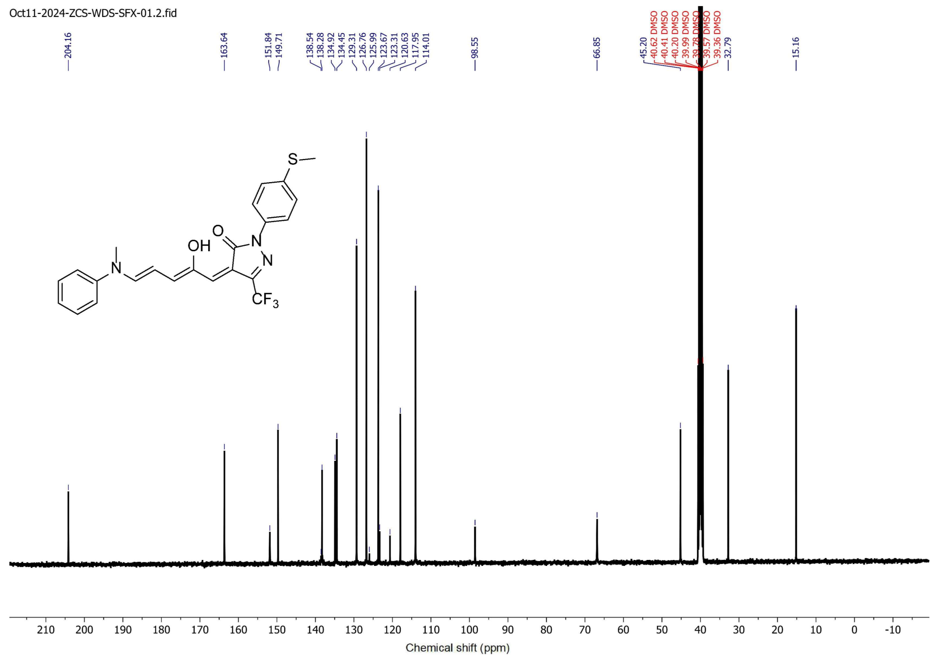

Supplementary Fig. 59 | ^{13}C NMR spectrum of DAS (100 MHz, 298 K), [DAS] = 10 mM, solvent: DMSO-*d*6.

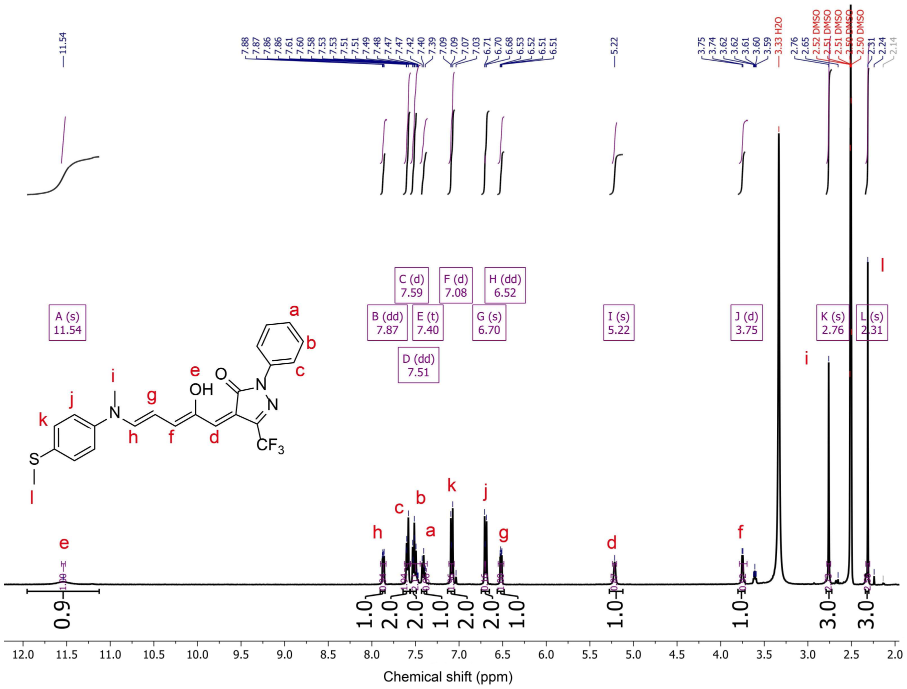

Supplementary Fig. 60 | ^{1}H NMR spectrum of SDA (400 MHz, 298 K), [SDA] = 10 mM, solvent: DMSO-*d*6.

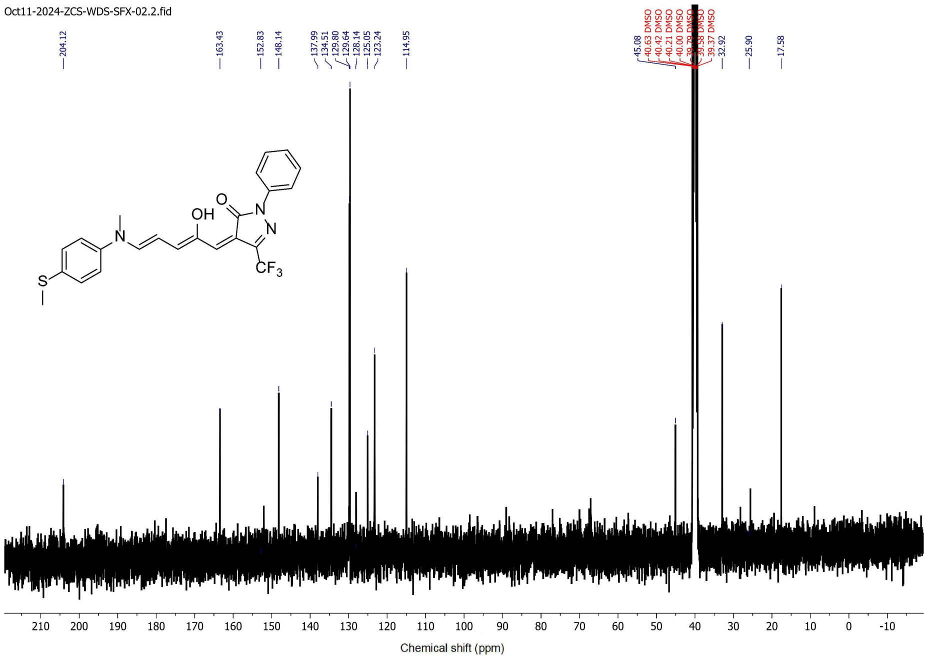

Supplementary Fig. 61 | ^{13}C NMR spectrum of SDA (100 MHz, 298 K), [SDA] = 10 mM, solvent: DMSO-*d*6.

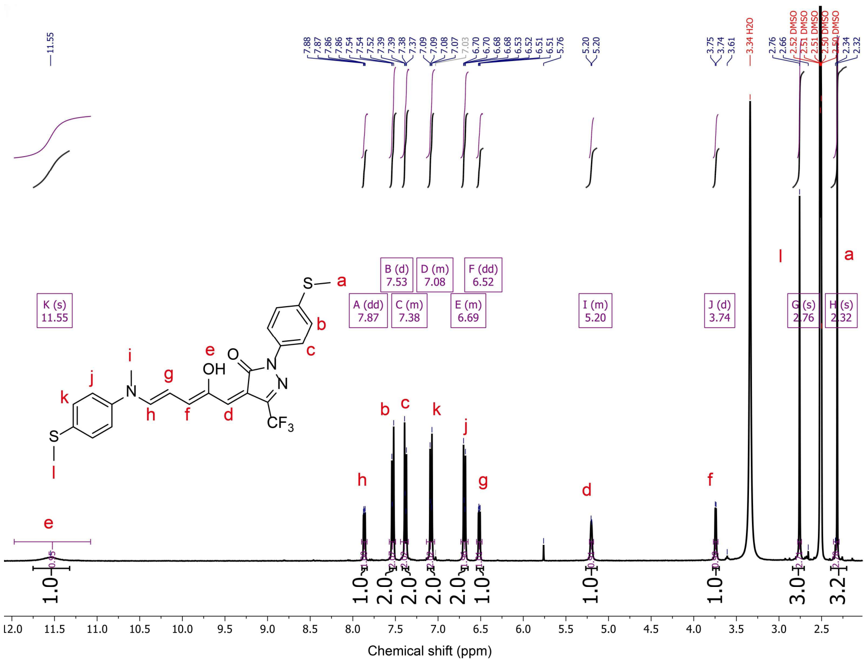

Supplementary Fig. 62 | ^{1}H NMR spectrum of SDAS (400 MHz, 298 K), [SDAS] = 10 mM, solvent: DMSO-*d*6.

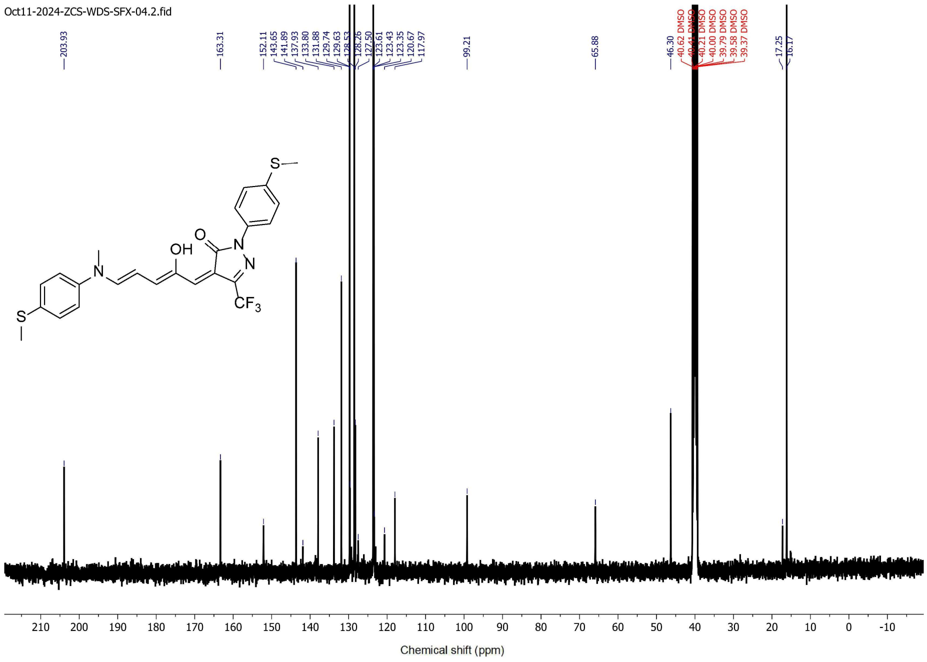

Supplementary Fig. 63 | ^{13}C NMR spectrum of SDAS (100 MHz, 298 K), [SDAS] = 10 mM, solvent: DMSO-*d*6.

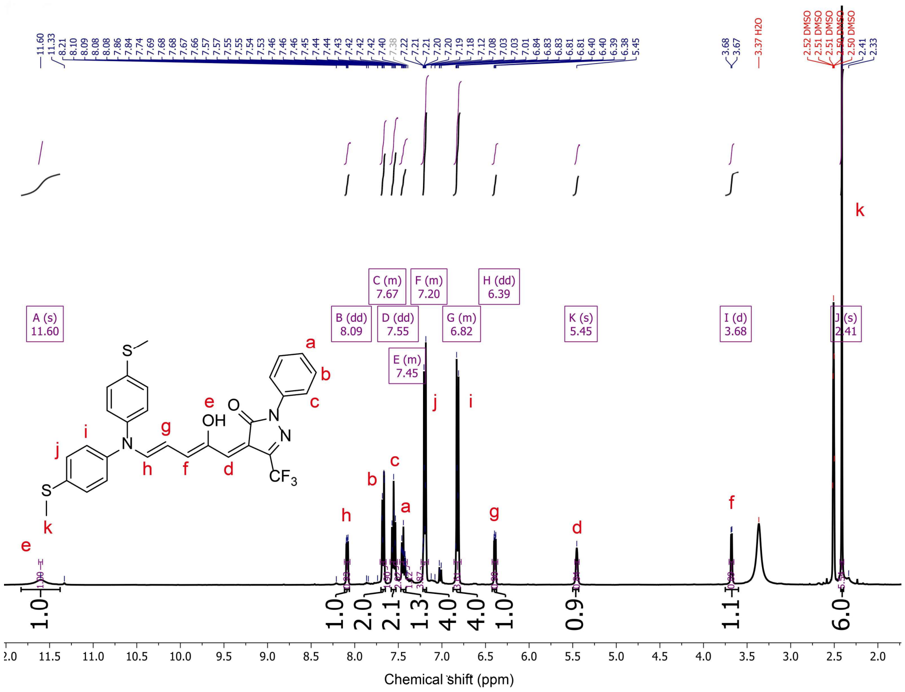

Supplementary Fig. 64 | ^{1}H NMR spectrum of SSDA (400 MHz, 298 K), [SSDA] = 10 mM, solvent: DMSO-*d*6.

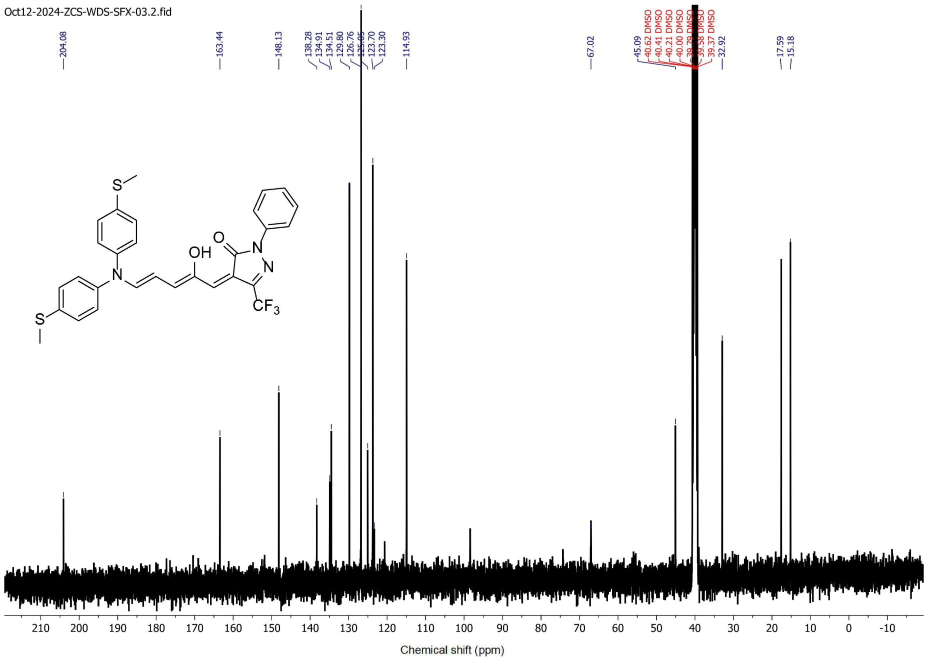

Supplementary Fig. 65 | ^{13}C NMR spectrum of SSDA (100 MHz, 298 K), [SSDA] = 10 mM, solvent: DMSO-*d*6.

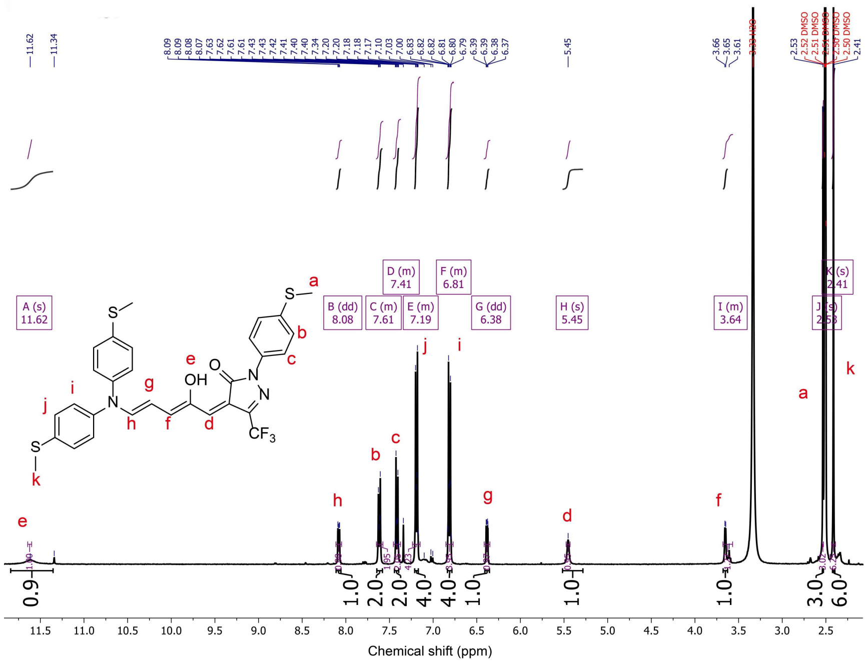

Supplementary Fig. 66 | ^{1}H NMR spectrum of SSDAS (400 MHz, 298 K), [SSDAS] = 10 mM, solvent: DMSO-*d*6.

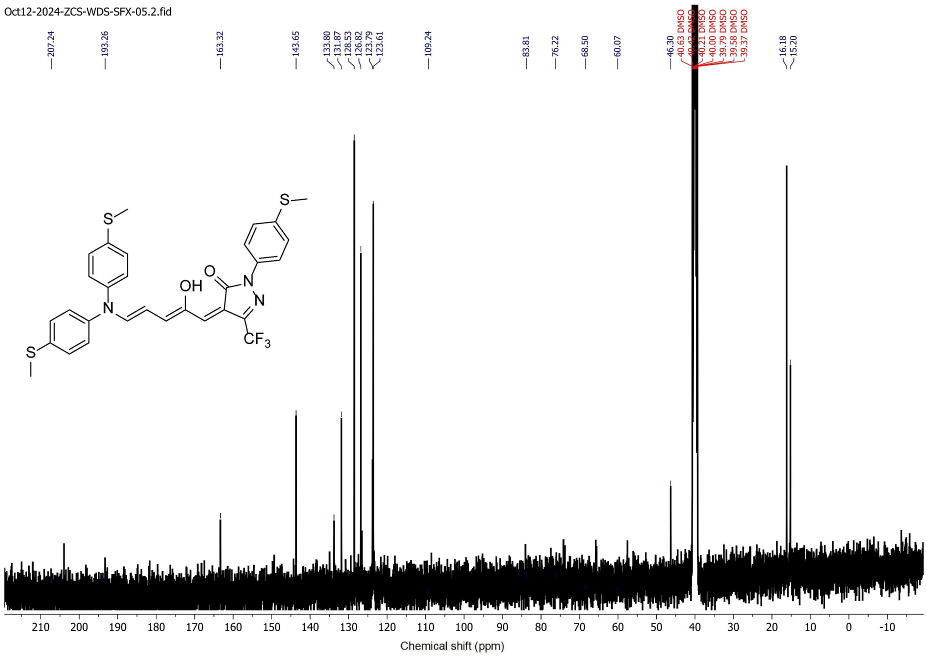

Supplementary Fig. 67 | ^{13}C NMR spectrum of SSDAS (100 MHz, 298 K), [SSDAS] = 10 mM, solvent: DMSO-*d*6.

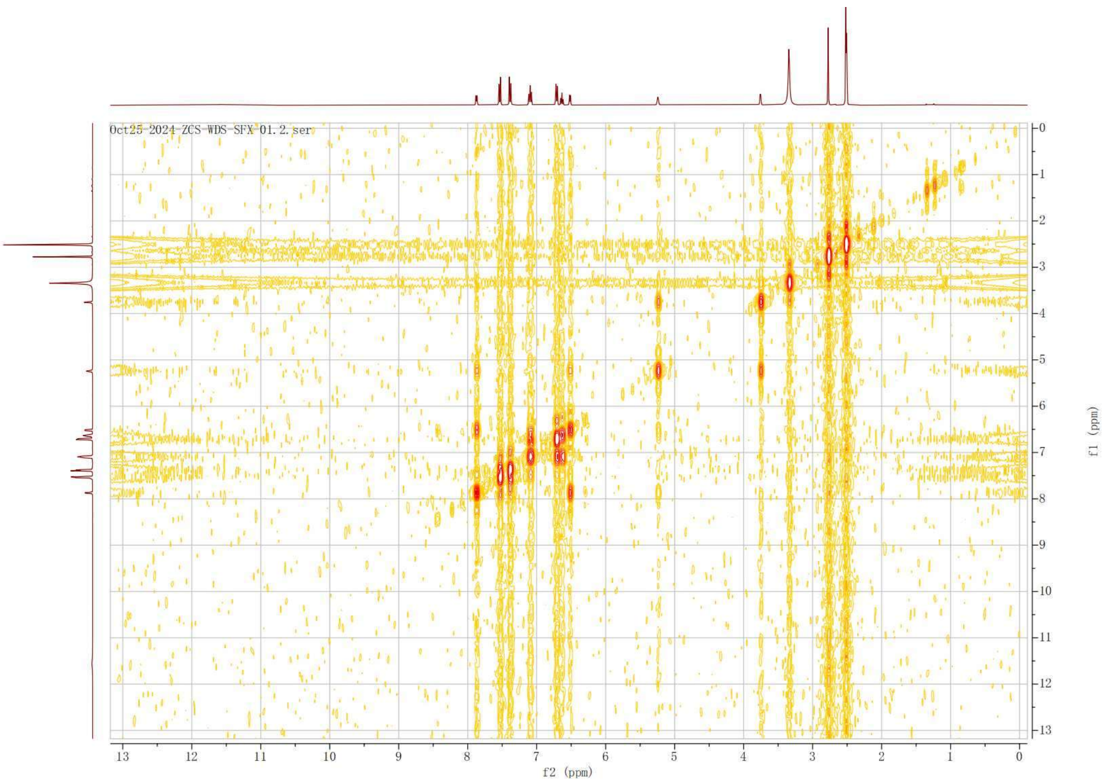

Supplementary Fig. 68 | 2D-COSY spectrum of DAS ([DAS] = 10 mM, in DMSO-*d*6).

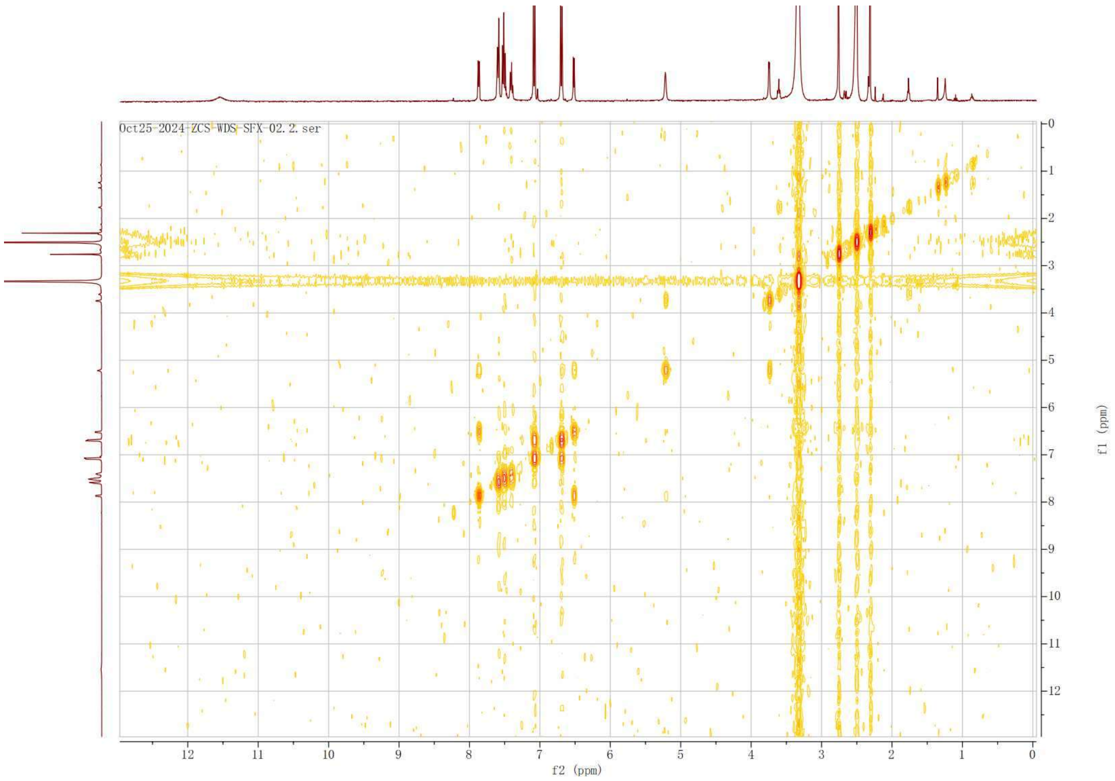

Supplementary Fig. 69 | 2D-COSY spectrum of SDA ([SDA] = 10 mM, in DMSO-*d*6).

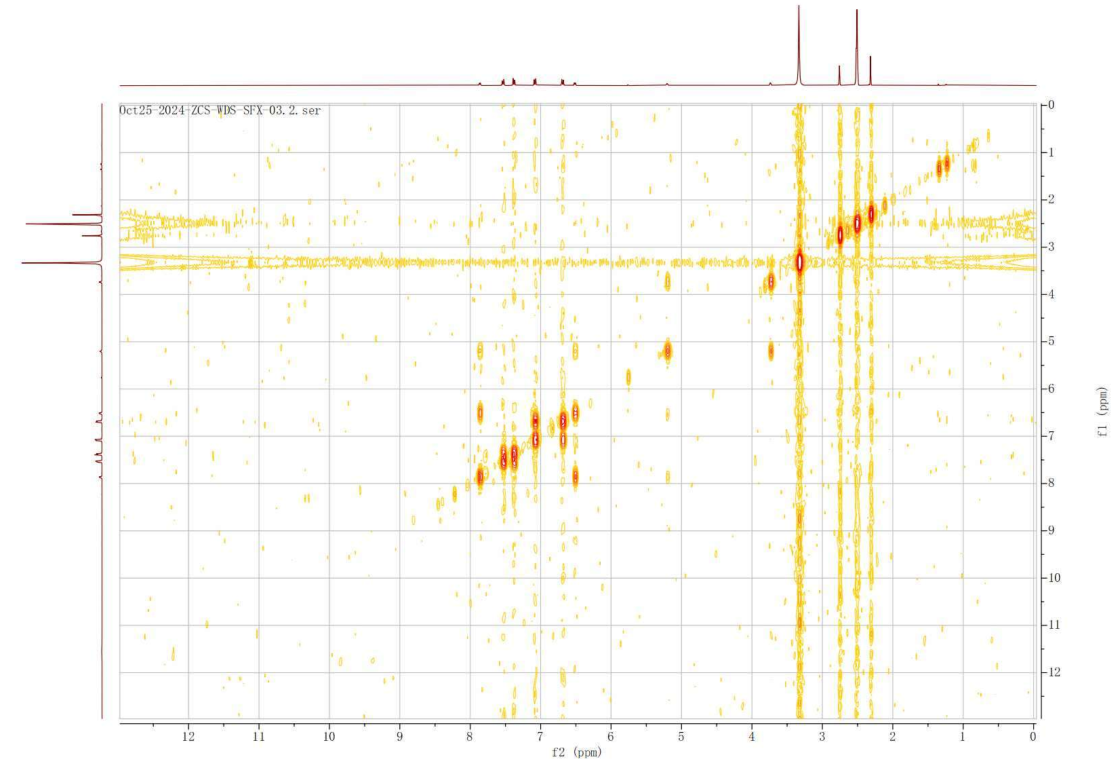

Supplementary Fig. 70 | 2D-COSY spectrum of SDAS ([SDAS] = 10 mM, in DMSO-*d*6).

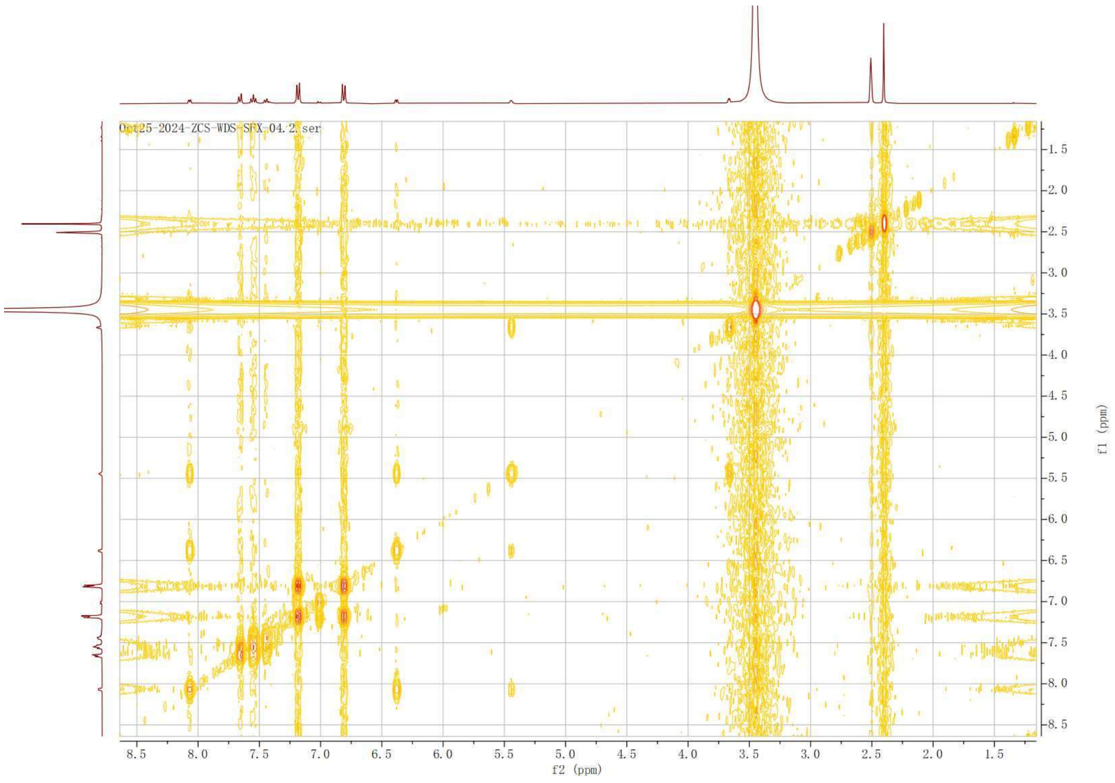

Supplementary Fig. 71 | 2D-COSY spectrum of SSDA ([SSDA] = 10 mM, in DMSO-*d*6).

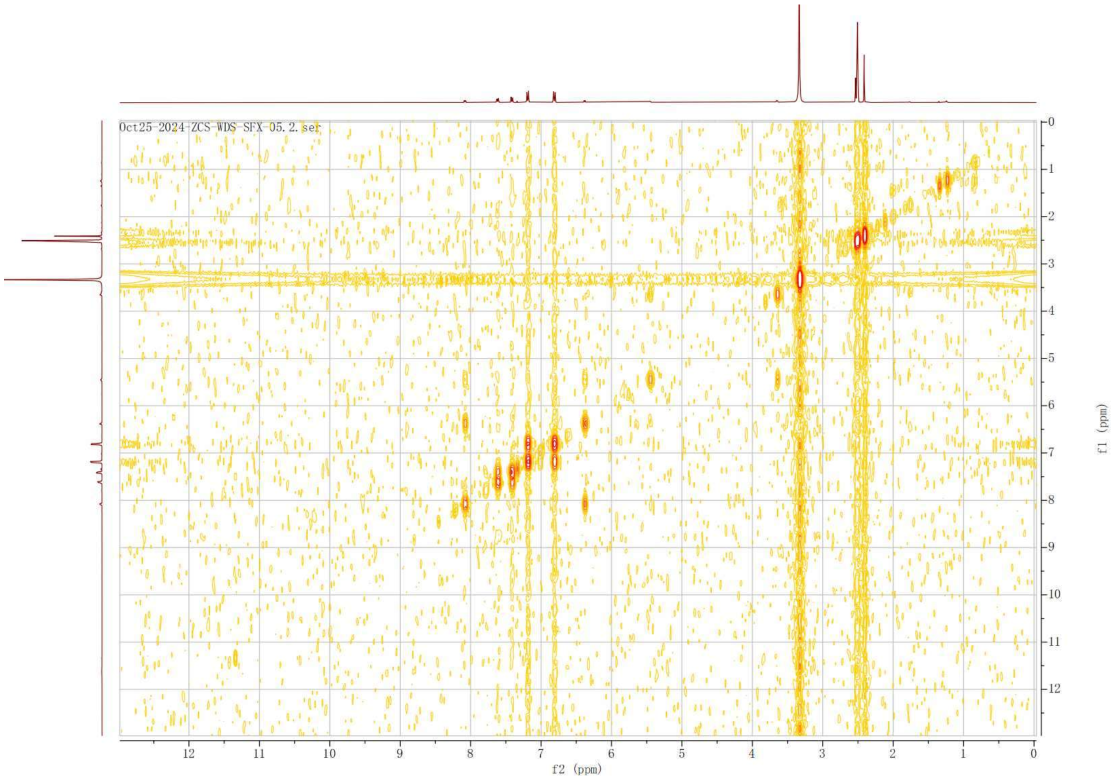

Supplementary Fig. 72 | 2D-COSY spectrum of SSDAS ([SSDAS] = 10 mM, in DMSO-*d6*).

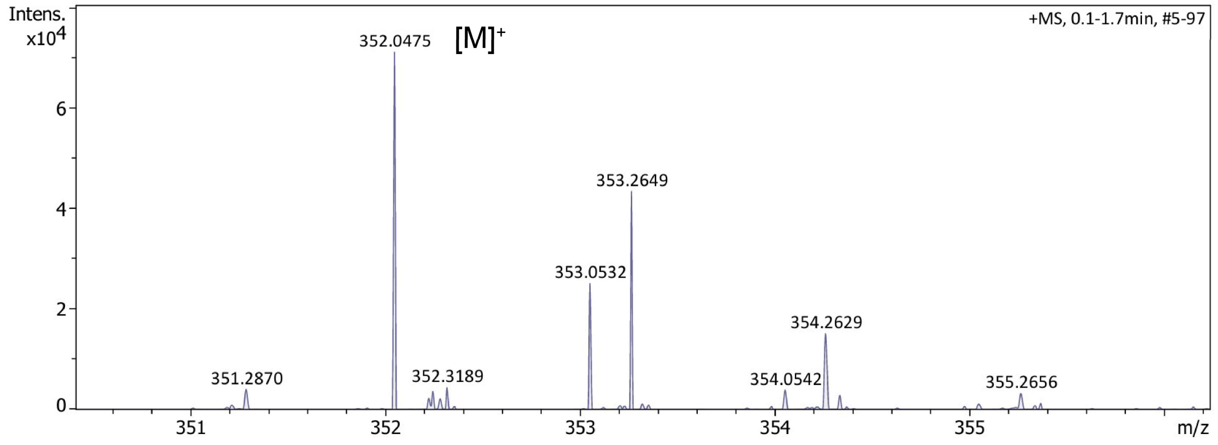

Supplementary Fig. 73 | High-resolution ESI mass spectra of intermediate 3.

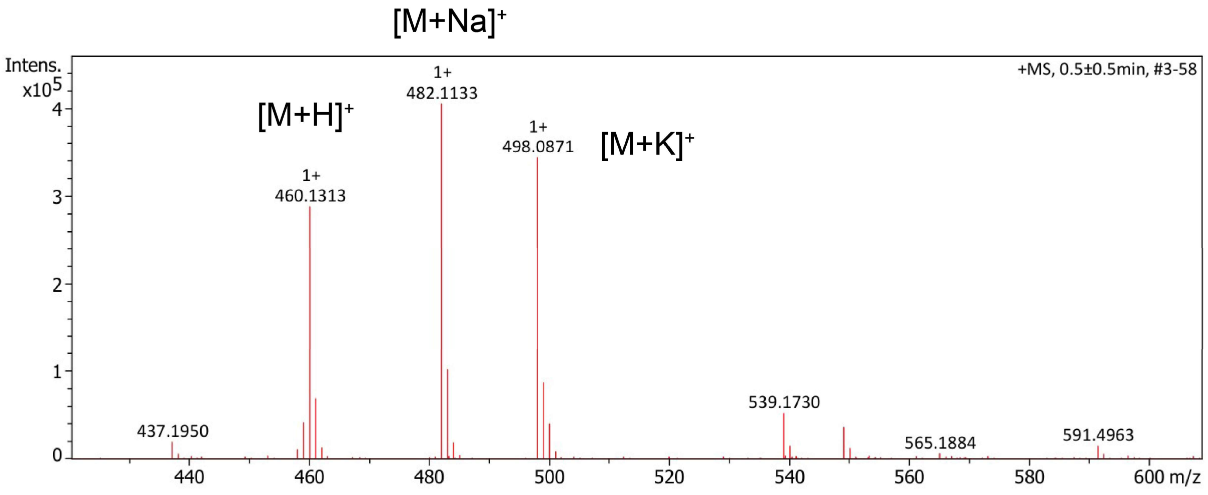

Supplementary Fig. 74 | High-resolution ESI mass spectra of DAS.

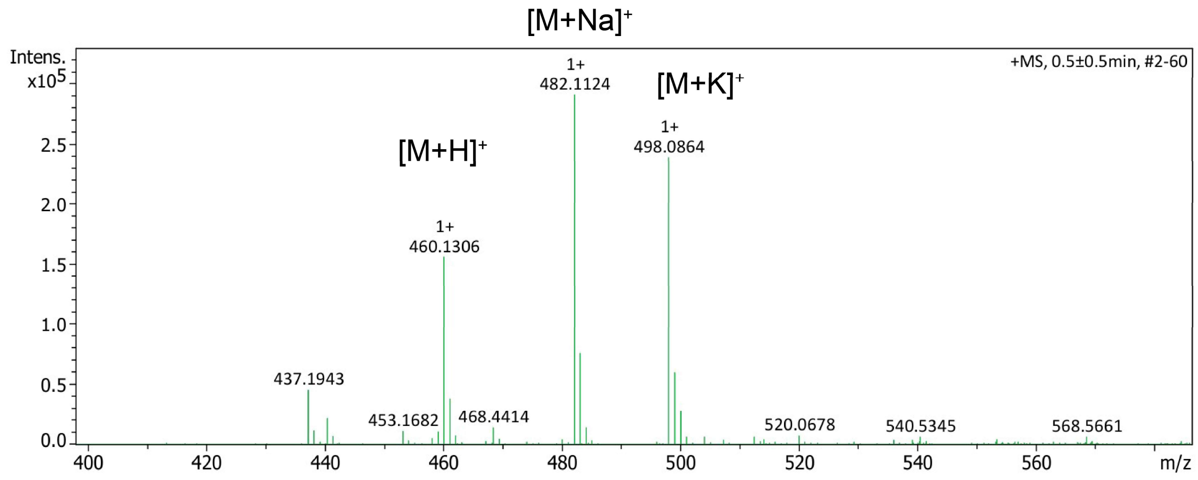

Supplementary Fig. 75 | High-resolution ESI mass spectra of SDA.

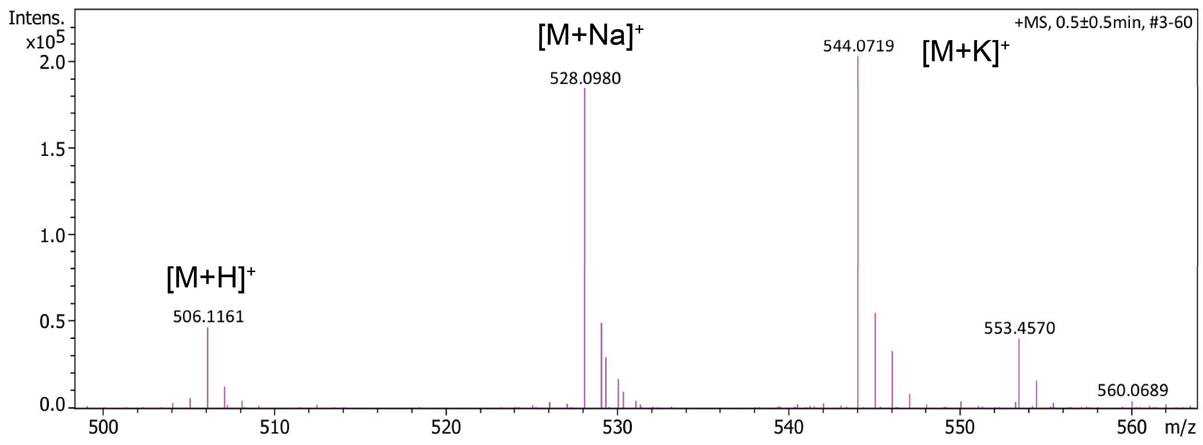

Supplementary Fig. 76 | High-resolution ESI mass spectra of SDAS.

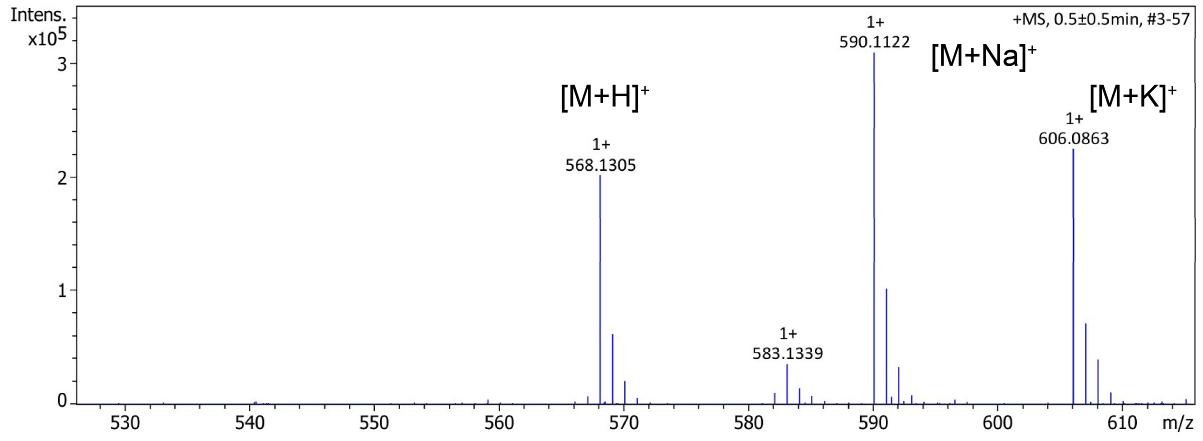

Supplementary Fig. 77 | High-resolution ESI mass spectra of SSDA.

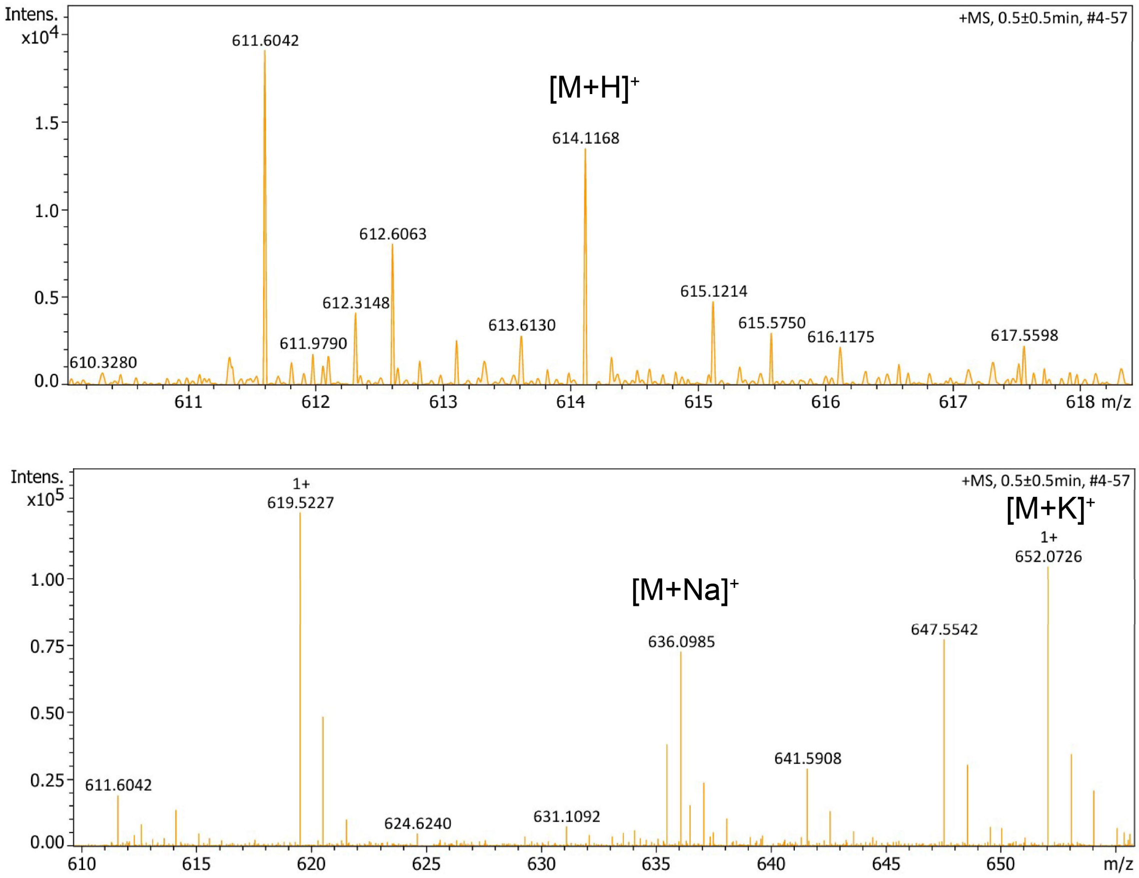

Supplementary Fig. 78 | High-resolution ESI mass spectra of SSDAS.

8 Cartesian coordinates

Supplementary Table 1. Cartesian coordinates of optimized DAS-A in Gaussian package.

N	-6.65354532	0.43828687	-0.01716405
C	-5.46240913	1.04103298	-0.00957907
C	-4.22464366	0.43212044	-0.01135818
C	-3.06861070	1.21921100	-0.00182208
C	-1.77787704	0.69858472	-0.01467498
C	-0.71030654	1.60134935	-0.01020287
C	0.66004523	1.35534696	-0.02556438
O	-1.66077897	-0.64457648	-0.03325915
C	-7.90090071	1.19300577	-0.05728013
C	-7.17003607	-1.69089991	-1.09610315
C	-6.76584260	-0.99150254	0.03591327
C	-7.29415084	-3.07459200	-1.03508361
C	-7.01032694	-3.74920023	0.14799983
C	-6.60182692	-3.04171691	1.27480160
C	-6.48095283	-1.65803961	1.22368653
C	1.70892992	2.32898492	-0.01956390
N	2.89561187	1.81316028	-0.04045922
N	2.71713086	0.45552679	-0.06428581
C	1.37642916	0.09145354	-0.05088215
O	0.95430622	-1.07042871	-0.05640568
C	3.85919208	-0.38284788	-0.05913654
C	3.76139283	-1.74888599	-0.33935855
C	4.90355997	-2.53336518	-0.33217369
C	6.15856922	-1.98608705	-0.05394229
C	6.24528671	-0.62252436	0.22100195
C	5.10570478	0.17234878	0.22070867
S	7.54486893	-3.09484674	-0.07597122
C	8.93787110	-1.99190286	0.25296072
C	1.54206965	3.81232878	0.00084367
F	0.84806229	4.20916313	1.08286052
F	0.84413205	4.23917024	-1.06672408
F	2.70373226	4.45690969	0.00796582
H	-5.51112699	2.12686756	-0.00818261
H	-4.13007319	-0.64661858	-0.02782329
H	-3.16909857	2.30022965	0.01479387
H	-1.00808361	2.64547851	0.00731192
H	-0.71742365	-0.92718750	-0.04003433
H	-7.68078917	2.25920519	-0.07699114
H	-8.47306197	0.93274780	-0.95008347
H	-8.50259948	0.96394561	0.82459431
H	-7.37079544	-1.15366274	-2.01679416
H	-7.60367603	-3.62551771	-1.91566240
H	-7.10275679	-4.82829519	0.19094398
H	-6.37563100	-3.56681679	2.19555885
H	-6.16044563	-1.09149276	2.09135310
H	2.80133137	-2.19143282	-0.55773018
H	4.81330394	-3.59189090	-0.55384394
H	7.19682757	-0.15591439	0.44266306
H	5.18268821	1.23022644	0.43270160
H	9.82707250	-2.62134823	0.22511897
H	9.02880058	-1.22516899	-0.51627666
H	8.86469568	-1.53698623	1.24053999

Supplementary Table 2. Cartesian coordinates of optimized DAS-TS (A-B) in Gaussian package.

C	1.45704700	-0.00069600	-0.68925900
C	1.18840000	2.03446500	0.16328800
C	0.50161400	1.03098500	-0.54387600
C	-0.86928500	1.05078500	-1.02114300
O	1.36973300	-1.12752600	-1.25929900
C	-1.56765400	0.01110400	-1.49769800
C	-2.97577200	0.17107400	-1.93662100
C	-4.00682700	-0.12050400	-1.12517800
C	-5.33391100	0.00003600	-1.63947200
O	-1.17624300	-1.28227400	-1.63417300
H	-5.46206000	0.41371300	-2.63657200
H	-3.83680800	-0.44554000	-0.10567800
H	-3.17073900	0.47843500	-2.96426800
H	-0.17626100	-1.34018600	-1.51321100
H	-1.38768700	2.00467900	-0.99002100
C	0.61994400	3.34349100	0.57951700
N	2.42918200	1.73389000	0.46432600
N	2.60462700	0.48193000	-0.05307700
C	3.85511100	-0.14910200	0.10403200
C	4.11417700	-1.41199000	-0.43799800
C	4.85683900	0.50942100	0.82583000
C	5.36248000	-1.99379300	-0.25682200
H	3.34470400	-1.92665700	-0.99158500
C	6.09631500	-0.08520200	0.99711900
H	4.64985900	1.48239800	1.24768900
C	6.36623900	-1.34154600	0.45456900
H	5.55594400	-2.97457000	-0.67686500
H	6.86401200	0.43098900	1.56282300
F	1.49031500	4.11222900	1.23139600
F	0.17778500	4.05289300	-0.48026400
F	-0.45267300	3.18696100	1.38192800
N	-6.42588500	-0.32638200	-1.01984500
C	-6.41852800	-0.96315600	0.27965700
C	-7.05982300	-0.34080100	1.34301100
C	-5.79668500	-2.19771400	0.42123800
C	-7.06247600	-0.96907500	2.58207300
H	-7.53067400	0.62664200	1.21176500
C	-5.80790700	-2.81458100	1.66571300
H	-5.30910900	-2.66352400	-0.42781000
H	-7.54766400	-0.48903300	3.42288000
H	-5.32014200	-3.77330700	1.79017100
S	7.96998700	-2.09586000	0.66449800
C	8.79762100	-1.40045500	-0.79965500
H	8.82259400	-0.31290700	-0.74421100
H	9.81842300	-1.78273500	-0.81020400
H	8.28792500	-1.71242300	-1.71028600
C	-6.43889300	-2.20196300	2.74313100
H	-6.44204100	-2.68537500	3.71236400
C	-7.74681900	-0.11417700	-1.62641200
H	-8.27889700	-1.06388700	-1.64583300
H	-8.30810900	0.60164400	-1.02732800
H	-7.62253200	0.26936600	-2.63563400

Supplementary Table 3. Cartesian coordinates of optimized DAS-B in Gaussian package.

C	2.75630300	-2.47848800	0.09861000
C	1.39730800	-2.18874100	0.05065100
C	0.95065400	-0.86196400	-0.03189300
C	-0.34091200	-0.35926400	-0.07371200
O	0.57811200	-3.26492500	0.10443000
C	-0.71519400	1.02334700	-0.14479800
N	-1.99348400	1.21244100	-0.16955600
N	-2.56479100	-0.03183100	-0.12054000
C	-1.62454100	-1.05130500	-0.05275300
O	-1.88393400	-2.25473000	0.01349500
C	-3.97802700	-0.13517200	-0.08890500
C	-4.62288300	-1.34841600	-0.34440300
C	-6.00637400	-1.40975200	-0.30564000
C	-6.77521300	-0.27770200	-0.02281300
C	-6.12171200	0.92863500	0.22295100
C	-4.73438800	0.99923300	0.19371600
S	-8.53777700	-0.48524500	-0.00512800
C	-9.12799300	1.17442000	0.39798900
C	3.86113100	-1.61093700	0.03613600
C	5.12765100	-2.13525700	0.12022900
N	6.29967400	-1.47352900	0.11611000
C	6.37804500	-0.04947600	0.11718700
C	7.54368800	-2.22292400	-0.04441100
C	7.16197000	0.60277000	-0.83227200
C	7.26040000	1.98979900	-0.81039500
C	6.57789500	2.72710100	0.15045000
C	5.80218000	2.07075200	1.10227800
C	5.70589000	0.68561000	1.09455700
C	0.21365800	2.19174500	-0.19102200
F	1.05252800	2.10469700	-1.23784000
F	0.99025400	2.23966000	0.90661300
F	-0.42954400	3.35012000	-0.28123600
H	2.97355800	-3.54081200	0.20005800
H	1.73832600	-0.11691000	-0.05887300
H	-0.36897800	-3.00732400	0.08057500
H	-4.04764000	-2.23495500	-0.56502200
H	-6.49448800	-2.35835000	-0.50446400
H	-6.67739600	1.83091500	0.44492200
H	-4.23526800	1.93984500	0.38470500
H	-10.21521000	1.10391900	0.42215400
H	-8.77773100	1.49595400	1.37868300
H	-8.84279600	1.89664800	-0.36668600
H	3.73210900	-0.54311900	-0.08548700
H	5.23673800	-3.21410700	0.19085700
H	7.41804600	-3.21924200	0.37838000
H	7.82101400	-2.32080900	-1.09727600
H	8.34346200	-1.71254800	0.49039300
H	7.68362100	0.03058700	-1.59129300
H	7.86742100	2.49345100	-1.55397800
H	6.65226500	3.80821900	0.16140300
H	5.27625200	2.63751600	1.86199100
H	5.12155100	0.16831700	1.84726400

Supplementary Table 4. Cartesian coordinates of optimized DAS-TS (B-B’) in Gaussian package.

C	-1.49135800	1.16118900	-0.22629400
C	-0.45428400	-0.83635100	-0.42614900
C	-0.18200500	0.56392300	-0.37958000
C	1.07956400	1.15150300	-0.42044500
O	-1.82489200	2.35094500	-0.15674500
C	1.44694000	2.50094900	-0.36134000
C	2.80096500	2.85127000	-0.31428700
C	3.84478500	2.02128000	0.10307000
C	3.62867300	0.95438700	0.96230800
O	0.56583400	3.53104300	-0.44222800
H	2.69387200	0.91473100	1.51360900
H	4.85900000	2.25281800	-0.20457300
H	3.03581800	3.86685700	-0.62194900
H	-0.36368700	3.21222700	-0.40818400
H	1.90461500	0.46926000	-0.58640600
C	0.57578600	-1.91055200	-0.51019600
N	-1.71120400	-1.12779800	-0.31110400
N	-2.36126000	0.07525000	-0.19737900
C	-3.77473000	0.08931300	-0.11589200
C	-4.46130100	1.22387400	0.31513100
C	-4.48385200	-1.06079600	-0.46760700
C	-5.84814100	1.19527700	0.39484000
H	-3.92057600	2.11452100	0.59647300
C	-5.86723200	-1.07022800	-0.39724900
H	-3.94723200	-1.93760200	-0.80265400
C	-6.56539000	0.05604800	0.03946000
H	-6.37524800	2.07270000	0.75146500
H	-6.40282400	-1.96302300	-0.69910300
F	0.05827200	-3.13110500	-0.48612700
F	1.31329800	-1.79713200	-1.62799300
F	1.44933300	-1.81668500	0.51726700
N	4.48478300	-0.02584700	1.26741000
C	5.68864900	-0.25455000	0.53456400
C	6.87770600	-0.49077700	1.21933600
C	5.65985800	-0.29610300	-0.85947700
C	8.04229000	-0.75086000	0.50578500
H	6.89643900	-0.46314300	2.30271300
C	6.82839800	-0.55226500	-1.56197600
H	4.72279900	-0.14519900	-1.38354500
C	8.02297000	-0.77752900	-0.88329800
H	6.80148300	-0.58900400	-2.64454100
S	-8.34472200	0.09511400	0.10607000
C	-8.67929600	-1.46939600	0.96622200
H	-8.09517600	-1.52873900	1.88317000
H	-9.73977100	-1.46633200	1.21702100
H	-8.47595400	-2.33133600	0.33340800
H	8.96697300	-0.92972400	1.04150400
C	4.21292500	-0.89129700	2.41561400
H	4.75172200	-0.55512100	3.30349800
H	3.14509500	-0.87737000	2.62705200
H	4.51263700	-1.91029100	2.17591000
H	8.93215700	-0.98075800	-1.43625000

Supplementary Table 5. Cartesian coordinates of optimized DAS-B’ in Gaussian package.

C	-2.67516300	-2.61467300	0.45576300	C	-7.28787400	0.23503000	1.64238700
C	-1.32482900	-2.22423700	0.41381800	C	-6.08562600	0.15632200	0.94264400
C	-0.96683400	-0.86127000	0.37316700	C	-0.40241500	2.25721100	0.31221700
C	0.29730100	-0.26865100	0.30558900	F	-1.25445800	2.15167400	1.36438200
O	-0.43219000	-3.23366500	0.52393300	F	-1.18331900	2.23910700	-0.80248300
C	0.58747600	1.13627900	0.29771600	F	0.18114800	3.46145500	0.37729600
N	1.86375500	1.40296700	0.19387500	H	-2.83424000	-3.62963600	0.81843900
N	2.50508300	0.18255000	0.14756600	H	-1.79975700	-0.17522600	0.46948000
C	1.61218200	-0.88871200	0.21054600	H	0.50352300	-2.89143400	0.44741300
O	1.93214300	-2.09702500	0.20327100	H	4.09486100	2.28751700	0.36988400
C	3.92307400	0.16065600	0.06795000	H	6.55030600	2.31198200	0.22660600
C	4.63422800	1.36332800	0.20340800	H	6.53351300	-1.95484900	-0.39440100
C	6.02014700	1.36888600	0.11999100	H	4.09095200	-1.96284800	-0.25163200
C	6.73665900	0.18029100	-0.09560300	H	10.15281300	-1.35681200	-0.54455900
C	6.02077600	-1.01398200	-0.22709700	H	8.78304600	-2.06617500	0.33811400
C	4.62701100	-1.02959100	-0.14933900	H	8.67313200	-1.78657200	-1.43094100
S	8.51642700	0.31189200	-0.17987300	H	-4.77865900	-2.24729800	0.46222300
C	9.06162100	-1.40093100	-0.48408800	H	-2.92336000	-0.65947700	-1.40769500
C	-3.82519300	-1.89735200	0.07502100	H	-3.73348100	0.96535000	-2.61085900
C	-3.82739000	-0.85593800	-0.83750100	H	-5.16391500	0.15086100	-3.28224300
N	-4.86998700	-0.06341400	-1.18011700	H	-5.35078600	1.63820000	-2.31533400
C	-6.09797000	-0.02054500	-0.44852700	H	-7.33574200	-0.24224900	-2.20505900
C	-4.78027900	0.71359800	-2.42268300	H	-9.46100800	-0.08114300	-0.95486200
C	-7.31992400	-0.10070900	-1.12898800	H	-9.44267300	0.21683200	1.51597200
C	-8.51811900	-0.01390000	-0.41919600	H	-7.26872400	0.37808000	2.71922900
C	-8.50801100	0.15034700	0.96648600	H	-5.13963200	0.24655300	1.46643900

Supplementary Table 6. Cartesian coordinates of optimized DAS-TS (B’-C) in Gaussian package.

C	-1.18949700	1.08638700	-0.32578100	H	-5.95515300	1.86751000	1.27074900
C	-0.10300200	-0.81677300	-0.43649600	H	-6.08571200	-1.95805600	-0.69087000
C	0.10352600	0.58217400	-0.49990900	F	0.52537300	-3.08188100	-0.38949000
C	1.35816500	1.26583400	-0.68098700	F	1.62216300	-1.77142200	-1.70773000
O	-1.64016000	2.32101800	-0.32587500	F	1.91567500	-1.63634700	0.41615100
C	1.48979500	2.67095200	-0.76115800	N	3.85138300	0.35456700	1.34933200
C	2.87685400	3.11623800	-0.75707400	C	4.96174800	-0.22881700	0.66823400
C	3.75238900	2.39202700	-0.01834800	C	6.01659600	-0.74873700	1.41633800
C	3.21350900	1.43761900	0.90944800	C	4.97907100	-0.32333400	-0.72339900
O	0.52810100	3.50527200	-0.78585900	C	7.09157900	-1.34767900	0.77154100
H	2.35852000	1.72894600	1.50427400	H	6.00141500	-0.67823800	2.49776600
H	4.82997100	2.50982900	-0.08695000	C	6.06035800	-0.92006900	-1.35714500
H	3.15803300	4.01627700	-1.29201700	H	4.15061300	0.05061300	-1.31161100
H	-0.82740600	2.91981200	-0.54354700	C	7.11991200	-1.43243000	-0.61542600
H	2.16959000	0.65903100	-1.05193300	H	7.90985300	-1.74643000	1.35926800
C	0.97268600	-1.83968900	-0.53177700	H	6.06387400	-0.99655500	-2.43784500
N	-1.35178600	-1.16286500	-0.23757600	S	-7.95881100	0.02310000	0.56095700
N	-2.02102000	0.01762300	-0.17272100	C	-8.50920000	-1.56416100	-0.10181100
C	-3.43369500	0.01835200	-0.01119400	H	-9.58768400	-1.59038200	0.04823100
C	-4.08197800	1.07612100	0.62455500	H	-8.30372900	-1.64451700	-1.16856300
C	-4.16446100	-1.06604800	-0.47928700	H	-8.06100500	-2.39713800	0.43875700
C	-5.45780600	1.04116700	0.77458300	H	7.95816000	-1.90180200	-1.11594700
H	-3.51778000	1.91812400	0.99956900	C	3.36206900	-0.32450900	2.55303400
C	-5.54341400	-1.09993400	-0.31587700	H	3.49422700	-1.39842700	2.44115300
H	-3.65119700	-1.88571500	-0.96440100	H	3.89714300	0.01932100	3.44019100
C	-6.20590300	-0.04418200	0.30902500	H	2.30095000	-0.11360700	2.67214500

Supplementary Table 7. Cartesian coordinates of optimized DAS-C in Gaussian package.

C	0.48125600	-0.04222100	0.36399500	S	-7.57848100	0.05367800	-0.68121100
C	0.18828000	1.32922800	0.53798600	C	-8.11915700	-1.36875300	0.29306700
N	-1.09376900	1.61404300	0.41965900	C	1.15151300	2.44554500	0.76964700
N	-1.68032300	0.42428900	0.18347200	F	1.71882400	2.85975500	-0.38331800
C	-0.76509500	-0.57929800	0.13041200	F	2.15985800	2.07075600	1.56951700
O	-1.05571800	-1.86523100	-0.11001600	F	0.56942800	3.51024500	1.31536900
C	-3.08582900	0.31882300	0.00582300	H	-1.94236700	-1.96132800	-0.48523200
C	-3.74771700	1.24101400	-0.80324300	H	-3.18463600	2.03799000	-1.27363100
C	-5.11424600	1.13400000	-0.97857900	H	-5.62937500	1.85679000	-1.60246900
C	-5.83775500	0.09906200	-0.36774400	H	-5.69452600	-1.60952800	0.94880800
C	-5.16436400	-0.81348000	0.44243000	H	-3.28424300	-1.37202800	1.32109100
C	-3.79091800	-0.69684300	0.63941000	H	2.57074300	-0.16434200	0.75965100
C	1.75216200	-0.81844700	0.43994400	H	2.60490800	-4.00587100	1.49967200
C	1.68876500	-1.94336200	1.49159600	H	3.20425800	-3.57369800	-1.02028400
C	2.41406900	-3.10536200	0.93012600	H	1.33284800	-1.75027800	-1.47974700
C	2.71083300	-2.87931900	-0.34938100	H	5.17768300	-2.36795700	-0.97554600
C	2.21029700	-1.54750100	-0.85540600	H	7.22768700	-1.68200400	0.20117500
O	1.12368000	-1.88120000	2.55119200	H	7.62646000	0.71309400	0.72634700
C	4.34849300	-0.38753900	-1.06536100	H	5.92554600	2.40089800	0.07576800
C	5.32297000	-1.32801500	-0.71038800	H	3.85354300	1.71369200	-1.03505200
C	6.48630700	-0.93546500	-0.06211300	H	3.21114400	0.64908100	-3.19143200
C	6.71395300	0.40707500	0.22755900	H	1.73676600	-0.32343700	-3.14897700
C	5.76322000	1.35057000	-0.14045500	H	1.97830400	0.95945100	-1.95196100
C	4.58723500	0.96038300	-0.77520800	H	-9.19511200	-1.44043000	0.13668400
N	3.15599600	-0.82726500	-1.71092000	H	-7.65503300	-2.29139300	-0.05479800
C	2.48439700	0.17620700	-2.53001400	H	-7.93094100	-1.21818600	1.35569200

Supplementary Table 8. Cartesian coordinates of optimized SDA-A in Gaussian package.

N	4.50275500	-2.37699200	-0.04894800
C	3.17046000	-2.52750600	-0.04056000
C	2.20803100	-1.54262400	-0.03379300
C	0.85368200	-1.90020900	-0.00554100
C	-0.18773200	-0.97983300	-0.01831900
C	-1.49701900	-1.47591000	0.00252500
C	-2.70668000	-0.79076400	-0.00570200
O	0.14701600	0.32568700	-0.05065300
C	5.37065100	-3.53203400	-0.26813300
C	6.06487500	-0.70506600	-0.90223400
C	5.12974000	-1.09745400	0.04711400
C	6.70954100	0.52296400	-0.78971400
C	6.42042600	1.37370600	0.27616000
C	5.47836600	0.96912500	1.23139200
C	4.84414800	-0.25476900	1.12311000
C	-4.01805600	-1.36400700	0.01743500
N	-4.96713400	-0.48655500	0.00124400
N	-4.35442800	0.73901100	-0.03415200
C	-2.96744200	0.63952000	-0.04112200
O	-2.18175500	1.59190500	-0.07184800
C	-5.16452000	1.90315400	-0.06051500
C	-4.60006800	3.18108100	-0.08144200
C	-5.43524000	4.29319800	-0.10648200
C	-6.81746600	4.15339300	-0.11069800
C	-7.36859100	2.87606200	-0.08943000
C	-6.55420600	1.75279800	-0.06453000
S	7.17405300	2.95623000	0.52245100
C	8.29302800	3.08846300	-0.88995000
C	-4.34858400	-2.81939000	0.06172500
F	-3.81540300	-3.47074400	-0.98728500
F	-5.65724400	-3.04589100	0.05611300
F	-3.83683000	-3.40074800	1.16106900
H	2.84618800	-3.56497300	-0.04911200
H	2.47162200	-0.49301800	-0.06588300
H	0.58879500	-2.95265200	0.02693100
H	-1.56291400	-2.55955800	0.02845400
H	-0.64879600	0.90486000	-0.06263600
H	4.83650800	-4.44195900	0.00105100
H	5.67821600	-3.60328700	-1.31437100
H	6.25702800	-3.44191900	0.35900400
H	6.29085900	-1.35131000	-1.74309500
H	7.42931400	0.80311100	-1.54806600
H	5.24759600	1.61587100	2.07130500
H	4.12773700	-0.56507600	1.87526100
H	-3.52780400	3.30035300	-0.07846300
H	-4.98857700	5.28124300	-0.12249700
H	-7.45812200	5.02756600	-0.13002500
H	-8.44517200	2.74617400	-0.09222500
H	-6.98241900	0.76029500	-0.04762900
H	8.78519800	4.05493800	-0.78494900
H	9.05271900	2.30737900	-0.87269300
H	7.74797200	3.07635400	-1.83342500

Supplementary Table 9. Cartesian coordinates of optimized SDA-TS (A-B) in Gaussian package.

C	-3.12030300	-0.14371500	-0.82789300
C	-3.08001700	-1.00229800	1.22142200
C	-2.34603700	-0.96653100	0.02173600
C	-1.09000000	-1.62626800	-0.28584300
O	-2.92452400	0.21427300	-2.02810500
C	-0.29685600	-1.37649100	-1.33696400
C	0.97052900	-2.11997200	-1.53994500
C	2.15766500	-1.62480700	-1.14859700
C	3.33301000	-2.38095600	-1.44182900
O	-0.46480900	-0.45871800	-2.32389000
H	3.20757800	-3.36024800	-1.89579000
H	2.22160100	-0.67794900	-0.62562000
H	0.93804300	-3.06862900	-2.07650800
H	-1.41908800	-0.12248900	-2.30479400
H	-0.75647100	-2.40084300	0.39870500
C	-2.69310000	-1.73953300	2.45184500
N	-4.18981000	-0.30197100	1.19763200
N	-4.22254700	0.22933600	-0.05872900
C	-5.29382800	1.07838400	-0.41670000
C	-5.51887900	1.44501800	-1.74630400
C	-6.14543400	1.55069600	0.58630000
C	-6.58555900	2.28237600	-2.05321000
H	-4.86323000	1.07801500	-2.52079900
C	-7.20775200	2.38063600	0.25888400
H	-5.96616600	1.25589400	1.61073400
C	-7.43552800	2.75622400	-1.06133500
H	-6.75073100	2.56044400	-3.08828100
H	-7.86041100	2.73839900	1.04749400
F	-3.57473600	-1.61148200	3.44220900
F	-2.54843000	-3.06128300	2.21660300
F	-1.49944100	-1.32645000	2.92584400
N	4.55774500	-2.02255100	-1.20461100
C	4.89835400	-0.71554000	-0.69455500
C	5.61869700	-0.60611000	0.48496800
C	4.52624100	0.41884500	-1.41172900
C	5.95625900	0.65111600	0.96905100
H	5.90226700	-1.49075300	1.04344800
C	4.86528300	1.66812500	-0.92792100
H	3.97202800	0.32269400	-2.33887800
C	5.58158000	1.80049900	0.26976000
H	6.50729600	0.71653000	1.89762000
H	4.56871300	2.55149100	-1.48211600
S	5.95337400	3.44255900	0.79113400
C	6.77145300	3.17986800	2.37968800
H	6.99129300	4.17545000	2.76242800
H	6.11307800	2.67386100	3.08459600
H	7.70880200	2.63748700	2.26411600
H	-8.26507700	3.40712300	-1.31217000
C	5.68553600	-2.91952000	-1.49047400
H	6.36319200	-2.42123200	-2.18191000
H	6.21363600	-3.13851000	-0.56342300
H	5.31217500	-3.84316200	-1.92509400

Supplementary Table 10. Cartesian coordinates of optimized SDA-B in Gaussian package.

C	0.87090880	3.20396331	-0.10762234
C	-0.42592275	2.70076322	-0.05519121
C	-0.64626815	1.32117882	0.05878326
C	-1.83242998	0.60335793	0.08558262
O	-1.41094469	3.62440281	-0.14533427
C	-1.96255876	-0.82099924	0.18194630
N	-3.18861696	-1.22934715	0.17259671
N	-3.96674222	-0.10591017	0.07126268
C	-3.21364481	1.06117543	0.00686204
O	-3.66958641	2.20138017	-0.09980618
C	-5.37552902	-0.25581286	0.00329773
C	-6.22590247	0.84370348	0.13666741
C	-7.60146989	0.65091136	0.06616999
C	-8.13851394	-0.61572402	-0.12886510
C	-7.28149143	-1.70444865	-0.25538056
C	-5.90575050	-1.53320943	-0.19329668
C	2.08448990	2.50464250	-0.01208185
C	3.28352628	3.16560204	-0.12442626
N	4.51001620	2.61176182	-0.11126120
C	4.68671506	1.19623824	-0.07083134
C	5.69586351	3.45263700	0.02020130
C	5.43128933	0.60323707	0.94713177
C	5.57014375	-0.77559289	0.99196374
C	4.96630990	-1.58518005	0.02471648
C	4.24528788	-0.98269612	-1.00836767
C	4.11367845	0.39806560	-1.05692290
S	5.17962461	-3.33630074	0.18030999
C	3.91978961	-3.97936080	-0.94841876
C	-0.84935225	-1.80980427	0.30337644
F	-0.11992538	-1.59102456	1.40983163
F	0.00708230	-1.70975458	-0.73268560
F	-1.28402002	-3.06392260	0.34484195
H	0.92416357	4.28241420	-0.24912185
H	0.25364645	0.72000140	0.12138426
H	-2.30085259	3.21028182	-0.13668689
H	-5.81700880	1.83078129	0.28903081
H	-8.25608866	1.50913064	0.16998510
H	-9.21229739	-0.75447818	-0.18052419
H	-7.68314644	-2.70020735	-0.40693242
H	-5.23729451	-2.37775017	-0.28983243
H	2.09345528	1.43522943	0.15110646
H	3.28416055	4.24645191	-0.23590162
H	5.47415703	4.44901686	-0.36041192
H	6.01128321	3.53850810	1.06345249
H	6.50925990	3.02385398	-0.56416806
H	5.88005275	1.21552747	1.72131121
H	6.13054669	-1.22973375	1.80230899
H	3.78385902	-1.57760585	-1.78625172
H	3.55569012	0.86101129	-1.86342143
H	3.89845319	-5.05650017	-0.78527860
H	2.93847284	-3.56842951	-0.71075145
H	4.17390028	-3.79205743	-1.99097829

Supplementary Table 11. Cartesian coordinates of optimized SDA-TS (B-B') in Gaussian package.

C	-3.28602100	0.84951300	-0.46265200
C	-1.89590100	-0.70566100	0.47525400
C	-1.86863100	0.61379800	-0.11870400
C	-0.76864700	1.38889900	-0.28717100
O	-3.81674700	1.82647400	-0.97198100
C	-0.61975400	2.69372300	-0.88766200
C	0.62434500	3.22012500	-0.98107900
C	1.86115500	2.56269800	-0.51064800
C	2.39205100	2.81118000	0.69212300
O	-1.66668300	3.39683800	-1.38424100
H	1.94794200	3.57001300	1.33264000
H	2.33226800	1.84255200	-1.17576200
H	0.69461300	4.18277500	-1.48449800
H	-2.51735300	2.92981700	-1.27172000
H	0.17132900	0.97570600	0.06652200
C	-0.71497900	-1.44900900	1.01109100
N	-3.07096900	-1.22151800	0.50520500
N	-3.92803500	-0.30842900	-0.06362600
C	-5.31868700	-0.60050200	-0.11620900
C	-6.14187200	0.04731700	-1.03544900
C	-5.84007100	-1.55094500	0.75963500
C	-7.49710400	-0.26030300	-1.06134400
H	-5.73271100	0.77919100	-1.71609300
C	-7.19408100	-1.85123200	0.71205500
H	-5.18627600	-2.05020000	1.46191100
C	-8.02939500	-1.20625300	-0.19387500
H	-8.13703400	0.24430900	-1.77559100
H	-7.59612000	-2.59228400	1.39287700
F	-1.04418000	-2.62348100	1.52551500
F	0.19671500	-1.65573800	0.04610700
F	-0.09976200	-0.73078800	1.96444100
N	3.48777200	2.17699600	1.27582600
C	4.01848300	1.01235600	0.66803300
C	5.35128100	0.90882000	0.29011600
C	3.18403800	-0.10028000	0.48660300
C	5.84683300	-0.27297700	-0.26473100
H	6.02433600	1.74808900	0.42092000
C	3.66582300	-1.26316400	-0.07587400
H	2.15275000	-0.04650500	0.81625400
C	5.01079400	-1.36465600	-0.46339000
H	2.99802300	-2.10921400	-0.20202100
H	6.89259200	-0.31328000	-0.54165100
S	5.52387600	-2.90746200	-1.17071500
C	7.27854000	-2.62253700	-1.48676600
H	7.65660000	-3.54599500	-1.92330900
H	7.42881900	-1.81259000	-2.19974700
H	7.82203200	-2.42485800	-0.56330600
H	-9.08661100	-1.44125700	-0.22436100
C	4.40906000	3.05586800	1.98130700
H	5.01573600	3.66797000	1.30379900
H	3.83513700	3.72677200	2.62148000
H	5.06946800	2.46194100	2.61249500

Supplementary Table 12. Cartesian coordinates of optimized SDA-B' in Gaussian package.

C	-0.75551800	-2.67535000	-0.25198300	S	-8.09195500	0.29721200	-1.49886300
C	0.59882300	-2.29499300	-0.26016400	C	-9.41108200	0.26147400	-0.24097600
C	0.96907900	-0.93569800	-0.22336100	C	1.55733600	2.17788700	-0.15333500
C	2.23950800	-0.35207300	-0.20261800	F	0.82185300	2.15077200	0.99165700
O	1.47796000	-3.31089200	-0.41214700	F	0.66314200	2.09126000	-1.17158300
C	2.53941500	1.05047400	-0.19268600	F	2.14599400	3.37928600	-0.22670900
N	3.82044100	1.30851800	-0.14100300	H	-0.93559700	-3.68645900	-0.61559000
N	4.45594300	0.08391500	-0.13407900	H	0.13825200	-0.24278400	-0.27987800
C	3.55272800	-0.98163200	-0.16996900	H	2.41852700	-2.97579000	-0.37479000
O	3.86244000	-2.19195400	-0.18945200	H	6.03333100	-2.07461700	0.18655200
C	5.87698600	0.05407500	-0.11682800	H	8.50463700	-2.07007200	0.22191300
C	6.57680500	-1.14820700	0.06521200	H	9.76706200	0.05865200	-0.06224000
C	7.97193600	-1.13305400	0.08100200	H	8.50960500	2.18824600	-0.38092500
C	8.68055400	0.05811300	-0.07714200	H	6.03515700	2.18033900	-0.41635800
C	7.97514900	1.25009000	-0.25521300	H	-2.85510200	-2.29764500	-0.17246400
C	6.58246700	1.25624200	-0.27814600	H	-0.92153400	-0.72773600	1.63252100
C	-1.88510900	-1.95410500	0.17807400	H	-1.67779600	0.87211600	2.88837300
C	-1.84712800	-0.91761600	1.09586300	H	-3.28333500	1.58865800	2.62908900
N	-2.87237900	-0.12190100	1.48005600	H	-3.11432900	0.09381400	3.58777400
C	-4.12245200	-0.06369800	0.79135300	H	-3.23912700	0.19382200	-1.16366900
C	-2.73459600	0.64919300	2.72161300	H	-5.39112100	0.36760900	-2.33472200
C	-4.16332800	0.11615900	-0.60049600	H	-7.46042200	-0.07474400	1.42566600
C	-5.38116400	0.21804800	-1.25828600	H	-5.31475200	-0.26674300	2.57970000
C	-6.59309900	0.15243800	-0.54675900	H	-10.34659700	0.37844000	-0.79525100
C	-6.54842600	-0.01724200	0.84227300	H	-9.43791900	-0.69284100	0.29243600
C	-5.32416300	-0.12406500	1.50352000	H	-9.31613500	1.09077200	0.46568400

Supplementary Table 13. Cartesian coordinates of optimized SDA-TS (B’-C) in Gaussian package.

C	-3.05626700	0.79615300	-0.32299000
C	-1.70300200	-0.92948600	-0.28527300
C	-1.70154100	0.47627600	-0.45367300
C	-0.55637100	1.32165200	-0.68252500
O	-3.68296000	1.94841600	-0.41821900
C	-0.63179100	2.71977000	-0.88745400
C	0.67743000	3.36125100	-0.90263100
C	1.61802100	2.83417600	-0.08323500
C	1.17923300	1.89178600	0.91093700
O	-1.70308400	3.39634400	-0.99323800
H	0.28155100	2.11818800	1.46938000
H	2.67091900	3.09651100	-0.13297700
H	0.84612700	4.24232100	-1.51105500
H	-2.96939600	2.63833600	-0.69096400
H	0.33862300	0.81576000	-1.00911200
C	-0.49021600	-1.79044500	-0.30667500
N	-2.89055400	-1.43952600	-0.06704000
N	-3.72603400	-0.36896900	-0.09309800
C	-5.12718600	-0.56548700	0.06030900
C	-5.92902400	0.43363200	0.60585600
C	-5.67516200	-1.78465100	-0.33094900
C	-7.29287000	0.20465900	0.74523600
H	-5.49453400	1.37336200	0.91537500
C	-7.03687900	-2.00113000	-0.17394200
H	-5.03128100	-2.54857900	-0.74567000
C	-7.85197700	-1.00774400	0.35924400
H	-7.91801700	0.98273200	1.16736100
H	-7.46192500	-2.95066300	-0.47753900
F	-0.75237600	-3.06781100	-0.05655400
F	0.14320000	-1.72765500	-1.49153000
F	0.41244500	-1.37370900	0.61023600
N	1.94872500	0.95154200	1.45703700
C	3.15567000	0.48925400	0.85282500
C	4.27083700	0.24920400	1.64716200
C	3.22321200	0.23106900	-0.51812600
C	5.45098100	-0.22429100	1.08443800
H	4.22942400	0.43865900	2.71381400
C	4.39829100	-0.23419700	-1.07785700
H	2.35669700	0.37271000	-1.15149300
C	5.52981200	-0.46460100	-0.28608600
H	6.30073200	-0.39610200	1.73228900
H	4.43299600	-0.43709300	-2.14264500
S	6.97536000	-1.05910600	-1.11389600
C	8.15926200	-1.22312700	0.23943000
H	7.82417900	-1.95284200	0.97571600
H	9.08011900	-1.58508400	-0.21569000
H	8.35881900	-0.26235300	0.71267700
H	-8.91576200	-1.17889600	0.47435100
C	1.52014300	0.30454800	2.70029200
H	1.81247400	-0.74295300	2.68062400

H	1.96720000	0.79212600	3.56895900	H	0.43562700	0.36309600	2.77704100

Supplementary Table 14. Cartesian coordinates of optimized SDA-C in Gaussian package.

C	1.14694100	-0.17431600	-0.29133000	C	-6.65169000	2.18344400	-0.68112200
C	1.38589100	1.21748800	-0.34491400	N	-1.41469700	-1.26683500	1.78517100
N	2.65919300	1.53900600	-0.23107200	C	-0.74737400	-0.30897600	2.66034100
N	3.29495200	0.35612900	-0.12237600	C	0.37766700	2.31257700	-0.45346200
C	2.41768700	-0.68303000	-0.14142700	F	-0.18016800	2.59586200	0.74340100
O	2.75741900	-1.97435400	-0.03077600	F	-0.63399500	1.97502900	-1.26598400
C	4.70819800	0.29334300	0.01919200	F	0.90799900	3.44494400	-0.90735200
C	5.34504400	1.18338400	0.87888100	H	3.65706400	-2.07603900	0.31019100
C	6.72410000	1.11435300	1.01889100	H	4.75603700	1.91953300	1.41215200
C	7.45684800	0.15563500	0.32238400	H	7.22835800	1.80843400	1.68115900
C	6.80998700	-0.72384300	-0.53769000	H	8.53267100	0.10356900	0.44224200
C	5.43017100	-0.65332300	-0.70415300	H	7.37796400	-1.45528400	-1.10059200
C	-0.09877500	-0.98559800	-0.40362700	H	4.92737900	-1.29076800	-1.42384300
C	-0.04207400	-1.99261300	-1.56881300	H	-0.95041600	-0.33283200	-0.62464400
C	-0.72118600	-3.22561100	-1.10981900	H	-0.91379400	-4.06523300	-1.76522000
C	-0.97276600	-3.14441200	0.19649900	H	-1.42455100	-3.91898200	0.80610100
C	-0.48364200	-1.86056900	0.82332000	H	0.42378000	-2.10166500	1.38865700
O	0.48703100	-1.80562900	-2.63215300	H	-3.45019100	-2.81206900	1.07560700
C	-2.65297800	-0.82094700	1.23507800	H	-5.57515400	-2.11909300	0.07270900
C	-3.63027300	-1.76127300	0.88388900	H	-4.34592600	1.99077900	0.36219200
C	-4.83652100	-1.36742600	0.33245600	H	-2.21985100	1.29017300	1.29466000
C	-5.12121200	-0.01194600	0.12999600	H	-7.60334900	2.48112500	-1.12082600
C	-4.16651300	0.93155600	0.49757300	H	-6.56420600	2.64172800	0.30375500
C	-2.94560000	0.52840700	1.03551400	H	-5.84575100	2.51955500	-1.33313900
S	-6.70392700	0.38052200	-0.57129800	H	-1.46220700	0.07132000	3.39091900

H	0.04886200	-0.82504100	3.20034300
H	-0.30174100	0.54384900	2.13320000

Supplementary Table 15. Cartesian coordinates of optimized SSDA-A in Gaussian package.

N	3.41723200	-0.13423800	0.20201200
C	2.20546300	-0.73627900	0.23497100
C	0.98226200	-0.12800300	0.12603400
C	-0.19277000	-0.89603500	0.22040700
C	-1.46654300	-0.36357200	0.10516300
C	-2.55596600	-1.24696600	0.20323300
C	-3.91479800	-0.98916400	0.12058000
O	-1.56714400	0.96438600	-0.09670400
C	4.60559700	-0.91177200	0.09074600
C	4.19239500	1.96121100	-0.78419900
C	3.53738600	1.29333000	0.24151700
C	4.33473600	3.34380800	-0.73989600
C	3.81491600	4.06950200	0.33269600
C	3.15541100	3.38550000	1.36248900
C	3.02219000	2.00893300	1.32162800
C	-4.98017800	-1.94459600	0.21863600
N	-6.15506400	-1.42012100	0.10466800
N	-5.96068600	-0.07518800	-0.07303700
C	-4.61639200	0.27261100	-0.08379500
O	-4.17587500	1.41371900	-0.23954300
C	-7.09117200	0.75999400	-0.26499500
C	-6.98649400	2.14835800	-0.16201800
C	-8.11996400	2.93069400	-0.35499000
C	-9.34996500	2.35067000	-0.64099500
C	-9.44304400	0.96563200	-0.73505900
C	-8.32270100	0.16727200	-0.55199700
S	3.93295000	5.82795200	0.49675200
C	4.79955700	6.31300900	-1.01279500
C	5.75996200	-0.52621700	0.77920400
C	6.90865400	-1.28875100	0.68602800
C	6.93782000	-2.45469100	-0.09115400
C	5.78806500	-2.82861000	-0.78366900
C	4.63396800	-2.05498700	-0.70151500
S	8.46494700	-3.35040200	-0.12818200
C	8.07153200	-4.75953900	-1.18846500
C	-4.83667900	-3.41587600	0.43201000
F	-4.11115000	-3.97936300	-0.54990100
F	-6.00780400	-4.03979600	0.47944400
F	-4.18433000	-3.67826200	1.57765400
H	2.25195600	-1.81233900	0.37410300
H	0.89657600	0.93919100	-0.03804800
H	-0.10855300	-1.96502600	0.38895600
H	-2.27455800	-2.28284600	0.36764500
H	-2.50427300	1.25438800	-0.17122500
H	4.59565300	1.39912800	-1.61969100
H	4.84820100	3.83949700	-1.55358100
H	2.74957900	3.93634100	2.20433700
H	2.51649800	1.48294700	2.12389900
H	-6.03458000	2.60610200	0.06037400
H	-8.03222500	4.00846100	-0.27442800

H	-10.22731900	2.97001800	-0.78794400
H	-10.39530300	0.49661700	-0.95635500
H	-8.39179500	-0.90969600	-0.62277600
H	4.88372200	7.39845100	-0.96723200
H	5.80202300	5.88764100	-1.05342200
H	4.22960400	6.04515900	-1.90206700
H	5.75049700	0.36714600	1.39316900
H	7.79508500	-0.98194600	1.23129700
H	5.77605900	-3.71069400	-1.41127500
H	3.76027700	-2.33575900	-1.27929800
H	8.97972700	-5.36015100	-1.22916100
H	7.27169600	-5.36584100	-0.76387700
H	7.81894700	-4.44159200	-2.19961400

Supplementary Table 16. Cartesian coordinates of optimized SSDA-TS (A-B) in Gaussian package.

C	-4.55181700	-0.43879500	-0.84769500	C	-9.50568900	1.10996100	-1.31177600
C	-4.42787200	-1.45277000	1.12638300	H	-8.45830800	1.96621600	-2.98066000
C	-3.64682300	-1.10894500	0.00845700	H	-10.24288700	0.12915200	0.45529800
C	-2.23775600	-1.38046100	-0.20953100	F	-4.89409400	-2.38444500	3.23930800
O	-4.37796600	0.06734900	-1.99602800	F	-3.42709400	-3.38289300	2.01199700
C	-1.47925400	-0.96031500	-1.23169100	F	-2.94378200	-1.50958400	2.94460100
C	-0.03812400	-1.30591200	-1.29667300	C	4.62660800	-0.71520500	-0.54017900
C	0.91100500	-0.47583200	-0.82678100	C	4.81131400	-1.97395900	0.01286600
C	2.27796300	-0.86727100	-0.93030700	C	6.96774100	-0.55350800	-1.01311400
O	-1.83104200	-0.19175100	-2.29392900	C	6.08206400	-2.53546100	0.04132500
H	2.50829400	-1.85906700	-1.30853500	H	3.97514200	-2.50685300	0.45222400
H	0.64249800	0.46458200	-0.35954700	C	7.17241400	-1.83306900	-0.47386400
H	0.25493900	-2.23664200	-1.78344500	H	7.80641800	0.00378700	-1.41525900
H	-2.82936800	-0.03774000	-2.27716100	H	6.20611700	-3.51393100	0.48597600
H	-1.73655200	-1.98832900	0.53808200	N	3.30720400	-0.14036100	-0.58501100
C	-3.94337100	-2.17672300	2.33005400	C	5.70724500	0.00830400	-1.04263900
N	-5.68082800	-1.07044800	1.05069100	H	5.55694500	0.99639900	-1.46194400
N	-5.76539500	-0.44184800	-0.15807900	S	8.82732100	-2.43930200	-0.48010200
C	-7.01105600	0.08631800	-0.56618200	C	8.64699100	-4.08577500	0.23947800
C	-7.13167300	0.86405700	-1.72117800	H	7.99892800	-4.71526200	-0.36894400
C	-8.14212700	-0.17295500	0.21423100	H	9.64754700	-4.51595400	0.24182800
C	-8.37759600	1.36513600	-2.08164000	H	8.28680200	-4.03531400	1.26620200
H	-6.25926300	1.06563800	-2.32251000	C	3.17683000	1.25400800	-0.23343000
C	-9.37552600	0.33924200	-0.16072300	C	3.68832000	1.70017700	0.97631400
H	-8.03826700	-0.77194200	1.10771400	C	2.57188900	2.13520300	-1.12553700

C	3.57156000	3.04073300	1.31560300
H	4.16871100	1.00449900	1.65524000
C	2.46158800	3.47050400	-0.78586200
H	2.19074100	1.77886200	-2.07627800
C	2.95548000	3.93905100	0.43931900
H	3.96462800	3.36998200	2.26812400
H	1.98640300	4.15727800	-1.47685600
S	2.75911700	5.65987400	0.76270200
C	3.39679400	5.82350500	2.44430800
H	3.25682600	6.87064600	2.70893900
H	2.83026100	5.20881100	3.14262300
H	4.45950800	5.59080400	2.49578000
H	-10.47186900	1.50589100	-1.60241200

Supplementary Table 17. Cartesian coordinates of optimized SSDA-B in Gaussian package.

C	-0.14196300	-2.32028200	-0.21132400
C	1.24313100	-2.28319200	-0.15294300
C	1.91208100	-1.05263200	-0.00989600
C	3.26383600	-0.76900600	0.01458500
O	1.87070000	-3.47194800	-0.29398500
C	3.85782900	0.53403800	0.12136900
N	5.14864700	0.51196700	0.11987100
N	5.51353100	-0.80475200	0.00910100
C	4.42002000	-1.65880300	-0.05534400
O	4.47393500	-2.88510100	-0.14394600
C	6.89289600	-1.13706700	0.02678300
C	7.33342600	-2.39704200	-0.38171800
C	8.69397200	-2.68307300	-0.35005200
C	9.61509400	-1.73286900	0.07400000
C	9.16428400	-0.47832700	0.47249500
C	7.81005100	-0.17509900	0.45469500
C	-1.05165700	-1.25608200	-0.04208900
C	-2.39200300	-1.44501000	-0.23427000
N	-3.34598600	-0.47845500	-0.18192900
C	-4.72477100	-0.82380200	-0.13431100
C	-2.97250000	0.90226300	-0.10432000
C	-5.66859100	-0.02044000	-0.78229300
C	-7.00730300	-0.36221900	-0.75303800
C	-7.44192000	-1.51353000	-0.08242300
C	-6.49939100	-2.30490200	0.57037000
C	-5.15243200	-1.95512300	0.55421100
C	-3.39987800	1.68304400	0.96790200
C	-2.99340900	3.00352300	1.06523700
C	-2.14782200	3.56330100	0.09989900
C	-1.74264300	2.78046600	-0.98296700
C	-2.16154500	1.45977000	-1.08623500
S	-9.18031500	-1.85041300	-0.11821600
C	-9.29983400	-3.41321800	0.78095800
S	-1.66323900	5.24955800	0.33516600
C	-0.34262100	5.45199000	-0.88401800
C	3.13031100	1.83577900	0.22128200
F	2.31613100	1.85693300	1.28945800
F	2.34152800	2.03665400	-0.85235900
F	3.95319700	2.87293400	0.31591100
H	-0.55756100	-3.30442000	-0.42129100
H	1.26186100	-0.18928700	0.06872300
H	2.84611900	-3.38035200	-0.24406300
H	6.62376000	-3.14004900	-0.71265200
H	9.03078300	-3.66368700	-0.66732900
H	10.67346900	-1.96607300	0.09322400
H	9.87007900	0.27442500	0.80544900
H	7.45568100	0.79902400	0.76344100
H	-0.70101000	-0.26909700	0.23029600
H	-2.76925100	-2.43478600	-0.47202300
H	-5.34783800	0.86666200	-1.31647200
H	-7.72693500	0.26688400	-1.26660200
H	-6.79602400	-3.19033500	1.11800700

H	-4.43935000	-2.55868500	1.10476400
H	-4.03715800	1.24842400	1.73036100
H	-3.31459700	3.59979900	1.91263500
H	-1.10176400	3.18492900	-1.75594400
H	-1.84325900	0.85285200	-1.92703500
H	-10.35510300	-3.68408500	0.76166100
H	-8.72879800	-4.19987200	0.28818600
H	-8.98825100	-3.30372700	1.81943500
H	0.08013900	6.43935900	-0.69994900
H	0.43804000	4.70468700	-0.74220100
H	-0.72417100	5.42273800	-1.90402100

Supplementary Table 18. Cartesian coordinates of optimized SSDA-TS (B-B') in Gaussian package.

C	-4.39774200	-1.02589400	0.76633000	C	-9.28978600	-1.13837700	-0.91551000
C	-3.27891800	0.32935300	-0.69770600	H	-9.40580300	-1.64187400	1.17051200
C	-3.05207000	-0.48407200	0.47891100	H	-8.85277800	-0.57366200	-2.94440300
C	-1.86720300	-0.66368400	1.11390700	F	-2.75149300	1.76925900	-2.47309600
O	-4.76325900	-1.79056400	1.64752100	F	-1.65099200	2.00605400	-0.63009700
C	-1.53791000	-1.44582200	2.28396200	F	-1.27252900	0.30680700	-1.89290900
C	-0.25666300	-1.44655300	2.72068500	C	3.84401300	-1.22353500	0.09157300
C	0.84992500	-0.67474300	2.12011500	C	4.25140300	-2.42820300	0.67316400
C	1.71113600	-1.21899400	1.25317100	C	5.65978400	-1.40755900	-1.49089700
O	-2.45601100	-2.17389700	2.96351400	C	5.34167600	-3.12044700	0.16110900
H	1.63422200	-2.26960900	0.98854700	H	3.73621600	-2.81304900	1.54509000
H	0.94856300	0.37202000	2.39911600	C	6.05942600	-2.61884700	-0.92101800
H	-0.06140000	-2.04009900	3.61203900	H	6.20107200	-1.01370000	-2.34396600
H	-3.34594800	-2.12518800	2.56245600	H	5.64743200	-4.05345400	0.62056200
H	-1.00909000	-0.14297700	0.69775600	N	2.71747300	-0.53567100	0.57574600
C	-2.24627200	1.11427100	-1.43994100	C	2.65314500	0.88624600	0.47995100
N	-4.50262500	0.32482000	-1.08571700	C	3.65579000	1.68075500	1.01817700
N	-5.20200000	-0.47475200	-0.21342400	C	1.56761500	1.48959800	-0.15797400
C	-6.58762700	-0.70430000	-0.43808800	C	3.58649500	3.06786800	0.91998300
C	-7.41444700	-1.10723200	0.60869900	H	4.50103800	1.21299400	1.51144900
C	-7.10280100	-0.51134800	-1.71847800	C	1.48425500	2.86713600	-0.24026800
C	-8.76356400	-1.32662200	0.35663300	H	0.79304600	0.86779100	-0.59384400
H	-7.01277700	-1.24955000	1.60100200	C	2.49634400	3.67363300	0.29766100
C	-8.45442100	-0.72603200	-1.94823900	H	0.63176500	3.32266200	-0.73252400
H	-6.44909800	-0.19038100	-2.51825800	S	7.46531100	-3.50610800	-1.56756900

C	8.78774400	-2.61572800	-0.69022300
H	9.73487800	-3.06073300	-0.99476800
H	8.78635100	-1.56078700	-0.96146200
H	8.67111700	-2.72296900	0.38727200
C	4.56885700	-0.71453400	-0.99481400
H	4.25991800	0.21456100	-1.45925100
H	4.38736200	3.65925600	1.34459200
S	2.29083600	5.42436500	0.12719000
C	3.74760500	6.07524800	0.97294000
H	3.66440900	7.15963200	0.91277900
H	3.76110400	5.78427700	2.02270600
H	4.66805300	5.76859000	0.47742500
H	-10.34338200	-1.30953700	-1.10122200

Supplementary Table 19. Cartesian coordinates of optimized SSDA-B’ in Gaussian package.

C	-0.25094900	-3.05616900	0.67341200
C	1.11185300	-2.71456400	0.66586600
C	1.56992700	-1.55143300	0.01430900
C	2.86906700	-1.05853400	-0.13973700
O	1.92730900	-3.63981100	1.22072300
C	3.27263000	0.12298400	-0.85107000
N	4.56313600	0.33344800	-0.81782000
N	5.10497500	-0.70908800	-0.09901900
C	4.13168400	-1.60335200	0.35091800
O	4.35712400	-2.64131500	1.00696900
C	6.51705700	-0.76653200	0.06050600
C	7.11230800	-1.68492100	0.93793200
C	8.50136500	-1.70561300	1.06830300
C	9.30507000	-0.82500600	0.34402300
C	8.70287000	0.08802200	-0.52419200
C	7.31826200	0.12206400	-0.67221000
C	-1.41380700	-2.28297400	0.45540800
C	-1.52343400	-0.91632400	0.62160700
N	-2.64231500	-0.16383200	0.41717200
C	-2.58146300	1.26235600	0.51225100
C	-3.90220700	-0.77260200	0.07234000
C	-3.65133700	1.98197400	1.06679500
C	-3.58187900	3.36426500	1.17569500
C	-2.44428100	4.06848900	0.74121400
C	-1.38454800	3.34692000	0.17853600
C	-1.45919200	1.96025600	0.05454500
C	-4.44787300	-0.57704300	-1.20153100
C	-5.67044300	-1.14988700	-1.52982600
C	-6.37098800	-1.93124700	-0.59305700
C	-5.81870100	-2.12177200	0.68098900
C	-4.59264800	-1.54208300	1.01047100
S	-2.46840000	5.83957700	0.93705900
C	-0.82074300	6.36171600	0.35624200
S	-7.92736400	-2.61341300	-1.12752300
C	-8.50446800	-3.56883700	0.31421200
C	2.39747000	1.15877900	-1.48278900
F	1.78879100	1.93523900	-0.54242700
F	1.39247400	0.59522300	-2.20363800
F	3.07456200	1.97854500	-2.29763200
H	-0.42538500	-4.10864600	0.89645100
H	0.79621600	-0.99621300	-0.50109300
H	2.88258200	-3.35429200	1.16461800
H	6.49518400	-2.37113900	1.50050000
H	8.95320700	-2.42159600	1.74999900
H	10.38578600	-0.84896400	0.45400800
H	9.31269400	0.78173100	-1.09724500
H	6.85114300	0.83010400	-1.34536000
H	-2.31402300	-2.84366100	0.22296100
H	-0.68146600	-0.34909500	1.00068700
H	-4.53228700	1.45856600	1.42314100
H	-4.41747800	3.90242400	1.61565300
H	-0.49462300	3.84621900	-0.18733700

H	-0.64234000	1.43472400	-0.42576800
H	-3.91242600	0.02202400	-1.93194900
H	-6.08256400	-0.99456800	-2.52329500
H	-6.33433400	-2.71209700	1.42991100
H	-4.17360800	-1.68642200	2.00209200
H	-0.78983600	7.44551300	0.49922300
H	-0.02274800	5.90619700	0.94920600
H	-0.67745200	6.14410100	-0.70572100
H	-9.45414600	-4.01575400	0.00678300
H	-7.80740500	-4.37100300	0.57199100
H	-8.68455200	-2.92611300	1.18038300

Supplementary Table 20. Cartesian coordinates of optimized SSDA-TS (B'-C) in Gaussian package.

C	-3.82052400	-1.27333700	-0.70679500
C	-2.47263400	-0.61254600	0.99238500
C	-2.43150400	-1.26822700	-0.27939300
C	-1.29126000	-1.71345100	-0.91824400
O	-4.33547000	-1.74366200	-1.72456100
C	-1.13286700	-2.36554800	-2.15981700
C	0.14806300	-2.61387700	-2.63621300
C	1.30734900	-1.92993100	-2.21711700
C	1.24831100	-0.63450300	-1.75870200
O	-2.16909700	-2.87211900	-2.87351900
H	0.35214400	-0.04561500	-1.93229400
H	2.27167500	-2.42225800	-2.29412300
H	0.23814500	-3.38709600	-3.39440000
H	-3.03298500	-2.57889600	-2.51169700
H	-0.37277100	-1.60778100	-0.35491300
C	-1.28290300	-0.30545100	1.83912400
N	-3.65911600	-0.23732200	1.34086500
N	-4.49529500	-0.63368400	0.32558100
C	-5.89058100	-0.41014900	0.45147600
C	-6.71997300	-0.43670200	-0.66980400
C	-6.42150400	-0.15464800	1.71591400
C	-8.08283700	-0.21461100	-0.50980500
H	-6.30639500	-0.63148500	-1.64781100
C	-7.78331100	0.07302000	1.85393300
H	-5.76595500	-0.13112100	2.57561100
C	-8.62231900	0.04090500	0.74493700
H	-8.72419100	-0.23745800	-1.38326500
H	-8.18910000	0.27184300	2.83911500
F	-1.58898400	0.36905800	2.93823900
F	-0.64740300	-1.43241500	2.20976300
F	-0.37696700	0.41724500	1.14944300
C	2.18086000	1.44481800	-0.98594900
C	1.67179800	2.22641100	-2.02000300
C	2.52993100	3.42535800	0.33542500
C	1.55998500	3.60217200	-1.85866000
H	1.37083200	1.76461100	-2.95340600
C	1.98965800	4.21389600	-0.68416000
H	2.88473500	3.88834000	1.24923300
H	1.14347700	4.20407100	-2.65759300
N	2.24172400	0.03038000	-1.12296100
C	2.61392100	2.05079100	0.19598500
H	3.01176900	1.44461700	1.00134800
C	3.30853600	-0.68361300	-0.48991300
C	4.62682500	-0.39791600	-0.81331400
C	3.01910900	-1.64030300	0.48302100
C	5.66360500	-1.07198900	-0.17674700
H	4.84524400	0.35632800	-1.56123600
C	4.04867900	-2.31596600	1.11141200
H	1.98706700	-1.83791600	0.75396300
C	5.38414100	-2.04039700	0.78733000

H	6.68313200	-0.83216800	-0.44841200
H	3.81794800	-3.05471400	1.87116000
S	1.88945900	5.98585400	-0.53079700
S	6.61897400	-2.96223200	1.65558800
C	0.93332800	6.11101000	1.01017000
H	0.71408400	7.16819700	1.15707400
H	-0.00084400	5.55935500	0.92184800
H	1.50412700	5.75129100	1.86442500
C	8.15467000	-2.28753700	0.98661200
H	8.24742000	-1.22307600	1.19953200
H	8.95711300	-2.81681300	1.49858400
H	8.24037400	-2.47562900	-0.08303600
H	-9.68576900	0.21499200	0.85833700

Supplementary Table 21. Cartesian coordinates of optimized SSDA-C in Gaussian package.

C	-0.49424000	-1.80130800	-0.10178700
C	-0.72698500	-1.82570700	1.28890300
N	-1.93751000	-1.43515800	1.62714100
N	-2.54473700	-1.16444800	0.46071200
C	-1.70080400	-1.35730900	-0.59597000
O	-2.05661200	-1.11330000	-1.87019200
C	-3.87069900	-0.64456700	0.45318600
C	-4.29449600	0.10866900	1.54521600
C	-5.58523500	0.61982700	1.55840900
C	-6.44393200	0.39481800	0.48637800
C	-6.01110500	-0.36416800	-0.59459100
C	-4.72684200	-0.89784100	-0.61625100
C	0.80245800	-2.00278500	-0.80889200
C	1.06552400	-3.37802300	-1.44330900
C	1.97343400	-3.13124300	-2.59523000
C	2.03824700	-1.82259900	-2.85362500
C	1.09687200	-1.03022900	-1.97835000
O	0.61892300	-4.43024400	-1.07455600
C	0.32603000	1.13846600	-1.30089200
C	-0.50487700	1.58659700	-2.32625800
C	-1.69996200	2.23401100	-2.03588800
C	-2.08199900	2.45888000	-0.70710800
C	-1.23098900	2.03776000	0.32021900
C	-0.03993900	1.38648700	0.02127100
S	-3.63453000	3.26875600	-0.44701700
C	-3.59113600	3.64999700	1.31814200
N	1.46881000	0.33407500	-1.62224500
C	2.70560900	0.65489700	-1.04533500
C	3.00088900	2.00030000	-0.76038500
C	4.22564400	2.36274800	-0.22802100
C	5.20523800	1.40350400	0.04020400
C	4.92385400	0.07300400	-0.25277600
C	3.69408800	-0.30389900	-0.78125100
S	6.78324700	1.87508900	0.72590700
C	6.37645700	1.77826300	2.49836500
C	0.27736500	-2.15720000	2.33952000
F	1.23612300	-1.20757000	2.40356000
F	0.91024000	-3.30650500	2.07128800
F	-0.26300700	-2.25383600	3.55058600
H	-2.34342900	-0.19000400	-1.96698800
H	-3.61714500	0.26289500	2.37608100
H	-5.91976700	1.19934000	2.41179200
H	-7.44854400	0.80118500	0.49839700
H	-6.67994600	-0.56162400	-1.42432400
H	-4.40692000	-1.52473300	-1.43829800
H	1.61476900	-1.87656000	-0.08191700
H	2.45902100	-3.93019600	-3.14058400
H	2.61565900	-1.34913000	-3.64025300
H	0.17712900	-0.93089400	-2.55966100
H	-0.21436000	1.41361200	-3.35751700
H	-2.33007000	2.58647900	-2.84642100
H	-1.49148100	2.19469900	1.35971300

H	0.59651600	1.03376800	0.82665900
H	-4.50524500	4.20772800	1.52196700
H	-3.59272700	2.74679300	1.92614900
H	-2.73380700	4.27746700	1.56185200
H	2.26584500	2.76661200	-0.97595100
H	4.43195200	3.40866400	-0.02776000
H	5.67249900	-0.68788500	-0.06069900
H	3.52720100	-1.35167000	-0.98830500
H	7.27673000	2.04939200	3.05075600
H	5.57948300	2.47744600	2.74949100
H	6.08169500	0.76525700	2.77004900

Supplementary Table 22. Cartesian coordinates of optimized SDAS-A in Gaussian package.

N	-5.76564300	1.91979600	-0.19188200	S	-7.47271000	-3.82356100	-0.14050500
C	-4.48750700	2.31554100	-0.14913000	C	-6.67756500	-4.55065300	1.31075600
C	-3.36018300	1.52276500	-0.15876500	S	7.69518700	-3.84624400	-0.16816700
C	-2.09465500	2.11472600	-0.07789600	C	9.25975700	-2.95404000	-0.31506400
C	-0.90634100	1.39148700	-0.07744500	C	2.86641400	3.91543500	0.27039300
C	0.29201500	2.10649600	0.01922600	F	2.26505200	4.51271200	-0.77409500
C	1.60321300	1.64130300	0.04847900	F	4.11623400	4.36207400	0.32697800
O	-1.00611300	0.04955000	-0.16085500	F	2.22582100	4.35211200	1.36919500
C	-6.13913500	0.53997600	-0.16768900	H	-4.36207100	3.39460900	-0.10761000
C	-6.84248900	2.89725000	-0.32812200	H	-3.42981300	0.44495600	-0.23259500
C	2.79341700	2.42866900	0.15588300	H	-2.02031500	3.19547700	-0.00747700
N	3.88045200	1.72731300	0.15573900	H	0.16404100	3.18283800	0.08291100
N	3.48842200	0.41915400	0.04802900	H	-0.12044100	-0.38110700	-0.16638400
C	2.10811500	0.28033500	-0.02645300	H	-6.46191400	3.88748700	-0.08350500
O	1.50369100	-0.79238300	-0.13542800	H	-7.23217700	2.91384300	-1.34852000
C	4.48425500	-0.58798200	-0.00488200	H	-7.65144300	2.64501100	0.35746400
C	4.15305300	-1.94597400	0.03424800	H	3.12030500	-2.25180600	0.10144100
C	5.15785100	-2.89865600	-0.01993700	H	4.88504100	-3.94865300	0.00929300
C	6.50299300	-2.53240700	-0.10836200	H	7.85113400	-0.84506200	-0.20692000
C	6.82250100	-1.17625200	-0.14134500	H	6.08234100	0.83719900	-0.11902300
C	5.82316100	-0.21237600	-0.09248900	H	-7.22506700	0.63496100	-2.02499500
C	-6.92733800	0.01166900	-1.18918200	H	-7.92095600	-1.72159400	-1.95161100
C	-7.31500300	-1.31692100	-1.14782700	H	-5.81349400	-2.21292100	1.76839200
C	-6.92013000	-2.14286000	-0.08900700	H	-5.14813000	0.14345600	1.69344700
C	-6.13320100	-1.60608700	0.93120400	H	-6.95638000	-5.60400500	1.30142700
C	-5.75157100	-0.27045000	0.89302900	H	-5.59229400	-4.47646800	1.24638700

H	-7.04134300	-4.10263400	2.23497000
H	10.03339300	-3.71974700	-0.36875300
H	9.29487100	-2.35844300	-1.22704900
H	9.44811500	-2.32975600	0.55808900

Supplementary Table 23. Cartesian coordinates of optimized SDAS-TS (A-B) in Gaussian package.

C	-2.19662400	-0.54100400	-0.68785000
C	-2.00251000	-1.88078100	1.07499800
C	-1.31602000	-1.46511300	-0.08012400
C	-0.00756200	-1.89076600	-0.54262100
O	-2.08181400	0.12264500	-1.75965000
C	0.70619000	-1.35852600	-1.54435700
C	2.04219300	-1.90003900	-1.89554400
C	3.17517700	-1.35911700	-1.41580900
C	4.42629800	-1.91749600	-1.82168300
O	0.39411600	-0.30764800	-2.34551700
H	4.41400500	-2.82042000	-2.42602700
H	3.14394400	-0.52114000	-0.72916200
H	2.09792700	-2.72880900	-2.60180700
H	-0.57116200	-0.05444300	-2.19916400
H	0.44621300	-2.73073900	-0.02478900
C	-1.50241900	-2.86191100	2.07324900
N	-3.17680600	-1.32009900	1.24070900
N	-3.30514800	-0.49113500	0.16239200
C	-4.47223600	0.28929600	0.03977900
C	-4.70436600	1.09157400	-1.08181400
C	-5.41647700	0.25720800	1.07176300
C	-5.86999300	1.84336300	-1.15835900
H	-3.97707100	1.12252700	-1.87806200
C	-6.57403800	1.01263600	0.97919000
H	-5.22963500	-0.36049900	1.93833200
C	-6.81695600	1.81177900	-0.13789000
H	-6.04275500	2.46642300	-2.02883800
H	-7.29673500	0.98611000	1.78717000
F	-2.36472600	-3.09210700	3.06147700
F	-1.22289500	-4.05290400	1.50277500
F	-0.34787900	-2.45322800	2.63830900
N	5.60041700	-1.45703100	-1.51558700
C	5.78175900	-0.20729200	-0.81476300
C	6.48484700	-0.18445100	0.37967200
C	5.27951100	0.96715300	-1.37031800
C	6.67945600	1.02237900	1.03905100
H	6.86552300	-1.10113000	0.81527500
C	5.47712300	2.16623700	-0.71248500
H	4.73743600	0.94106200	-2.30928300
C	6.17996500	2.20983500	0.49966700
H	7.22044100	1.01747100	1.97567300
H	5.08176400	3.07931100	-1.14255800
S	6.37543500	3.79567400	1.24345200
C	7.26474200	3.41238900	2.76803700
H	7.39711100	4.36634700	3.27633300
H	6.68330700	2.75334500	3.41150000
H	8.24677300	2.98816500	2.56261600
S	-8.31609100	2.77325600	-0.25709600
C	-9.38603500	1.52584800	-1.03875800
H	-9.48474400	0.65080500	-0.39766400

H	-10.36715000	1.97947500	-1.18012700
H	-8.98396300	1.23097000	-2.00712000
C	6.82906600	-2.15397300	-1.91930800
H	7.43818800	-1.47450600	-2.51313800
H	7.38299900	-2.44885400	-1.02912900
H	6.57337400	-3.03768900	-2.49838000

Supplementary Table 24. Cartesian coordinates of optimized SDAS-B in Gaussian package.

C	1.84810800	3.21771000	-0.06483900	C	5.13912100	-1.01547200	-1.02796000
C	0.54536800	2.72995800	-0.00391200	C	5.02675600	0.36725000	-1.07197600
C	0.30915400	1.35259300	0.10026300	S	6.06521500	-3.38377600	0.13811900
C	-0.88542500	0.64824300	0.12564400	C	4.78568500	-4.00831600	-0.97837000
O	-0.42910500	3.66625600	-0.07841700	C	0.07050400	-1.77703400	0.33045000
C	-1.03118300	-0.77477100	0.21464000	F	0.80453900	-1.57082500	1.43625100
N	-2.26194100	-1.16915800	0.20208700	F	0.92614300	-1.68365700	-0.70694700
N	-3.02693000	-0.03678300	0.10276600	F	-0.37899000	-3.02602100	0.36841900
C	-2.26112200	1.12150400	0.04845200	H	1.91381600	4.29605900	-0.20210500
O	-2.70452100	2.26832400	-0.04909900	H	1.20182200	0.73993200	0.15360000
C	-4.43719100	-0.17147800	0.04553000	H	-1.32369700	3.26224500	-0.07193600
C	-4.99160400	-1.45439000	-0.01229700	H	-4.34175400	-2.31861100	-0.01185800
C	-6.36370000	-1.61533000	-0.06615800	H	-6.77602200	-2.61821100	-0.10940200
C	-7.21907300	-0.50841700	-0.06733500	H	-7.28330600	1.64883500	-0.00680300
C	-6.65896800	0.76427900	-0.00965500	H	-4.86531100	1.93462900	0.09235700
C	-5.27865300	0.93869000	0.04822700	H	-10.75683600	0.67779900	-0.16522400
S	-8.96111900	-0.83790000	-0.14371200	H	-9.43275000	1.34905700	0.79954200
C	-9.67737800	0.82084800	-0.12171700	H	-9.36727300	1.40139800	-0.99038000
C	3.05310300	2.50232200	0.01416600	H	3.04975400	1.43273600	0.17577800
C	4.25975300	3.14594900	-0.11887200	H	4.27455900	4.22703900	-0.22725400
N	5.47727400	2.57364900	-0.13206500	H	6.46003900	4.39751900	-0.40300400
C	5.63454300	1.15540300	-0.09887400	H	7.02691000	3.47285200	0.99994600
C	6.67881000	3.39633200	-0.03356400	H	7.46796700	2.95833900	-0.64387700
C	6.39569900	0.55018100	0.89955100	H	6.87257400	1.15488000	1.66279500
C	6.51600400	-0.83041500	0.93921900	H	7.09071400	-1.29395500	1.73414100
C	5.87604000	-1.62984100	-0.01333500	H	4.64961800	-1.60227000	-1.79473300

H	4.45615200	0.83920900	-1.86432800
H	4.75580200	-5.08600500	-0.82026900
H	3.81117400	-3.58939700	-0.72714300
H	5.02937900	-3.81813900	-2.02291800

Supplementary Table 25. Cartesian coordinates of optimized SDAS-TS (B-B') in Gaussian package.

C	-2.31842800	1.05797900	-0.35500400	C	-7.16192100	-0.74515000	0.03529200
C	-0.98260800	-0.55276900	0.56796900	H	-7.19287600	0.66246700	-1.60376000
C	-0.90645600	0.75926500	-0.03923900	H	-6.76027600	-2.09729700	1.66563100
C	0.22394000	1.48137300	-0.23990300	F	-0.19977300	-2.50335900	1.61103000
O	-2.81683300	2.05245100	-0.86479200	F	1.05936800	-1.59448000	0.10989200
C	0.41574300	2.77480900	-0.85314700	F	0.83334800	-0.65337700	2.03014500
C	1.67856800	3.24800500	-0.97514200	N	4.53643100	2.08907500	1.23142400
C	2.89552000	2.54154500	-0.52489000	C	4.99965400	0.90032100	0.61466700
C	3.45885700	2.77018000	0.66699800	C	6.31321400	0.74047500	0.19240100
O	-0.61083400	3.51804200	-1.33376400	C	4.11379100	-0.17685300	0.46747200
H	3.06118800	3.54946800	1.31351300	C	6.74016600	-0.46305600	-0.37265900
H	3.32350100	1.79995000	-1.19560400	H	7.02400400	1.55213200	0.29547100
H	1.77908200	4.20370200	-1.48662200	C	4.52754900	-1.36131100	-0.10421600
H	-1.47864700	3.08872800	-1.19998400	H	3.09716100	-0.07711600	0.83070400
H	1.15306100	1.02708200	0.09170800	C	5.85338000	-1.52005600	-0.53627100
C	0.17260600	-1.34560500	1.08823800	H	3.82135200	-2.17931900	-0.20320200
N	-2.18010200	-1.01242100	0.62758400	S	-8.89374700	-1.10614600	0.12044600
N	-3.00407000	-0.06405400	0.06823000	C	-9.57574500	-0.05096900	-1.17668800
C	-4.40723900	-0.28574100	0.04658100	H	-9.19351700	-0.32799800	-2.15842700
C	-5.21567800	0.36686800	-0.87642300	H	-10.65167200	-0.21971500	-1.16102900
C	-4.96937900	-1.17523300	0.96252600	H	-9.38422900	1.00208100	-0.97348100
C	-6.58794000	0.13925400	-0.87483500	H	7.77297100	-0.54722200	-0.68597500
H	-4.78688800	1.05291400	-1.59234000	S	6.27885500	-3.08642500	-1.25054400
C	-6.33269100	-1.40325000	0.95017200	C	8.03101300	-2.87383400	-1.63096500
H	-4.33654800	-1.68797800	1.67460700	H	8.35669500	-3.81434900	-2.07332200

H	8.18724000	-2.07513500	-2.35532300
H	8.61459600	-2.69155600	-0.72904300
C	5.51310900	2.92802300	1.91051200
H	6.12956400	3.51090200	1.21621900
H	4.98554400	3.62572700	2.56196300
H	6.16184900	2.30684000	2.52739000

Supplementary Table 26. Cartesian coordinates of optimized SDAS-B’ in Gaussian package.

C	-1.78755400	-2.69639300	-0.27547400
C	-0.42645600	-2.34081100	-0.27564600
C	-0.03209500	-0.98865000	-0.22639300
C	1.24879400	-0.42876800	-0.19668000
O	0.43456800	-3.37105800	-0.43349700
C	1.57520500	0.96779500	-0.17664100
N	2.86117400	1.20068300	-0.11748800
N	3.47198700	-0.03617200	-0.11618800
C	2.54968100	-1.08342200	-0.16284300
O	2.83792400	-2.29940500	-0.18842700
C	4.89101400	-0.09446600	-0.09224900
C	5.57264500	-1.31050300	0.07255200
C	6.96310900	-1.32630700	0.09705600
C	7.70856900	-0.14531100	-0.03699500
C	7.01963300	1.06262300	-0.19890600
C	5.62654800	1.08880000	-0.22808000
S	9.48811200	-0.29888300	0.01038300
C	10.07436100	1.41818800	-0.16634900
C	-2.90508200	-1.95850700	0.15784100
C	-2.85077800	-0.93184200	1.08572300
N	-3.86257000	-0.12193500	1.47629700
C	-5.11005900	-0.03358200	0.78634100
C	-3.71390700	0.63110700	2.72771300
C	-5.14565700	0.15689100	-0.60431600
C	-6.36032600	0.28850200	-1.26264300
C	-7.57435800	0.24323300	-0.55305900
C	-7.53484400	0.06301300	0.83480800
C	-6.31384800	-0.07415300	1.49649900
S	-9.06808600	0.42666700	-1.50657900
C	-10.39147400	0.39899500	-0.25302800
C	0.61535400	2.11385600	-0.13292200
F	-0.11469900	2.10248800	1.01569100
F	-0.28553000	2.04436800	-1.14667900
F	1.22719700	3.30355700	-0.21036900
H	-1.98497100	-3.70071400	-0.64873500
H	-0.85019400	-0.28053500	-0.28016100
H	1.38120700	-3.05405000	-0.38800400
H	5.01669200	-2.23185400	0.17566300
H	7.47264500	-2.27804400	0.22561900
H	7.55437600	1.99982700	-0.30744900
H	5.10558000	2.02997200	-0.35365700
H	11.16618200	1.36152500	-0.13526200
H	9.77359500	1.85233100	-1.12410900
H	9.73255600	2.04923000	0.65899000
H	-3.88020000	-2.28105400	-0.19838000
H	-1.92322100	-0.76393500	1.62612300
H	-2.65385700	0.83445100	2.89895600
H	-4.24726700	1.58042900	2.64637900
H	-4.10419900	0.07104600	3.58615300
H	-4.21963600	0.21942600	-1.16631500
H	-6.36582300	0.44544600	-2.33809600
H	-8.44827100	0.02105400	1.41732900

H	-6.30925700	-0.22457100	2.57163400
H	-11.32319200	0.53675700	-0.80891200
H	-10.43511900	-0.56030300	0.27017600
H	-10.28570200	1.21925600	0.46260000

Supplementary Table 27. Cartesian coordinates of optimized SDAS-TS (B'-C) in Gaussian package.

C	-2.43424100	1.16034400	-0.27637500	C	-7.43937300	-0.21838200	0.00567300
C	-1.26332600	-0.76633000	-0.09495400	H	-7.38882600	1.89651000	0.40815400
C	-1.08177900	0.63823800	-0.28529300	H	-7.11189800	-2.31692000	-0.39827000
C	0.13761500	1.29311900	-0.40004400	F	-0.60911100	-2.99615300	0.26380600
O	-2.84547000	2.31720200	-0.42641400	F	0.61144300	-1.79740400	-1.05202600
C	0.41880900	2.65787200	-0.56881500	F	0.65328400	-1.43707800	1.06704300
C	1.74377100	3.09760700	-0.56387400	N	3.56677600	0.65048000	1.51965000
C	2.82886600	2.41349700	-0.00027200	C	4.77724600	0.33998700	0.83812400
C	2.65968700	1.51191100	1.03434500	C	5.96808900	0.23255900	1.55552400
O	-0.52410100	3.59513500	-0.84214800	C	4.76871400	0.08776500	-0.53361000
H	1.71934500	1.52077900	1.57816500	C	7.14515600	-0.09656000	0.89812300
H	3.83208000	2.63948400	-0.34610600	H	5.98253000	0.42801900	2.62131200
H	1.92312100	4.06188200	-1.03207100	C	5.94638400	-0.25379400	-1.18178500
H	-1.43209900	3.22463500	-0.77521500	H	3.83715900	0.13263500	-1.08606200
H	1.00929200	0.65062900	-0.42958900	C	7.14498800	-0.34427100	-0.47366900
C	-0.16608500	-1.76584800	0.04390100	H	8.07341200	-0.14693000	1.45600900
N	-2.50298500	-1.11716400	0.03336100	H	5.93281800	-0.46756300	-2.24375500
N	-3.23160400	0.03933500	-0.08321500	S	8.65698500	-0.74967400	-1.32880500
C	-4.64717800	-0.02933400	-0.05248500	C	8.99102700	-2.37197600	-0.57709500
C	-5.41906800	1.10798500	0.19577500	H	8.16832300	-3.05804500	-0.77072200
C	-5.27324000	-1.25334200	-0.26531500	H	9.89712400	-2.75640200	-1.04452700
C	-6.79994000	1.00557700	0.21772200	H	9.15947900	-2.28055200	0.49486400
H	-4.94499000	2.06349200	0.36249200	S	-9.21171000	-0.22163500	0.06328500
C	-6.65869500	-1.34814900	-0.23169900	C	-9.60898000	-1.94638100	-0.29500200
H	-4.67466900	-2.13460600	-0.45150500	H	-10.69640600	-2.00898500	-0.28645800

H	-9.24986900	-2.24052000	-1.28071200
H	-9.21539300	-2.61444000	0.47033200
C	3.33928000	0.03225300	2.82605600
H	3.71505100	-0.98943800	2.81178300
H	3.83461700	0.59007000	3.62347100
H	2.26976500	0.00958600	3.02751100

Supplementary Table 28. Cartesian coordinates of optimized SDAS-C in Gaussian package.

C	0.30434247	-0.07266052	-0.35653941	N	-2.21916250	-1.25683008	1.72925546
C	0.55331769	1.31607853	-0.42751612	C	-1.55444641	-0.27501728	2.57963245
N	1.82690256	1.63229566	-0.30404085	S	8.36333881	0.20292707	0.65610827
N	2.45310826	0.44686381	-0.17131078	C	8.95677612	-1.08432679	-0.46461724
C	1.56985940	-0.58765692	-0.18697536	C	-0.45657225	2.40307247	-0.58172678
O	1.90892314	-1.87871044	-0.06310363	F	-1.14864714	2.60272017	0.56109652
C	3.86223293	0.37426482	-0.00532519	F	-1.36689946	2.10108803	-1.51869909
C	4.49622141	1.24839946	0.87625803	F	0.09709356	3.56718436	-0.90775267
C	5.86626377	1.17357220	1.04000653	H	2.79813117	-1.97464452	0.30632513
C	6.62163593	0.21712537	0.34538746	H	3.90892763	1.98507118	1.41097865
C	5.97589913	-0.64744615	-0.53678955	H	6.35946305	1.86029975	1.71968557
C	4.59852985	-0.56085534	-0.72184260	H	6.52956438	-1.38208099	-1.10684790
C	-0.95526717	-0.86208966	-0.47106433	H	4.11087413	-1.19471323	-1.45515233
C	-0.95055653	-1.80189050	-1.69246616	H	-1.80550902	-0.18770079	-0.62539961
C	-1.60183469	-3.06319895	-1.27278057	H	-1.81319965	-3.86962661	-1.96313413
C	-1.80508912	-3.05281714	0.04441656	H	-2.22659575	-3.86431879	0.62652243
C	-1.30623315	-1.79830158	0.72044465	H	-0.38549529	-2.05949670	1.25423121
O	-0.47219976	-1.55102878	-2.76666391	H	-4.24014125	-2.87607458	1.18477096
C	-3.49953978	-0.85932603	1.23840169	H	-6.44531952	-2.27594857	0.29488990
C	-4.46611963	-1.83769862	0.97388600	H	-5.32919686	1.87510615	0.39617667
C	-5.71527860	-1.49614751	0.48706642	H	-3.12938965	1.26425472	1.21985245
C	-6.05304138	-0.15585743	0.26588954	H	-8.68977936	2.22820464	-0.87428465
C	-5.10802174	0.82602812	0.54845405	H	-7.56853686	2.45042164	0.47803292
C	-3.84492920	0.47470651	1.02210255	H	-6.95179914	2.31945730	-1.19930358
S	-7.68713342	0.16936373	-0.34601122	H	-2.24996122	0.06739169	3.34666941
C	-7.70277553	1.97031308	-0.49108144	H	-0.70960651	-0.75714942	3.07592588

H	-1.17375547	0.60048558	2.03886312
H	10.03701675	-1.11865511	-0.32604226
H	8.54254987	-2.05966613	-0.21040374
H	8.74658310	-0.83421572	-1.50422529

Supplementary Table 29. Cartesian coordinates of optimized SSDAS-A in Gaussian package.

N	4.28642800	-0.00440600	0.17367600
C	3.12364900	-0.69664300	0.19924600
C	1.85579400	-0.18444000	0.10612300
C	0.74845200	-1.04756700	0.19600800
C	-0.56680500	-0.62421800	0.09600300
C	-1.57307700	-1.60027800	0.19960300
C	-2.95024000	-1.46303200	0.13414000
O	-0.78371200	0.69140100	-0.09313300
C	5.52819300	-0.69125800	0.04337100
C	4.92345600	2.16664200	-0.74694700
C	4.30190600	1.42645500	0.24978500
C	4.96734800	3.55401600	-0.66381900
C	4.38213700	4.21224700	0.41842000
C	3.75597100	3.45609400	1.41799500
C	3.72066800	2.07530400	1.33883900
C	-3.92732400	-2.50619100	0.25302100
N	-5.14465700	-2.08596800	0.14864700
N	-5.06828300	-0.73232100	-0.04994700
C	-3.76078300	-0.26769100	-0.06511300
O	-3.42402500	0.90956500	-0.21448900
C	-6.27075500	0.00985100	-0.16554000
C	-6.27016500	1.32777400	-0.63075800
C	-7.46743800	2.01728000	-0.73530000
C	-8.68202300	1.41971200	-0.38872900
C	-8.67089900	0.10352300	0.06987300
C	-7.47554100	-0.59542500	0.18292600
S	4.37543300	5.96941800	0.62986000
C	5.26354900	6.55097100	-0.83240300
C	6.65159400	-0.24433000	0.74566200
C	7.85123900	-0.92163100	0.63621100
C	7.96322700	-2.06160300	-0.17151100
C	6.84399900	-2.49536400	-0.87879200
C	5.63828900	-1.80711600	-0.78008500
S	9.54923400	-2.84787700	-0.22768900
C	9.24710400	-4.27388600	-1.29555400
S	-10.14641600	2.40687400	-0.56540400
C	-11.45763500	1.25353500	-0.10105000
C	-3.65482800	-3.95892800	0.46773500
F	-2.93825700	-4.15659400	1.58813500
F	-2.92507200	-4.47063800	-0.53867300
F	-4.76807400	-4.67612000	0.56672000
H	3.25251500	-1.76864000	0.31630800
H	1.68370800	0.87503000	-0.03930900
H	0.92329600	-2.10765700	0.35030900
H	-1.19749000	-2.60690700	0.35540000
H	-1.74343400	0.90018400	-0.15055100
H	5.37828100	1.65825700	-1.59023200
H	5.45680200	4.10709700	-1.45501800
H	3.30010000	3.95376100	2.26732600
H	3.24159400	1.49444100	2.11921600
H	-5.34329100	1.80856800	-0.90479200
H	-7.45260300	3.03916100	-1.10020200

H	-9.58773000	-0.39959700	0.34982100
H	-7.47680600	-1.61720700	0.53789900
H	5.29251700	7.63680300	-0.74564600
H	6.28649500	6.17592000	-0.85400300
H	4.73660500	6.28898500	-1.74948200
H	6.57840400	0.62885900	1.38382000
H	8.71265000	-0.56884900	1.19362800
H	6.89488300	-3.35797000	-1.53079600
H	4.78818200	-2.13207700	-1.36976900
H	10.19191900	-4.81496600	-1.33883800
H	8.48774500	-4.93195800	-0.87372300
H	8.97457500	-3.96826700	-2.30537200
H	-12.39129600	1.80282900	-0.21992900
H	-11.47647400	0.38634900	-0.76098600
H	-11.36744100	0.94171400	0.93919800

Supplementary Table 30. Cartesian coordinates of optimized SSDAS-TS (A-B) in Gaussian package.

C	-3.73128300	-0.74806700	-0.47504600
C	-3.50432600	-1.42472800	1.62975100
C	-2.77512200	-1.24267700	0.44072600
C	-1.36762900	-1.50643800	0.20145800
O	-3.61311100	-0.42502800	-1.69367100
C	-0.64762800	-1.09918900	-0.85300300
C	0.79491400	-1.42362600	-0.97100300
C	1.74947800	-0.55565900	-0.58824200
C	3.11515800	-0.91705500	-0.78036700
O	-1.04861300	-0.34945300	-1.91208300
H	3.33895200	-1.91646200	-1.14150800
H	1.48623300	0.39528000	-0.14010500
H	1.08139800	-2.36701200	-1.43648800
H	-2.05741600	-0.31450300	-1.93091600
H	-0.83948600	-2.09010100	0.94978600
C	-2.95243800	-1.93545100	2.91184000
N	-4.77221200	-1.09799400	1.54468200
N	-4.92215200	-0.67917400	0.25341600
C	-6.18685000	-0.22801100	-0.17419000
C	-6.44444800	0.06416000	-1.51703800
C	-7.20401400	-0.07208000	0.77351000
C	-7.70629800	0.50646400	-1.89295200
H	-5.66191900	-0.05301600	-2.25048600
C	-8.45810300	0.36755600	0.38138000
H	-6.99745500	-0.29799400	1.80990900
C	-8.72530100	0.65957800	-0.95609100
H	-7.89950600	0.73546600	-2.93504000
H	-9.23873100	0.49142500	1.12372200
F	-3.85734000	-1.99918800	3.88692900
F	-2.43093300	-3.17253700	2.77138700
F	-1.93890100	-1.16552800	3.35797900
C	5.48428200	-0.70177000	-0.62252200
C	5.75901300	-1.93097700	-0.04106400
C	7.75145000	-0.52228600	-1.36987300
C	7.03656400	-2.46924300	-0.13649800
H	4.98998500	-2.45976300	0.51137400
C	8.04394800	-1.77444800	-0.80729100
H	8.52625500	0.02911600	-1.89055000
H	7.23058700	-3.42381800	0.33409600
N	4.15242200	-0.15584100	-0.55301600
C	6.48437800	0.01714900	-1.27563900
H	6.26427500	0.98177500	-1.71797900
S	9.69665500	-2.35717500	-0.99542400
C	9.64179600	-3.97707100	-0.19876100
H	8.92748500	-4.63662800	-0.68993700
H	10.64046500	-4.39495200	-0.31722500
H	9.42380200	-3.89221700	0.86503100
C	4.02390100	1.24869600	-0.24565400
C	4.66679500	1.76053600	0.87184700
C	3.29099900	2.07580300	-1.09280700

C	4.55277700	3.11131200	1.16863300
H	5.25037900	1.10816900	1.51147400
C	3.18189000	3.42105900	-0.79521000
H	2.81116100	1.67017600	-1.97665100
C	3.80592400	3.95467100	0.34083400
H	5.05156500	3.49169600	2.05004800
H	2.60591100	4.06457300	-1.45016100
S	3.59530300	5.68146400	0.61920600
C	4.44149200	5.93536100	2.19433500
H	4.30404200	6.98903700	2.43310900
H	3.99038100	5.33722800	2.98495600
H	5.50869200	5.73281500	2.11477500
S	-10.34571800	1.21482000	-1.45798800
C	-11.10830500	-0.40615100	-1.77801700
H	-11.12085800	-1.00873400	-0.87073600
H	-12.13302300	-0.22645300	-2.10358100
H	-10.56915400	-0.93315800	-2.56411200

Supplementary Table 31. Cartesian coordinates of optimized SSDAS-B in Gaussian package.

C	-0.92859200	-2.23773900	-0.22406300	C	-7.27878700	-2.50776900	0.59957200
C	0.45392700	-2.13780500	-0.17621600	C	-5.94838300	-2.09975500	0.57794400
C	1.06720300	-0.87844900	-0.03668400	C	-4.33564600	1.61055300	0.99403800
C	2.40546500	-0.53528600	-0.02094200	C	-3.98506400	2.94731400	1.08750700
O	1.13338700	-3.29701300	-0.32156500	C	-3.18719000	3.54604700	0.10510800
C	2.94240300	0.79246900	0.08291300	C	-2.77354200	2.78531900	-0.99019300
N	4.23320600	0.82686100	0.07160200	C	-3.13551300	1.44753000	-1.08854100
N	4.65352400	-0.47257300	-0.04231800	S	-9.98055700	-2.16956800	-0.07579900
C	3.59916000	-1.37332700	-0.09939500	C	-10.02287500	-3.75531800	0.78964500
O	3.70843500	-2.59632700	-0.18950400	S	-2.77063700	5.25097400	0.33565700
C	6.04528200	-0.74462600	-0.03767200	C	-1.47976100	5.51069100	-0.90454700
C	6.54304200	-1.98399000	-0.44880700	C	2.16017300	2.06152300	0.18670800
C	7.90956600	-2.21128500	-0.43382000	F	1.34895000	2.04719600	1.25722200
C	8.80529300	-1.22107200	-0.02153700	F	1.36063200	2.22969600	-0.88434000
C	8.29745700	0.01319600	0.38039600	F	2.93878200	3.13271300	0.28026900
C	6.92830500	0.24908300	0.37596300	H	-1.30083300	-3.24048800	-0.42730000
S	10.53102800	-1.63239100	-0.05083400	H	0.37907500	-0.04541000	0.04811700
C	11.30794900	-0.11591200	0.55098900	H	2.10422000	-3.16208900	-0.27847700
C	-1.88384500	-1.21515100	-0.05103300	H	5.86871500	-2.76303000	-0.77138100
C	-3.21658200	-1.46597500	-0.22622700	H	8.28337200	-3.17825200	-0.75445300
N	-4.21212000	-0.54329700	-0.16433900	H	8.95489100	0.80898300	0.70671700
C	-5.57421300	-0.94780000	-0.10772000	H	6.54304800	1.21038000	0.68868300
C	-3.89861700	0.85267400	-0.09047500	H	12.37942100	-0.31460300	0.55730800
C	-6.55587500	-0.18305100	-0.74611500	H	11.11360800	0.72446900	-0.11513200
C	-7.87809400	-0.58346800	-0.71229800	H	10.99119900	0.11876000	1.56702300
C	-8.25842400	-1.75607600	-0.04567500	H	-1.57488700	-0.21141500	0.21091300

H	-3.55188800	-2.47266900	-0.45610400
H	-6.27722300	0.71948500	-1.27802800
H	-8.62669200	0.01556300	-1.22028800
H	-7.53397500	-3.40686200	1.14588200
H	-5.20694800	-2.67445000	1.12191000
H	-4.93549000	1.14560900	1.76888700
H	-4.31187100	3.52673200	1.94448000
H	-2.17074300	3.22098400	-1.77670300
H	-2.80951500	0.85686800	-1.93797100
H	-11.06339000	-4.07787400	0.76329000
H	-9.41337500	-4.50175600	0.28066700
H	-9.71716600	-3.65318600	1.83055900
H	-1.09476600	6.51402400	-0.72406400
H	-0.66726200	4.79527800	-0.77797700
H	-1.87631100	5.46878400	-1.91833000

Supplementary Table 32. Cartesian coordinates of optimized SSDAS-TS (B-B') in Gaussian package.

C	-3.57891200	-0.60542800	1.04591800	C	-8.54295200	-0.41459100	-0.43883200
C	-2.43433100	0.60459300	-0.52098800	H	-8.59302100	-0.96914200	1.63432200
C	-2.21325000	-0.17841200	0.67667700	H	-8.16815900	0.20029700	-2.46328600
C	-1.01559800	-0.42252200	1.26500900	F	-1.88459000	1.92314800	-2.38161300
O	-3.95695000	-1.30389000	1.97487100	F	-0.69000200	2.16068000	-0.59836900
C	-0.68741800	-1.17663700	2.45265500	F	-0.48974200	0.38347500	-1.79261000
C	0.60760700	-1.23342100	2.84339100	C	4.61131300	-1.36292000	0.05869600
C	1.73496200	-0.55776100	2.16996600	C	4.95505000	-2.58121900	0.65372200
C	2.52401200	-1.18656700	1.29138600	C	6.37451900	-1.69032800	-1.56026700
O	-1.61845700	-1.82143700	3.19589400	C	5.98711900	-3.34996200	0.13043300
H	2.36943800	-2.23809100	1.06863200	H	4.43815000	-2.91880800	1.54399200
H	1.91402600	0.48834900	2.40866300	C	6.71005500	-2.91428600	-0.97660200
H	0.80072400	-1.79323200	3.75684900	H	6.91999900	-1.34638300	-2.43203800
H	-2.52192500	-1.71501800	2.84020000	H	6.24343000	-4.29249000	0.60064900
H	-0.14267500	0.01478000	0.78864800	N	3.54495100	-0.59295800	0.55454500
C	-1.38176700	1.28019500	-1.33992700	C	3.56334600	0.82720100	0.41233500
N	-3.67104600	0.67682600	-0.85632200	C	4.60509400	1.58041500	0.93520200
N	-4.38683700	-0.03555100	0.07737500	C	2.51832900	1.46950800	-0.25390900
C	-5.79149900	-0.16807500	-0.08144200	C	4.61399500	2.96568900	0.79452900
C	-6.59472100	-0.55225200	0.99132800	H	5.41835000	1.08147400	1.45114000
C	-6.35647200	0.10244500	-1.32708600	C	2.51322500	2.84640000	-0.38018900
C	-7.96514200	-0.67523600	0.80131500	H	1.71468000	0.87892100	-0.68012800
H	-6.16138500	-0.75159600	1.95978700	C	3.56491500	3.61138600	0.14206500
C	-7.72726300	-0.01696400	-1.49736400	H	1.69212600	3.33296500	-0.89557700
H	-5.72634400	0.41182900	-2.14956000	S	-10.30585000	-0.58041500	-0.66871800

C	-10.34631000	-2.32254700	-1.19391100
H	-9.94991200	-2.96774900	-0.41124400
H	-11.38956400	-2.57933800	-1.37620900
H	-9.77831700	-2.45924500	-2.11307200
S	8.04135600	-3.90071100	-1.63729000
C	9.43694600	-3.09281000	-0.79405400
H	10.34577800	-3.60546400	-1.10894800
H	9.50266900	-2.04343300	-1.07871000
H	9.33261000	-3.17944200	0.28655900
C	5.34132700	-0.92079900	-1.05321700
H	5.08183500	0.01805400	-1.52807700
H	5.44203100	3.52439900	1.21121700
S	3.45847300	5.36452100	-0.08474800
C	4.98109400	5.95460400	0.68586500
H	4.97316300	7.03705600	0.56511200
H	5.00587300	5.72297900	1.75001000
H	5.86189800	5.55447800	0.18499000

Supplementary Table 33. Cartesian coordinates of optimized SSDAS-B’ in Gaussian package.

C	-0.98422000	-2.16399400	-2.50688500
C	0.35047200	-1.90074700	-2.15785400
C	0.67374100	-1.34592300	-0.90051300
C	1.91916700	-1.01940800	-0.36095200
O	1.27249400	-2.32705100	-3.04883900
C	2.17787000	-0.51182600	0.95633400
N	3.44284100	-0.27067800	1.18044500
N	4.11021400	-0.62146400	0.02445900
C	3.24477000	-1.09022500	-0.96466400
O	3.58880500	-1.48720100	-2.09828500
C	5.52413400	-0.48593100	-0.01228100
C	6.25504300	-0.73183600	-1.18533700
C	7.63794000	-0.58392200	-1.18248700
C	8.32731800	-0.18954300	-0.02595900
C	7.58946200	0.05408800	1.13860800
C	6.20361700	-0.09273700	1.14684900
S	10.10289100	-0.03618100	-0.15184000
C	10.60750600	0.51696600	1.51029300
C	-2.17388500	-1.62079900	-1.98022400
C	-2.26706100	-0.39194500	-1.35627700
N	-3.37683400	0.11836500	-0.75154300
C	-3.47425600	1.52415500	-0.51388300
C	-4.47628100	-0.72892700	-0.37824400
C	-4.08547300	2.00238200	0.65609200
C	-4.15609200	3.36624300	0.90004900
C	-3.61568600	4.29296000	-0.01023000
C	-3.01099000	3.81212200	-1.17785400
C	-2.94979400	2.44143500	-1.43123700
C	-5.75706300	-0.48643000	-0.89105100
C	-6.82088500	-1.29968500	-0.52070100
C	-6.62920300	-2.37352600	0.36710800
C	-5.34475500	-2.60894200	0.87797200
C	-4.27832100	-1.78963300	0.50944700
S	-3.77176900	6.01677700	0.41299600
C	-2.88355500	6.87599300	-0.92724500
S	-8.06789800	-3.34461100	0.76772600
C	-7.42921500	-4.63584200	1.88526500
C	1.16511200	-0.19379900	2.01061600
F	0.37590500	-1.26801300	2.27461500
F	0.32719500	0.79523300	1.60563200
F	1.72617800	0.19431800	3.16416500
H	-1.09863600	-2.88271500	-3.31759900
H	-0.17296000	-1.21712000	-0.23686200
H	2.19441100	-2.07575600	-2.75677700
H	5.74335700	-1.03783700	-2.08699500
H	8.18571200	-0.77852700	-2.10124200
H	8.08010300	0.36002000	2.05607700
H	5.64500000	0.09970800	2.05443400
H	11.69435400	0.63148600	1.46570300
H	10.16348700	1.48308000	1.76647100
H	10.36480200	-0.22567600	2.27560400
H	-3.09078900	-2.17848900	-2.15475900

H	-1.41681700	0.28312900	-1.36622100
H	-4.49173900	1.30454900	1.38063800
H	-4.62483600	3.71636400	1.81598800
H	-2.59682800	4.49328500	-1.91271400
H	-2.51089700	2.09211700	-2.36096900
H	-5.91638200	0.33615800	-1.58145000
H	-7.80805700	-1.10384300	-0.93080000
H	-5.16050900	-3.42017400	1.57311800
H	-3.28981000	-1.97202800	0.92010300
H	-2.93269600	7.93939600	-0.67652500
H	-1.83352200	6.57387800	-0.97149100
H	-3.36530400	6.72298500	-1.89696800
H	-8.29269700	-5.25908300	2.13447100
H	-6.67699400	-5.25889000	1.39346300
H	-7.02729600	-4.20950600	2.80842700

Supplementary Table 34. Cartesian coordinates of optimized SSDAS-TS (B’-C) in Gaussian package.

C	2.41589800	-1.50251400	0.81715600
C	1.21138100	-1.04900900	-0.95879300
C	1.10384200	-1.60018100	0.34119100
C	-0.08822700	-2.09574400	0.97703100
O	2.95287100	-1.88256800	1.95644100
C	-0.09269300	-2.75720900	2.23668400
C	-1.43693900	-2.97564300	2.74549700
C	-2.35026700	-2.02078300	2.43915900
C	-1.85775400	-0.78417900	1.90448200
O	0.93909000	-3.08497200	2.89697300
H	-0.98131400	-0.33129700	2.34457900
H	-3.41897400	-2.14545100	2.58838500
H	-1.65458000	-3.83065600	3.37468600
H	2.23387600	-2.42944000	2.43808300
H	-0.93074200	-2.27052800	0.32607800
C	0.08062500	-0.89754800	-1.91192900
N	2.41781400	-0.64439700	-1.26837800
N	3.16105800	-0.92843300	-0.16724400
C	4.55714400	-0.65781500	-0.17056500
C	5.23156900	-0.35796800	1.01179200
C	5.24181600	-0.67479700	-1.37871500
C	6.58931500	-0.09084300	0.97283200
H	4.70103600	-0.33049700	1.95294800
C	6.60185400	-0.39482100	-1.41240500
H	4.70716400	-0.90006500	-2.29204500
C	7.29160100	-0.10491500	-0.23585400
H	7.10787700	0.14262700	1.89638600
H	7.10876900	-0.41553500	-2.36828600
F	0.43284600	-0.32399900	-3.05616200
F	-0.47641700	-2.08849600	-2.20610400
F	-0.91134400	-0.15759600	-1.37238500
C	-2.25096600	1.42782400	1.03186900
C	-1.75464300	2.13316200	2.12809500
C	-2.05372200	3.41253800	-0.32042900
C	-1.38946500	3.46134100	1.99092600
H	-1.67035400	1.65183000	3.09571100
C	-1.53200700	4.11818700	0.76488400
H	-2.17994000	3.88594700	-1.28525600
H	-1.00142700	3.99690400	2.85052700
N	-2.60689200	0.04879600	1.15941500
C	-2.40817200	2.07711600	-0.18803900
H	-2.79401000	1.53535400	-1.04312200
C	-3.76697900	-0.41947100	0.45822200
C	-4.98583000	0.21462800	0.66449300
C	-3.68273200	-1.48677600	-0.43389600
C	-6.12374400	-0.21800600	-0.00459400
H	-5.04446500	1.05292900	1.35000100
C	-4.81825700	-1.92400700	-1.09390800
H	-2.73063900	-1.96149800	-0.63491700
C	-6.05218600	-1.29697800	-0.88694100

H	-7.05917300	0.29471300	0.17733400
H	-4.74230300	-2.75361300	-1.78835400
S	-1.04226200	5.81759700	0.70432900
S	-7.43291300	-1.93670400	-1.78878200
C	-1.30612200	6.23770000	-1.03229500
H	-0.98726800	7.27378200	-1.13792300
H	-0.69563800	5.61592100	-1.68603900
H	-2.35822500	6.16733800	-1.30625400
C	-8.79032700	-0.88519100	-1.22902700
H	-8.62724600	0.15667400	-1.50190500
H	-9.67651700	-1.24660800	-1.74913600
H	-8.95269700	-0.98199700	-0.15620700
S	9.02545800	0.25238300	-0.15848600
C	9.51183800	0.21134800	-1.89716400
H	10.57444200	0.45032300	-1.91305000
H	9.37128600	-0.77892700	-2.32860100
H	8.97612000	0.96207700	-2.47714900

Supplementary Table 35. Cartesian coordinates of optimized SSDAS-C in Gaussian package.

C	-0.26748700	1.90823200	-0.15072600	N	-2.06262900	-0.42031400	-1.61469000
C	-0.04321500	2.03212500	1.23644500	C	-3.26872700	-0.81712600	-1.02169800
N	1.18742900	1.73758500	1.59945600	C	-3.45571000	-2.17069100	-0.68673500
N	1.81561100	1.43450500	0.45230900	C	-4.64717500	-2.61036000	-0.13741300
C	0.96734500	1.51133300	-0.61512500	C	-5.69960500	-1.72328000	0.10129300
O	1.34808100	1.21258400	-1.87005700	C	-5.52547300	-0.38614700	-0.24058400
C	3.15462700	0.95412400	0.47912700	C	-4.33120600	0.06841700	-0.78902300
C	3.59436200	0.26298600	1.60770500	S	-7.23386100	-2.29383600	0.81054600
C	4.88421300	-0.23252500	1.64748200	C	-6.81919000	-2.13857400	2.57699300
C	5.74947000	-0.06804500	0.55901900	S	7.35648100	-0.79493200	0.70578300
C	5.30604200	0.64477500	-0.55294200	C	8.10676200	-0.42376500	-0.89545000
C	4.01524700	1.16426300	-0.59139700	C	-1.07064400	2.35810900	2.26639300
C	-1.57230400	1.98482300	-0.86841100	F	-1.95092700	1.34246700	2.40298200
C	-1.93764600	3.31646100	-1.54434000	F	-1.79216700	3.43213900	1.92200600
C	-2.82205100	2.96733600	-2.68779200	F	-0.53882200	2.58196200	3.46450600
C	-2.78565800	1.65063100	-2.90737800	H	1.69532500	0.30571800	-1.90967300
C	-1.78981900	0.95756900	-2.00806700	H	2.92370100	0.13728100	2.44879300
O	-1.57235400	4.41013300	-1.20819300	H	5.22272900	-0.76637600	2.52981200
C	-0.86375100	-1.13975600	-1.29665500	H	5.95596900	0.81747600	-1.40103600
C	-0.03048200	-1.57026600	-2.32713100	H	3.69584200	1.74543700	-1.44691800
C	1.21471700	-2.12061600	-2.04573400	H	-2.37499000	1.81946700	-0.13865900
C	1.64741800	-2.25980700	-0.72070100	H	-3.36561400	3.71074100	-3.25657900
C	0.79071000	-1.86718900	0.31388600	H	-3.32286800	1.11130300	-3.68001500
C	-0.45168600	-1.31580500	0.02384800	H	-0.86332300	0.90745700	-2.58489300
S	3.27582800	-2.91027500	-0.47141200	H	-0.35786400	-1.45617800	-3.35543000
C	3.26258400	-3.35171700	1.28093600	H	1.84929900	-2.45813700	-2.85896500

H	1.08913900	-1.96405100	1.35075700
H	-1.09332200	-0.97905700	0.83225200
H	4.22328400	-3.83080900	1.47100900
H	3.18227900	-2.47384700	1.91935400
H	2.46358700	-4.06087700	1.49693100
H	-2.66269100	-2.88385100	-0.87734100
H	-4.76910500	-3.66177900	0.09999800
H	-6.33194500	0.31920600	-0.07204700
H	-4.25181900	1.11771700	-1.03696800
H	-7.69410000	-2.46050900	3.14266100
H	-5.97443300	-2.77765200	2.83215800
H	-6.59151100	-1.10328700	2.82836400
H	9.09613900	-0.87958200	-0.87088300
H	7.53740100	-0.86706000	-1.71211500
H	8.22226900	0.64956000	-1.0431430

9 References

1 Xu, W. *et al.* A Peierls transition in long polymethine molecular wires: Evolution of molecular geometry and single-molecule conductance. *J. Am. Chem. Soc.* **143**, 20472-20481 (2021).

2 Clerc, M. *et al.* Promoting the Furan ring-opening reaction to access new donor-acceptor Stenhouse adducts with Hexafluoroisopropanol. *Angew. Chem. Int. Ed.* **60**, 10219-10227 (2021).

3 Frisch, M. e. *et al.* (Gaussian, Inc. Wallingford, CT, 2016).

4 Grimme, S., Antony, J., Ehrlich, S. & Krieg, H. A consistent and accurate ab initio parametrization of density functional dispersion correction (DFT-D) for the 94 elements H-Pu. *J. Chem. Phys.* **132** (2010).

5 Grimme, S., Ehrlich, S. & Goerigk, L. Effect of the damping function in dispersion corrected density functional theory. *J. Comput. Chem.* **32**, 1456-1465 (2011).

6 Lu, T. & Chen, F. Multiwfn: A multifunctional wavefunction analyzer. *J. Comput. Chem.* **33**, 580-592 (2012).

7 Humphrey, W., Dalke, A. & Schulten, K. VMD: visual molecular dynamics. *J. Mol. Graph.* **14**, 33-38 (1996).

8 Stricker, F. *et al.* A multi-stage single photochrome system for controlled photoswitching responses. *Nat. Chem.* **14**, 942-948 (2022).

9 Stricker, F. *et al.* Selective control of donor-acceptor Stenhouse adduct populations with non-selective stimuli. *Chem* **9**, 1994-2005 (2023).

10 Adak, O. *et al.* Flicker noise as a probe of electronic interaction at metal-single molecule interfaces. *Nano Lett.* **15**, 4143-4149 (2015).

11 Tang, C. *et al.* Reversible switching between destructive and constructive quantum interference using atomically precise chemical gating of single-molecule junctions. *J. Am. Chem. Soc.* **143**, 9385-9392 (2021).

12 Tang, C. *et al.* Multicenter-bond-based quantum interference in charge transport through single-molecule carborane junctions. *Angew. Chem.* **131**, 10711-10715 (2019).

13 Xu, X. *et al.* Manipulating π-π interactions between single molecules by using antenna electrodes as optical tweezers. *Phys. Rev. Lett.* **133**, 233001 (2024).

14 Brandbyge, M., Mozos, J.-L., Ordejón, P., Taylor, J. & Stokbro, K. Density-functional method for nonequilibrium electron transport. *Phys. Rev. B* **65**, 165401 (2002).

15 Perdew, J. P., Burke, K. & Ernzerhof, M. Generalized gradient approximation made simple. *Phys. Rev. Lett.* **77**, 3865 (1996).
